# Ambiphilic boryl groups in a neutral Ni(ii) complex: a new activation mode of H_2_†

**DOI:** 10.1039/d0sc06014c

**Published:** 2020-12-22

**Authors:** Pablo Ríos, Javier Borge, Francisco Fernández de Córdova, Giuseppe Sciortino, Agustí Lledós, Amor Rodríguez

**Affiliations:** Instituto de Investigaciones Químicas, Departamento de Química Inorgánica, Universidad de Sevilla, Consejo Superior de Investigaciones Científicas, Centro de Innovación en Química Avanzada (ORFEO-CINQA) C/Américo Vespucio 49 41092 Sevilla Spain marodriguez@iiq.csic.es; Departamento de Química Física y Analítica, Centro de Innovación en Química Avanzada (ORFEO-CINQA), Universidad de Oviedo C/Julián Clavería 8 33006 Oviedo Spain; Departament de Química, Centro de Innovación en Química Avanzada (ORFEO-CINQA), Universitat Autònoma de Barcelona Campus UAB 08193 Cerdanyola del Vallès Spain agusti@klingon.uab.es

## Abstract

The concept of metal–ligand cooperation opens new avenues for the design of catalytic systems that may offer alternative reactivity patterns to the existing ones. Investigations of this concept with ligands bearing a boron center in their skeleton established mechanistic pathways for the activation of small molecules in which the boron atom usually performs as an electrophile. Here, we show how this electrophilic behavior can be modified by the ligand *trans* to the boron center, evincing its ambiphilic nature. Treatment of diphosphinoboryl (PBP) nickel–methyl complex 1 with bis(catecholato)diboron (B_2_Cat_2_) allows for the synthesis of nickel(ii) bis-boryl complex 3 that promotes the clean and reversible heterolytic cleavage of dihydrogen leading to the formation of dihydroborate nickel complex 4. Density functional theory analysis of this reaction revealed that the heterolytic activation of H_2_ is facilitated by the cooperation of both boryl moieties and the metal atom in a concerted mechanism that involves a Ni(ii)/Ni(0)/Ni(ii) process. Contrary to 1, the boron atom from the PBP ligand in 3 behaves as a nucleophile, accepting a formally protic hydrogen, whereas the catecholboryl moiety acts as an electrophile, receiving the attack from the hydride-like fragment. This manifests the dramatic change in the electronic properties of a ligand by tuning the substituent *trans* to it and constitutes an unprecedented cooperative mechanism that involves two boryl ligands in the same molecule operating differently, one as a Lewis acid and the other one as a Lewis base, in cooperation with the metal. In addition, reactivity towards different nucleophiles such as amines or ammonia confirmed the electrophilic nature of the Bcat moiety, allowing the formation of aminoboranes.

## Abstract 2

A bis(boryl)nickel complex promotes the facile and reversible activation of H_2_ through a cooperative mechanism that involves the metal and both boryl moieties in a concerted five-center process.

## Graphical abstract

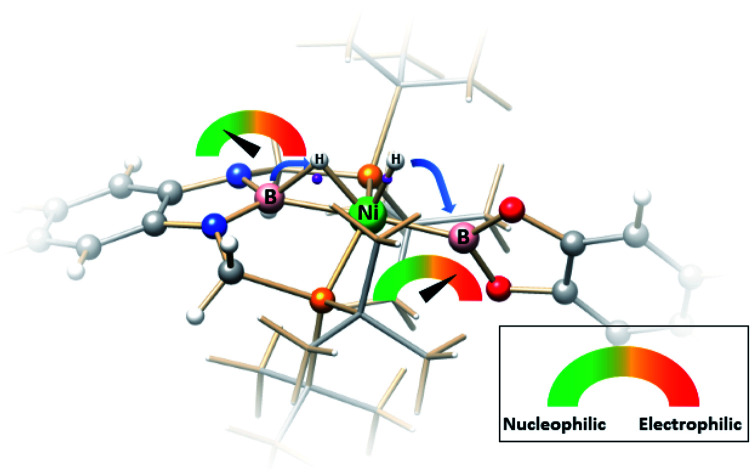

## Introduction

**Scheme 1:**
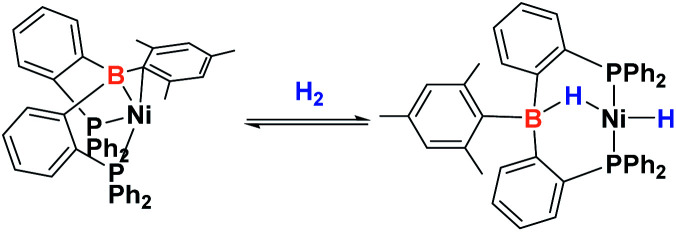
Activation of H_2_ by a nickel–borane complex.

Since the discovery of pincer ligands in the late 1970s,^1^ we have witnessed a tremendous expansion of their use as supporting ligands in many transition metal complexes that are involved in relevant catalytic transformations.^2,3^ More recently, inspired by nature, the metal–ligand cooperative processes observed in catalytic reactions mediated by enzymes have been mimicked based on ligand design to develop cooperative catalysts in which both, the metal and the ligand, play a part in the catalytic reaction.^4^ A wide variety of systems having diverse metal–ligand bonds have proven very effective for the activation of diverse small molecules.^5^ Recently, the growing interest in first-row transition metal catalysts has also motivated the design of cooperative platforms based on these metals.^6^ Among them, those systems that bear a boron atom in the ligand architecture, whether as a borane or as a boryl functionality, show a different reactivity pattern, the boron center acting as a Lewis acid, in contrast to that observed when electron-rich moieties such as amides, alkoxides or carbenes are involved (in these situations the Lewis acidic site resides on the metal).^7–11^ In 2012 Peters and co-workers^9^ reported the heterolytic cleavage of dihydrogen along with alkene hydrogenation by using a nickel–borane complex stabilized by a bis(phosphino)borane ligand previously described by the group of Bourissou (Scheme 1).^10^

**Scheme 2:**
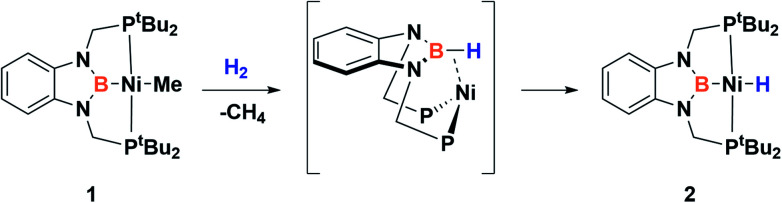
Activation of H_2_ by nickel–methyl complex 1.

Recently, we have described the activation of dihydrogen mediated by a nickel–methyl complex stabilized by a bis(phosphino)boryl pincer ligand.^11*c*^ Mechanistic studies suggest that the H_2_ bond heterolytic rupture involves a metal–boryl cooperation mechanism in which the boryl moiety acts as the electrophilic site accepting a hydride to form, after loss of methane, a Ni(0) σ-borane complex that finally evolves to a nickel(ii) hydride species 2 (Scheme 2).^11*a*^

At this point we wondered if the electrophilic behavior of this boron center might be affected by the nature of the group *trans* to it and, if possible, selecting the appropriate ligand would transform a Lewis acidic boron center into a nucleophilic one. Therefore, we envisioned that placing a stronger σ-donor like a dioxoboryl moiety (which on the other hand has an empty p orbital), instead of a methyl group, for the diaminoboryl fragment may have an influence on the electronic distribution on the B–Ni–L axis.

## Results and discussion

**Scheme 3:**
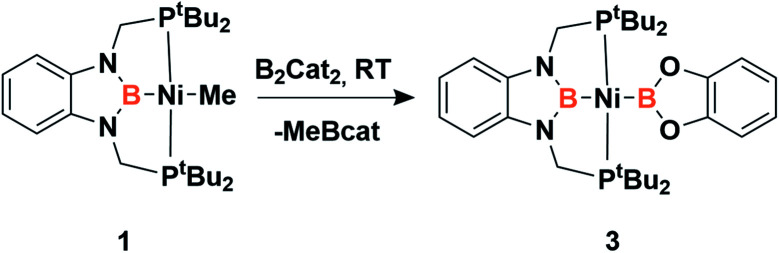
Synthesis of *trans*-bis(boryl)-nickel complex 3.

Accordingly, to get access to our target complex, the reaction of 1 with bis(catecholato)diboron was performed. We observed the instantaneous and clean formation of [(PBP)NiBcat] (3) with concomitant formation of 2-methyl-1,3,2-benzodioxaborole as the only by-product by ^1^H and ^11^B NMR (Scheme 3). It is worth mentioning that, up to now, the only nickel boryl complex fully characterized is the diphosphino–amido nickel complex [(PNP)NiBcat] reported by Mindiola and co-workers.^12^

**Fig. 1:**
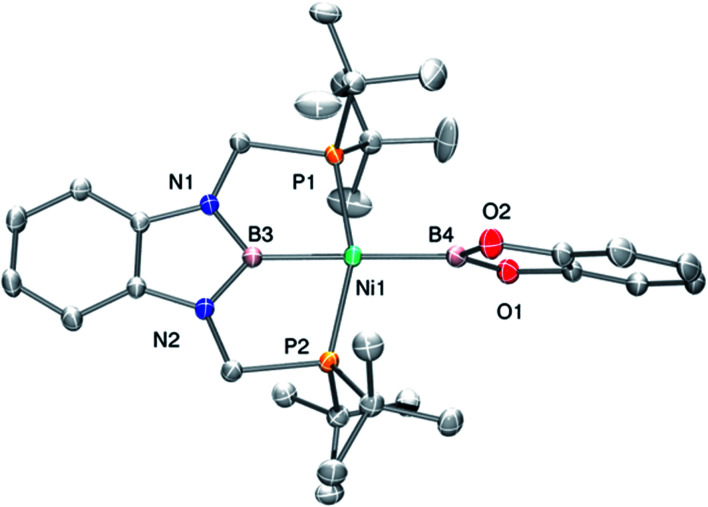
Molecular structure of 3 (H atoms omitted for clarity). Thermal ellipsoids are represented at the 50% probability level. Selected bond distances (Å) and angles (°): Ni(1)–B(4) = 2.015(2); Ni(1)–B(3) = 1.942(2); P(1)–Ni(1)–P(2) = 157.74(3); B(3)–Ni(1)–B(4) = 175.0(1).

Complex 3 displays only one ^31^P NMR signal (*δ* 117.3 ppm) according to its symmetry and two new resonances at 49 and 59 ppm in the ^11^B NMR spectrum for the two boryl moieties. This species shows a high sensitivity to oxygen and moisture, although appropriate crystals for X-ray diffraction analysis could be obtained by cooling a concentrated toluene/pentane solution of 3 (Fig. 1). The solid-state structure of 3 reveals a square-planar geometry at nickel with both boryl groups adopting a *trans* configuration due to the constraint exerted by the pincer scaffold. This arrangement is rather uncommon because of the tendency of boryl groups to occupy *cis* or *fac* positions in metal complexes. In fact, just a few examples of d-block compounds containing two mutually *trans* boryl ligands have been described.^13^ To the best of our knowledge, 3 is the first nickel complex of this type. 3 exhibits the Bcat fragment located almost perpendicular (N(1)–B(3)–B(4)–O(2) = 100.0(3)°) to the nickel square plane. The Ni(1)–B(3) bond distance (1.942(2) Å) is close in value to that observed in Mindiola's complex (1.9091(18) Å).^12^ However, the Ni(1)–B(4) bond (2.015(2) Å) is considerably longer as a consequence of the stronger *trans*-influence of the diaminoboryl moiety compared to the oxygen-containing one.^14^

**Fig. 2:**
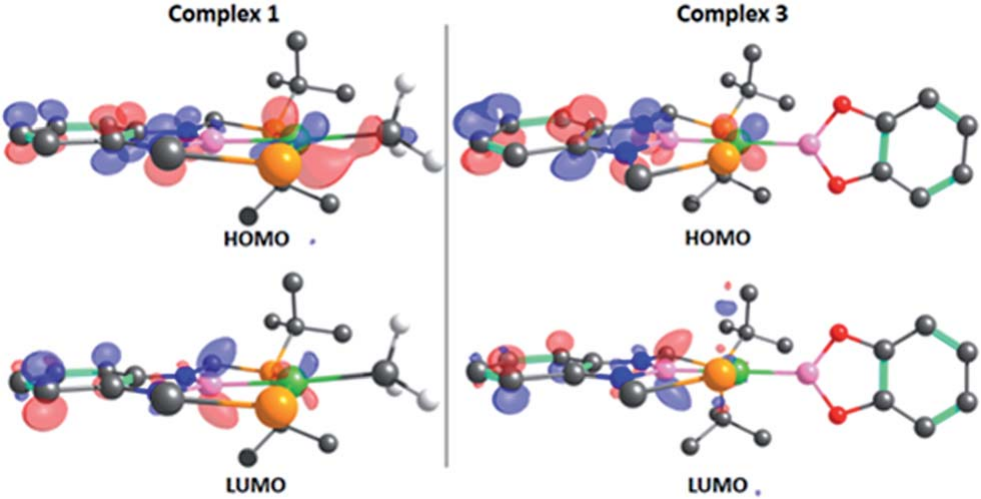
Frontier orbitals of complexes 1 (left) and 3 (right). Isodensity value: 0.06 e au^−3^. Most of the hydrogen atoms and some *tert*-butyl groups have been omitted for clarity.

The structure of 3 was analyzed by DFT methods using the PBE0/def2TZVP/def2QZVP level of theory, including Grimme's D3 (PBE0-D3) dispersion correction (see ESI† for more information and references), exhibiting an excellent agreement between the experimental and calculated geometries (Table S5†).^15^ A molecular orbital study was performed on the optimized structure of 3 in order to investigate its electron density and to determine whether the perpendicular conformation of the Bcat fragment might be due to the existence of metal (d)–boryl (p) π-interactions. As displayed in Fig. 2, the HOMO is located on the d_*xz*_ orbital of nickel and the π aromatic system of the PBP ligand (no overlap between Ni(1) and B(3) (PBP) is observed, given the opposite sign of the orbitals), whereas the LUMO is mainly located on the p_z_ orbital of B(3) (PBP). No appreciable contribution of the Bcat fragment is observed in any of them. Interestingly, methyl complex 1 exhibits almost identical frontier orbitals (Fig. 2, left); the main differences are the lack of contribution of B(3) (PBP) to the HOMO of 1 and the presence of electron density on the methyl group. This is in agreement with the electron rearrangement that we previously observed for the hydrogenolysis of the Ni–Me bond in 1 (*i.e.* the methyl moiety acts as a Lewis base capturing a proton while the boron atom behaves as a Lewis acid, receiving a hydride).^11*a*^ Additionally, the contribution of B(3) (PBP) to the HOMO in 3 clearly reveals the modification of the electron density along the B–M–L axis compared to 1, as discussed previously. Further analysis of 3 revealed the presence of energetically lower molecular orbitals with the right symmetry for π-backbonding, namely HOMO-6 and HOMO-7, although the coefficients of the p orbitals of the boron atoms in these molecular orbitals are considerably small (Fig. S58†).^16^

**Scheme 4:**
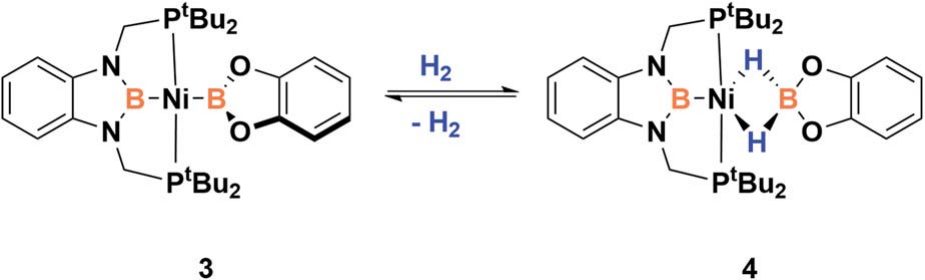
Dihydrogen activation by 3.

In order to explore the effect of this electronic picture on the cooperative bond activation of dihydrogen, we exposed a solution of 3 in C_6_D_6_ to an atmosphere of H_2_ (4 bar) at room temperature. We observed an immediate change of colour from yellow to pale orange upon diffusion of dihydrogen into the solution of 3. Analysis by ^1^H, ^31^P{^1^H} and ^11^B{^1^H} NMR spectroscopy confirmed the full conversion of 3 into a new species 4 (Scheme 4).

**Fig. 3:**
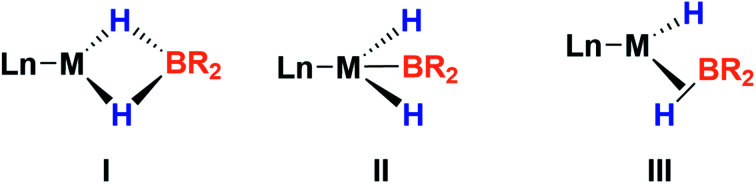
Different bonding scenarios in a MH_2_BR_2_ complex.

This new complex is characterized by a signal at 107.1 ppm in the ^31^P{^1^H} NMR spectrum. The ^11^B{^1^H} NMR spectrum shows two resonances; one is shifted upfield (18 ppm) compared to that observed for 3 (59 ppm), as expected for a tetra-coordinated boron center, while the other one appears at the typical chemical shift of a diaminoboryl group (41 ppm). The ^1^H NMR spectrum of 4 is very similar to that of 3, yet there is a singlet that integrates to two protons at *δ* = 1.49 ppm. These NMR data suggest the formation of a dihydro–borate complex (I, Fig. 3).^17^ The ^2^H NMR spectrum of a sample prepared by reacting 3 with deuterium shows a single broad resonance at the same chemical shift. Moreover, addition of a 1 : 1 mixture of H_2_ and D_2_ (2 bar) to a solution of 3 leads to the formation of HD, demonstrating the reversibility of the reaction (see ESI†).^18^

**Fig. 4:**
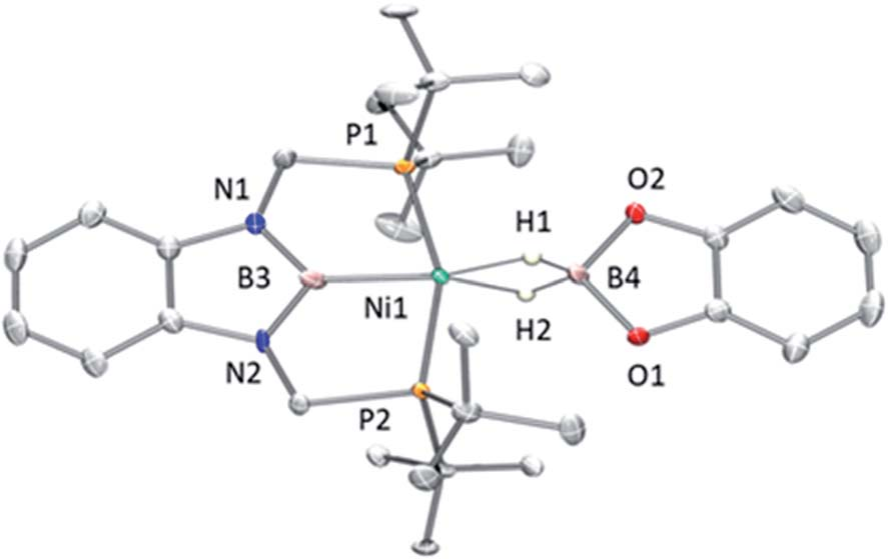
Molecular structure of 4 (H atoms omitted for clarity expect for the H_2_B unit). Thermal ellipsoids are represented at the 50% probability level. Selected bond distances (Å) and angles (°): Ni(1)–B(4) = 2.145(3); Ni(1)–B(3) = 1.966(2); B(4)–H(1) = 1.190(2); B(4)–H(2) = 1.267(3); Ni(1)–H(1) = 1.614(3); Ni(1)–H(2) = 1.750(2).

Fortunately, crystallization of 4 from C_6_D_6_/pentane at −20 °C afforded suitable crystals for X-ray diffraction analysis which confirmed the proposed structure by NMR (Fig. 4). Among the nickel dihydro–borate complexes reported, only a few have been characterized by X-ray diffraction analysis and, to our knowledge, 4 represents the first nickel complex containing a H_2_Bcat moiety.^19^ The structure of 4 shows a *κ*^2^ coordination mode for the H_2_Bcat group with two different B–H bond distances (1.190(2) and 1.267(3) Å). These distances are considerably shorter than those reported for niobocene Cp_2_Nb[BH_2_(O_2_C_6_H_4_)] (1.62(5) and 1.69(5) Å), whose structure resembles bonding scenario II (Fig. 3).^19*a*^ The Ni(1)–B(3) distance (1.966(2) Å) is significantly shorter than the Ni(1)–B(4) distance (2.145(3) Å) which is, in fact, longer than the sum of the covalent radii of nickel and boron (2.09 Å).^20^ The Ni–H bond distances (1.614(3) and 1.750(2) Å) are comparable to those found in similar species previously reported.^17^ Additional support for classifying 4 as a dihydroborate comes from QTAIM analysis on its optimized structure.^21^ Indeed, bond critical points (BCPs) were observed for Ni(1)–H(1), Ni(1)–H(2), B(4)–H(1) and B(4)–H(2), but not between Ni(1) and B(4). Moreover, a ring critical point (RCP) was also detected inside the kite-shaped cycle formed by the bond paths among Ni(1), H(1), H(2) and B(4) (Fig. S59†). These results are in good agreement with some previously reported complexes of osmium containing H_2_Bcat fragments.^19*f*^

At this point the question that arises is whether the mechanism of H_2_ activation is analogous to that operating when 1 is employed (*i.e.* the diaminoboryl group acting as an electrophile) or, in contrast, the presence of a dioxoboryl group *trans* to it affects its electronic behaviour to allow a different H–H bond activation mode. Accordingly, the mechanism for the formation of 4 was modelled by means of DFT calculations.^22^

**Fig. 5:**
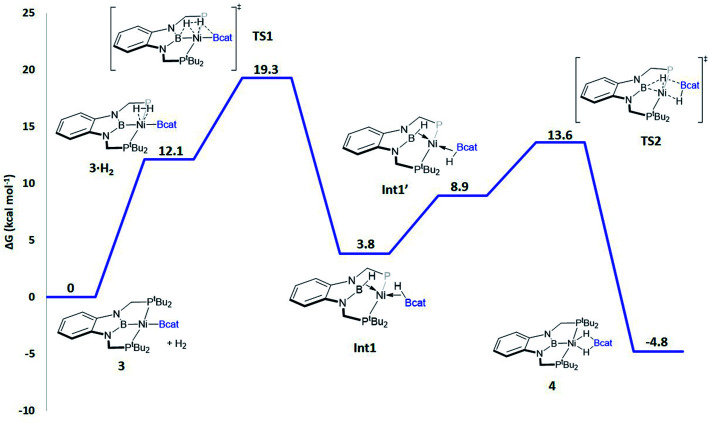
Gibbs energy profile in toluene for the reaction of 3 with H_2_. Relative Gibbs energies at 298 K and 1 M in kcal mol^−1^. Some of the phosphine groups (P^*t*^Bu_2_) have been abbreviated as P for clarity.

**Fig. 6:**
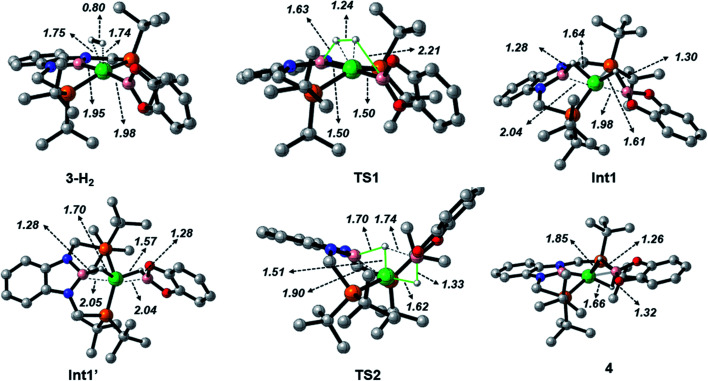
Optimized structures of intermediates and transition states in the Gibbs energy profile for the reaction of 3 with H_2_. Selected distances in Å. H atoms bonded to carbon atoms have been omitted for clarity.

When considering the potential mechanisms that could lead to the activation of the H_2_ molecule, several possibilities were taken into account: (a) decoordination of one of the phosphine ligands in order to create a vacant position where H_2_ could bind the metal atom; (b) cooperativity of the PBP pincer ligand, in a similar fashion to that reported previously^9,11^ (Schemes 1 and 2), and (c) metathesis processes.^23^ Decoordination of one of the phosphine groups turned out to be too energy demanding (the resulting product was 38.3 kcal mol^−1^ higher than complex 3), and exploratory calculations involving metathesis pathways evolved towards participation of the PBP ligand. Thus, the mechanism in which the pincer ligand assists the activation of the H_2_ was computed, as displayed in Fig. 5 (energy profile) and 6 (optimized structures of intermediates and transition states). Starting from 3, the approach of H_2_ to the pincer complex gives the corresponding adduct 3·H_2_, which is 12.1 kcal mol^−1^ above the origin. The increased energy and the small degree of elongation of the H–H bond (0.803 Å in 3·H_2_*vs.* 0.744 Å in the optimized free H_2_ molecule, see Fig. 6) reflect the weakness of the binding. This is not surprising, given the absence of a coordination vacant site in the complex, its neutral character and the electron configuration on the metal.^24^ In 3·H_2_ the dihydrogen ligand is slightly leaned towards the boron atom of the PBP ligand (H–B(PBP) = 2.387 Å, H–B(cat) = 2.407 Å), already anticipating the course of the reaction. The perpendicular orientation of the Bcat ligand precludes any interaction with H_2_ at this point. Although the formation of the dihydrogen complex seems to be unfavourable, H–H cleavage can take place easily from 3·H_2_ through TS1 (19.3 kcal mol^−1^). TS1 reveals substantial elongation of the H_2_ molecule (1.244 Å), where one of the H atoms is located close to the B atom of the PBP ligand (H–B(PBP) = 1.632 Å). In contrast, the other H atom is farther away from Bcat (H–B(cat) = 2.211 Å). Both hydrogen atoms remain close to Ni and almost equidistant (H–Ni = 1.495 and 1.503 Å). This geometrical arrangement of TS1 fits with an heterolytic cleavage of the H–H bond between a basic site (the boron atom of the PBP ligand) and a Lewis acid center (the Ni atom).^25*a*^ In agreement with this picture, natural charge analysis shows incipient charge separation between the two H atoms: whereas the one close to the PBP ligand possesses a charge of +0.023, the one next to Bcat has a charge of −0.052. Moreover, both boron atoms also differ in their natural charges (B(PBP) = +0.587 *vs.* B(cat) = +0.826). However, in contrast to what happens in usual ligand-assisted H–H heterolytic splitting, the hydride does not remain bonded to the metal; instead it ends up (Int1) connected to a more acidic center, namely the boron atom of the Bcat ligand. The metal is just assisting the heterolytic H–H scission between the two boron centers, which play different roles during H_2_ activation (see below).

Upon cleavage, each boryl moiety receives a H atom, giving the corresponding Ni(0) bis σ-BH complex Int1, with a relative Gibbs energy of 3.8 kcal mol^−1^. This species seems to contain two σ-BH interactions (average values: Ni–H ≈ 1.62 Å; B–H ≈ 1.29 Å; B–H–Ni ≈ 85°).^25*b*^ Interestingly, the Ni–B(cat) distance (1.979 Å) is slightly shorter than that of Ni–B(PBP) (2.037 Å). The explanation for this shortening might be found in the small degree of pyramidalization of the boron atom of the B(cat) unit (sum of angles around B(cat) = 343.9° *vs.* 351.7° in B(PBP)), suggesting a stronger interaction with the metal center. However, QTAIM analysis only evinces BCPs for the B–H and Ni–H bonds, suggesting η^1^ interactions (Fig. S60†). After Int1 catecholborane rotates in a similar way to that described by Hartwig *et al.*,^26^ placing both H atoms in a *trans* arrangement in species Int1′ falling at 8.9 kcal mol^−1^ (Fig. 6). The most appreciable consequences of this rotation are the slight contraction of the Ni–H(Bcat) bond to 1.567 Å and the B–Ni–B angle from 179.1° to 133.0°. Closing this angle renders the complex with an appropriate geometry for H transfer from PBP to Bcat: the catecholboryl fragment is orienting the p_*z*_ orbital of boron in a way that can receive one H atom. Indeed, the H atom from the diaminoborane can bounce to the boron atom of Bcat through TS2, giving 4 as the final and most thermodynamically stable species of the profile (Fig. 6). The energy required for this transition state is considerably small (4.7 kcal mol^−1^ with respect to Int1′), presumably because of the preorganized geometry.^27^ In TS2 the hydrogen atom that is being transferred is almost halfway between both boron centres (H–B(PBP) = 1.703 Å, H–B(Bcat) = 1.735 Å) and remains close to the Ni atom (H–Ni = 1.515 Å). TS2 can be considered as the transition state for the hydride transfer between the two boron atoms, assisted by the nickel atom (see Fig. S51, S52 and S65†).

The importance of both boryl groups in hydrogen activation was further evidenced when the boryl group of the PBP ligand was replaced by a carbene fragment (similar to a benzimidazol-2-ylidene moiety), giving a transition state similar to TS1 with a much higher Gibbs energy (34.3 kcal mol^−1^, Fig. S54†). In this cationic TS1 a heterolytic H–H rupture also takes place, but the Bcat boron receives the formally protic hydrogen (H–B(Bcat) = 1.452 Å) and the hydride is kept bonded to the nickel centre (Ni–H = 1.500 Å). TS1 with the carbene ligand is a late transition state (H–H = 1.416 Å *vs.* 1.244 Å with the PBP ligand). In this way the energy barrier for the H–H rupture is much higher and in agreement with the Hammond postulate the resulting intermediate is not stabilized (just 0.1 kcal mol^−1^ below TS1), giving a highly endergonic profile.

To analyze the influence of the pincer ligand in the reaction, we have computed the energy profile on an unconstrained (PMe_3_)_2_(B(NH)_2_R)Ni(Bcat) complex (R = C_6_H_4_). A similar mechanism can operate for the H_2_ activation (see Fig. S55†). However, in the unconstrained complex the isomer with the two boryl ligands mutually *cis* is 8.7 kcal mol^−1^ more stable than the *trans* boryl isomer. The pincer ligand is forcing the system to adopt the *trans* disposition of the boryls required for the reaction to take place.

**Fig. 7:**
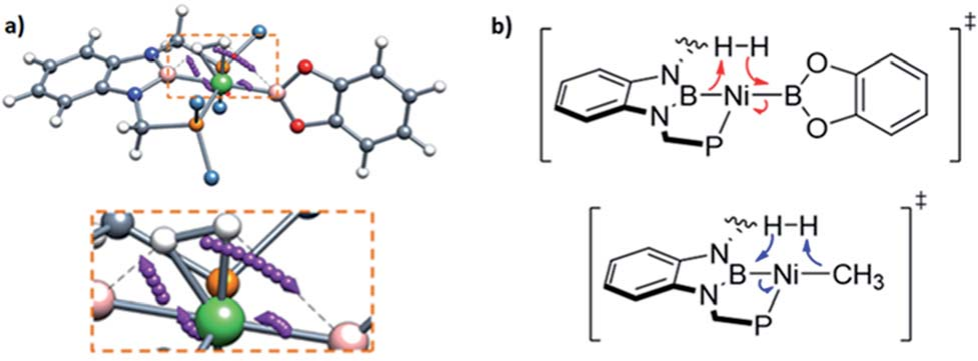
Electron rearrangement that takes place upon H_2_ activation in complex 3 (a) and comparison with complex 1 (b).^11^

In order to investigate the electron rearrangement that takes place upon exposure of 3 to H_2_ and compare it to the analogous reaction observed for methyl derivative 1, a localized orbital analysis was performed. This method involves the transformation of Kohn–Sham orbitals into maximally localized orbitals (LMOs) whose centroids are computed for the selected structures.^28^ In the case of methyl complex 1 and boryl species 3, a direct comparison of both species gives little information, as shown in Fig. S50;† nonetheless, studying the electron rearrangement along the reaction paths provides valuable information, since the rupture and formation of chemical bonds can be studied visually, similar to an arrow-pushing scheme. Thus, analysis of the centroids involved in the H–H cleavage in TS1 revealed that the boron atom from the PBP ligand acts as a Lewis base, capturing a formally protic hydrogen, whereas the catecholboryl moiety receives the attack from the hydride-like H fragment (Fig. 7a and b top). This is exactly the opposite result compared to that observed for complex 1, where the boryl fragment acts as a Lewis acid accepting a hydride-like unit and the methyl ligand behaves as a Lewis base, capturing a formally protic hydrogen (Fig. 7b, bottom).^11^ This manifests the ambiphilic character of the boryl ligand and how it can be tuned by modifying the ligand *trans* to it.

**Fig. 8:**
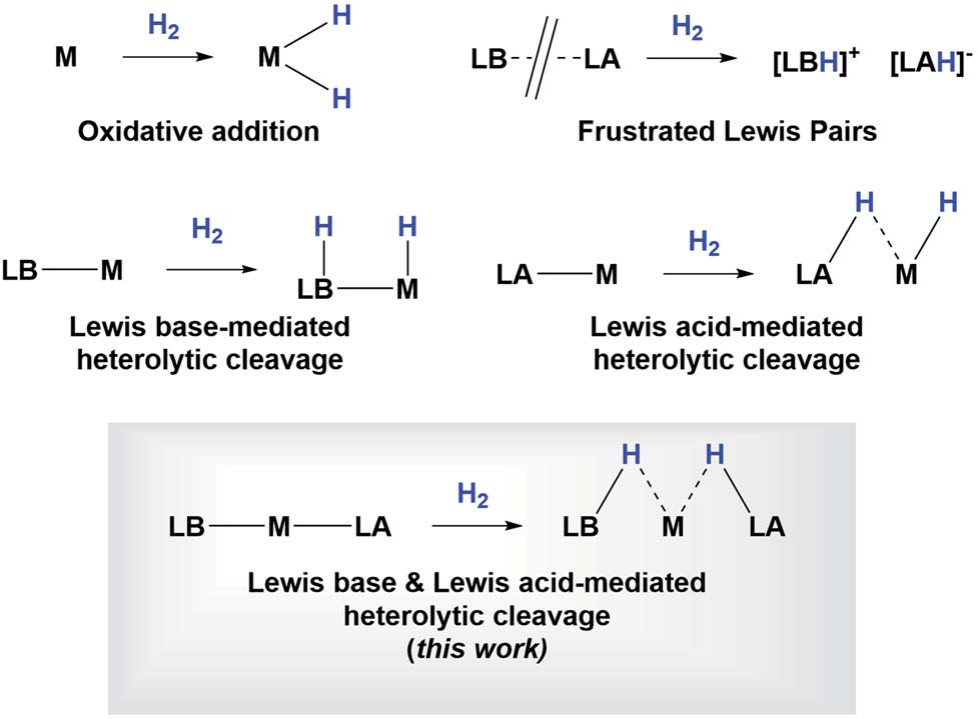
Different types of H_2_ activation.

Studying the centroids in TS2 reveals how nickel assists the transfer of a hydride from the diaminoborane to the boron atom of HBcat, forming the H_2_Bcat^–^ fragment. At the same time, an electron pair of Ni moves away from the metal to forge a Ni–B bond with the boron atom of the PBP ligand, reconstituting the pincer scaffold in a process where the oxidation state of the metal increases from Ni(0) to Ni(ii) (red arrow, Fig. S51†). This mechanism for H_2_ activation is novel compared to preceding reports according to recent classifications (Fig. 8)^7*b*^ due to the presence of two boryl ligands with different Lewis acid/base behaviour. In fact, some previous methodologies for activating dihydrogen with nickel involve cationic species with a vacant site to form the H_2_ complex followed by deprotonation,^24,29^ or the use of electron rich, Ni(0) derivatives able to weaken the H–H bond *via* back-donation to the σ* orbital of H_2_ in combination with Lewis acidic ligands.^9,30^ As a comparison, 3 is a neutral Ni(ii) four-coordinate complex with no vacant position in its coordination sphere, and the aforementioned data suggest that the presence of the two boryl ligands is responsible for the successful H_2_ activation.

**Scheme 5:**
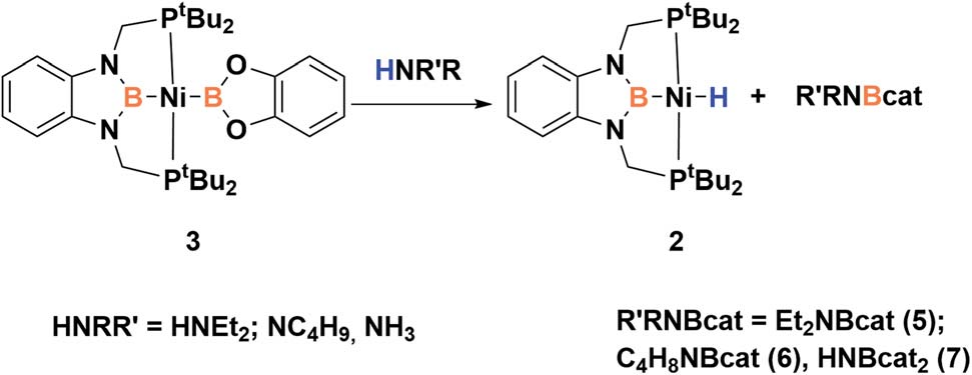
Reactivity of 3 with amines and ammonia.

Given the reactivity observed with 3 towards dihydrogen and the conclusions extracted from DFT calculations we were keen to study the electrophilic behavior of the dioxoboryl group. Thus, we explored the reactivity of 3 towards different nucleophilic species. First, we performed the reaction towards amines in order to learn whether the coordination to the electrophilic boron center takes place (Scheme 5). Thus, treatment of 3 with secondary amines, such as diethylamine and pyrrolidine, allows the instantaneous formation in both cases of a new species that was identified as the nickel hydride complex (2, see Scheme 5), and the corresponding aminoborane products 5 and 6.^31^ These species were characterized by ^1^H, ^11^B and ^13^C NMR spectroscopy and in the case of 6 also by X-ray diffraction analysis. 5 shows only one ^11^B chemical shift at 25.9 ppm in solution, characteristic of a three-coordinated boron atom. In the case of 6, which is a dimer in the solid state, the ^11^B NMR spectrum shows two different resonances, one at 25 ppm and another one at 9 ppm, which is the expected chemical shift for a tetra-coordinated boron, revealing a monomer–dimer equilibrium in solution for this species (see ESI†). Additionally, when 3 is reacted with deuterated diethylamine (Et_2_ND) the formation of the nickel-deuteride (2-D) complex is confirmed by ^2^H NMR spectroscopy (see ESI†).

This reactivity towards amines might be consistent with a highly Lewis acidic boron center at the Bcat moiety that enables the initial coordination of the amine followed by activation of the N–H bond whether by a cooperative mechanism involving the second boron center or by a likely metathesis pathway. Encouraged by these results we sought to explore if the borylation of NH_3_ can also be accomplished by 3. Activation of ammonia is quite challenging due to the tendency of this molecule to form stable Werner-type complexes that do not undergo further N–H activation as a consequence of the high strength of the N–H bond.^32,33^ Different approaches to overcome these difficulties encompass the use of electron rich transition metal complexes to favor N–H cleavage by oxidative addition,^34^ main group systems,^35^ bimetallic species,^36^ transition metal systems with non-innocent ligands that operate cooperatively,^37^ metal coordination to induce N–H bond-weakening^38^ or activation of ammonia through different concerted mechanisms.^39^

The reaction of ammonia with 3 yielded nickel–hydride complex 2,^40^ similarly to that observed with amines, and the double borylation product of ammonia (HNBcat_2_). This species was characterized by comparison of its NMR spectra with those of a sample independently synthesized by dehydrogenative borylation of NH_3_ and HBCat using a platinum catalyst (see ESI† for details).^31^ It is important to mention that no reaction was observed when a solution of either 2 or nickel methyl complex 1 was treated with ammonia (even at 70 °C), highlighting the role of the dioxoboryl group in this process.

Finally, further study of the electrophilic behavior of Bcat on 3 was performed by analysis of its reactivity towards carbon dioxide. Unfortunately, no reaction was observed with CO_2_, even after prolonged heating at 70 °C. This lack of reactivity might be an indication of the insufficient electrophilic character of this boron center to react with the oxygen atoms of CO_2_ but also of the low nucleophilicity of the dioxoboryl moiety that is not basic enough to attack the electrophilic carbon of carbon dioxide.^41^

## Conclusions

In conclusion, we have prepared a bis(phosphino)boryl (PBP) nickel complex that accommodates a second boryl group (Bcat) *trans* to the boron atom of the pincer ligand. This nickel complex is able to reversibly activate dihydrogen in a mechanism that involves the cooperation between the metal and both boryl moieties through a concerted five-center process. We have shown that the electronic behavior of the diaminoboryl group on the PBP ligand can be changed by modifying the ligand *trans* to it (boryl instead of methyl) and we provide valuable information on the key role of the second boryl group in the reactivity of this compound. We have found that after the splitting of the dihydrogen molecule, the boron atom from the PBP ligand behaves as a nucleophile, accepting a formally protic hydrogen, whereas the hydride-like fragment ends up bonded to the catecholboryl moiety that acts as an electrophile. Additionally, the Lewis acidic character of the Bcat group was further corroborated by reactivity towards Lewis bases such as amines and ammonia.

This work discloses an unprecedented mechanism that shows the impact of the boryl–metal–boryl arrangement on the facile and reversible activation of the H–H bond, which could be useful for the design of catalytic systems that may be able to perform other non-polar bond activations.

## Conflicts of interest

There are no conflicts to declare.

## Supplementary Material

SC-012-D0SC06014C-s001

SC-012-D0SC06014C-s002

SC-012-D0SC06014C-s003
