# Supplementary material for: Ambiphilic boryl groups in a neutral Ni(ii) complex: a new activation mode of H2
Source: Chem Sci. 2020 Dec 22;12(7):2540–8. doi: 10.1039/d0sc06014c (PMC8179274; doi:10.1039/d0sc06014c)
Supplement: SC-012-D0SC06014C-s001 [file SC-012-D0SC06014C-s001.pdf]

Electronic Supplementary Material (ESI) for Chemical Science.
This journal is © The Royal Society of Chemistry 2020

Supporting Information

Ambiphilic Boryl Groups in a Neutral Ni(II) Complex: A New Activation Mode of H_2

Pablo Ríos, [a] Javier Borge, [b] Francisco Fernández de Córdova, [a] Giuseppe Sciortino, [c] Agustí Lledós,* [c] Amor Rodríguez* [a]

[a] *Instituto de Investigaciones Químicas-Departamento de Química Inorgánica. Universidad de Sevilla-Consejo Superior de Investigaciones Científicas, Centro de Innovación en Química Avanzada (ORFEO-CINQA). C/ Américo Vespucio 49, 41092 Sevilla, Spain.*

[b] *Departamento de Química Física y Analítica, Centro de Innovación en Química Avanzada (ORFEO-CINQA), Universidad de Oviedo, C/Julián Clavería 8, 33006, Oviedo, Spain.*

[c] *Departament de Química-Centro de Innovación en Química Avanzada (ORFEO-CINCA), Universitat Autònoma de Barcelona. Campus UAB, 08193 Cerdanyola del Vallès, Spain*

To whom correspondence should be addressed: agusti@klingon.uab.es; marodriguez@iiq.csic.es

TABLE OF CONTENTS

I- EXPERIMENTAL PROCEDURES **3**

II- NMR SPECTROSCOPIC DATA..**8**

III- X-RAY STRUCTURAL CHARACTERIZATION...**31**

IV- COMPUTATIONAL DETAILS..**38**

V – COMPARISON OF X-RAY AND OPTIMIZED GEOMETRICAL PARAMETERS OF **3** **39**

VI – GEOMETRICAL PARAMETERS OF INTERMEDIATES AND TRANSITION STATES IN THE ENERGY PROFILE OF FIGURE 5..**40**

VII – NATURAL CHARGE ANALYSIS OF INTERMEDIATES AND TRANSITION STATES IN THE ENERGY PROFILE OF FIGURE 5..**45**

VIII – LOCALIZED MOLECULAR ORBITAL ANALYSIS..**52**

IX – COMPARATION OF THE REACTION WITH PBP AND CARBENE LIGANDS **53**

X- ENERGY PROFILE OF THE REACTION WITH UNCONSTRAINED PBP LIGANDS **55**

XI – SELECTED MOLECULAR ORBITALS OF **3** **56**

XII – QTAIM ANALYSIS OF **4** **58**

XIII – OPTIMIZED GEOMETRIES (ENERGY PROFILE SPECIES) **60**

XIV – CARTESIAN COORDINATES OF THE OPTIMIZED STRUCTURES **63**

XV- REFERENCES **79**

I- EXPERIMENTAL PROCEDURES

General considerations. All manipulations were carried out using standard Schlenk and glove box techniques under an atmosphere of argon and of high purity nitrogen, respectively. All solvents were dried and degassed prior to use. Toluene and *n*-pentane were distilled over sodium. Benzene-d_6 (C_6D_6) was distilled under argon over sodium and CD_2Cl_2 was distilled over CaH_2. Both were then degassed and dried over 4 Å molecular sieves. Ammonia was dried over CaO and then condensed over sodium metal chunks. All other compounds were commercially available and were used as received. Solution NMR spectra were recorded on a Bruker DRX-400 spectrometer and they were referenced to external $SiMe_4$ (δ 0 ppm) using the residual protio solvent peaks as internal standard (^1H NMR experiments) or the characteristic resonances of the solvent nuclei (^{13}C NMR experiments). ^{11}B NMR spectra were referenced to an external standard of $BF_3{\cdot}Et_2O$. ^{31}P NMR chemical shifts were referenced to an external 85% solution of H_3PO_4 in the adequate solvent. Pyrrolidine, diethylamine and ammonia were dried before use. Ligand **PBP** was prepared according to the procedure previously described in the literature. [1] $NiBr_2$(dme) and bis(catecholato)diboron were purchased from Aldrich and used as received. [(^{tBu}PBP)NiBr] and [(^{tBu}PBP)NiMe] (**1**) were prepared as previously described. [2]

Synthesis of [(^{tBu}PBP)NiBCat] (3)

85 mg (0.167 mmol) of [(^{tBu}PBP)NiMe] (**1**) were suspended in 2.5 mL of pentane and then 41.6 mg (0.175 mmol) of B_2Cat_2 were added as a solid. Then, the mixture was stirred at room temperature overnight and the yellow solid that precipitates was washed with pentane (2 x 3 mL). Afterwards the solid was dried in vacuum to afford **3** as a bright yellow solid; yield 72 mg (70%). Yellow crystals suitable for X-ray diffraction analysis were obtained by cooling at -23°C a solution of **3** in toluene/pentane, (1 mL/2 mL).

^1H NMR (400 MHz, C_6D_6, 25 °C): *δ* 1.17 (vt, 36 H, $^3J_{HP}$ = 6 Hz, ^{t}Bu), 3.82 (s, 4 H, CH_2), 6.88 (dd, 2 H, $^3J_{HH}$ = 6, $^4J_{HH}$ = 3.3 Hz, C*H*-aromatic), 7.03 (dd, 2 H, $^3J_{HH}$ = 6, $^4J_{HH}$ = 3.2 Hz, C*H*-aromatic), 7.18 (dd, 2 H, $^3J_{HH}$ = 6, $^4J_{HH}$ = 3 Hz, C*H*), 7.34 (dd, 2 H, $^3J_{HH}$ = 6, $^4J_{HH}$ = 3 Hz, C*H*-aromatic) ppm. $^{13}C\{^1H\}$ NMR (100 MHz, C_6D_6, 25 °C): δ 29.5 (s, *C*H_3- ^{t}Bu), 35.4 (t, $^1J_{PC}$ = 8

Hz, *Cq*-^{t}Bu), 42.8 (t, $^1J_{PC}$ = 15 Hz, *C*H_2), 109.3 (s, *C*H-aromatic), 110.7 (s, *C*H-aromatic), 118.3 (s, *C*H-aromatic), 120.5 (s, *C*H-aromatic), 139.5 (t, $^3J_{PC}$ = 9 Hz, *C*q-aromatic), 150.9 (s, *C*q-aromatic-Bcat). ^{31}P{^{1}H} (161 MHz, C_6D_6, 25 °C) δ 117.3 (s) ppm. ^{11}B{^{1}H} (128 MHz, C_6D_6, 25 °C) δ 59 and 49 (bs) ppm. Elemental Analysis $C_{30}H_{48}B_2N_2NiO_2P_2$ (610.98) Calcd: C 58.98, H 7.92, N 4.59; found: C 59.27; H 7.40, N 4.74.

Synthesis of [(^{tBu}PBP)NiH_2Bcat] (4)

15 mg (0.024 mmol) of **3** were dissolved in 500 μL of C_6D_6. The solution was placed in a J. Young valve NMR tube and degassed via three freeze-pump-thaw cycles. Then, the tube was charged with H_2 (4 bar) and an instantaneous colour change from yellow to pale orange was observed. ^{31}P, ^{11}B, ^{13}C and ^{1}H NMR analysis confirmed the quantitative formation of **4**. All volatiles were removed *in vacuo* to give a yellow solid. Yield 16 mg, 90 %. Single crystals of **4** were grown by cooling a C_6D_6/pentane solution (0.1 mL/1.5 mL) to -23 °C during 1 day. Elemental Analysis $C_{30}H_{50}B_2N_2NiO_2P_2$ (613.00) Calcd: C 58.78, H 8.29, N 4.57; found: C 59.12; H 7.96, N 4.18.

^{1}H NMR (400 MHz, C_6D_6, 25 °C): *δ* 1.27 (vt, 36 H, $^3J_{HP}$ = 6 Hz, ^{t}Bu), 1.49 (s, 2H, *H_2*Bcat) (we observed that this chemical shift varies from 1.49 to 0.7 ppm depending on the reaction conditions), 3.63 (s, 4 H, C*H_2*), 6.81 (dd, 2 H, $^3J_{HH}$ = 6, $^4J_{HH}$ = 3.3 Hz, C*H*-aromatic), 6.94 (dd, 2 H, $^3J_{HH}$ = 6, $^4J_{HH}$ = 3.2 Hz, C*H*-aromatic), 6.98 (dd, 2 H, $^3J_{HH}$ = 6, $^4J_{HH}$ = 3.2 Hz, C*H*-aromatic), 7.14 (dd, 2 H, $^3J_{HH}$ = 6, $^4J_{HH}$ = 3.2 Hz, C*H*-aromatic) ppm. ^{13}C{^{1}H} NMR (100 MHz, C_6D_6, 25 °C): δ 29.3 (s, *C*H_3- ^{t}Bu), 35.2 (t, $^1J_{PC}$ = 7 Hz, *Cq*-^{t}Bu), 42.8 (t, $^1J_{PC}$ = 17 Hz, *C*H_2), 108.4 (s, *C*H-aromatic), 111.3 (s, *C*H-aromatic), 118.5 (s, *C*H-aromatic), 121.4 (s, *C*H-aromatic), 138.8 (t, $^3J_{PC}$ = 8 Hz, *C*q-aromatic), 149.5 (s, *C*q-aromatic-Bcat). ^{31}P{^{1}H} (161 MHz, C_6D_6, 25 °C) δ 107.1 (s) ppm. ^{11}B{^{1}H} (128 MHz, C_6D_6, 25 °C) δ 41 and 18 (bs) ppm.

Synthesis of [(^{tBu}PBP)NiD_2Bcat] (4-D_2)

15 mg (0.024 mmol) of **3** were dissolved in 500 μl of C_6D_6. The solution was placed in a J. Young valve NMR tube and degassed via three freeze-pump-thaw cycles. Then, the tube was charged with D_2 (4 bar) and an instantaneous colour change from yellow to

pale orange was observed. ^{31}P, ^{11}B, ^{1}H and ^{2}H NMR analysis confirmed the quantitative formation of **4-D$_2$**.

^{1}H NMR (400 MHz, C_6D_6, 25 °C): δ 1.27 (vt, 36 H, $^{3}J_{HP}$ = 6 Hz, ^{t}Bu), 3.60 (s, 4 H, C*H*$_2$), 6.81 (m, 2 H, C*H*-aromatic), 6.89 (dd, 2 H, $^{3}J_{HH}$ = 6, $^{4}J_{HH}$ = 3 Hz, C*H*-aromatic), 6.98 (dd, 2 H, $^{3}J_{HH}$ = 6, $^{4}J_{HH}$ = 3.2 Hz, C*H*-aromatic), 7.14 (dd, 2 H, $^{3}J_{HH}$ = 6, $^{4}J_{HH}$ = 3.2 Hz, C*H*-aromatic) ppm. ^{2}H NMR (61 MHz, C_6D_6, 25 °C): 1.49 ppm.

H_2/D_2 Exchange Experiment

In a NMR tube, 5 mg (0.008 mol) of **3** were dissolved in 400 µL of C_6D_6. The NMR tube was evacuated under vacuum and D_2 gas (2 bar) was introduced in the NMR tube, then 5 minutes later, H_2 gas (2 bar) was also introduced in the NMR tube. The ^{1}H NMR spectrum of the reaction mixture showed, after 10 minutes at room temperature, formation of HD gas (δ = 4.42 ppm, $^{1}J_{D\text{-}H}$ = 43 Hz) and the corresponding HD complex **4-D$_1$**. H_2 gas was also present (δ = 4.46 ppm).

Reaction of [(^{tBu}PBP)NiBCat] (3) with pyrrolidine

20 mg (0.032 mmol) of **3** were dissolved in 500 µl of C_6D_6. The solution was transferred to a J. Young valve NMR tube and then 2.7 µL (0.032 mmol) of pyrrolidine were added to this solution. ^{31}P, ^{11}B, ^{13}C and ^{1}H NMR analysis confirmed the quantitative and instantaneous formation of **[(^{tBu}PBP)NiH]** (**2**)[2] and (pyrr-Bcat)$_2$.

NMR data of [(^{tBu}PBP)NiH] (2) (identical to RMN data previously reported by our group) [2] ^{1}H NMR (400 MHz, C_6D_6, 25 °C): δ -1.72 (t, 1 H, $^{2}J_{HP}$ = 33 Hz, Ni-*H*), 1.25 (vt, 36 H, $^{3}J_{HP}$ = 7 Hz, ^{t}Bu), 3.79 (bs, 4 H, C*H*$_2$), 7.02 (dd, 2 H, $^{3}J_{HH}$ = 5, $^{4}J_{HH}$ = 3 Hz, C*H*-aromatic), 7.14 (dd, 2 H, $^{3}J_{HH}$ = 5, $^{4}J_{HH}$ = 3 Hz, C*H*-aromatic) ppm. $^{13}C\{^{1}H\}$ NMR (100 MHz, C_6D_6, 25 °C): δ 29.6 (t, $^{2}J_{PC}$ = 3 Hz, *C*H$_3$-^{t}Bu), 34.3 (t, $^{1}J_{PC}$ = 6 Hz, *Cq*-^{t}Bu), 41.7 (t, $^{1}J_{PC}$ = 16 Hz, *C*H$_2$), 108.9 (*C*H-aromatic), 118.0 (*C*H-aromatic), 139.9 (t, $^{3}J_{PC}$ = 8 Hz, *C*q-aromatic). $^{31}P\{^{1}H\}$ (160 MHz, C_6D_6, 25 °C) δ 122.5 (s) ppm. $^{11}B\{^{1}H\}$ (128 MHz, C_6D_6, 25 °C) δ 48 (bs) ppm.

NMR data of pyrrolidine-Bcat: ^{1}H NMR (400 MHz, C_6D_6, 25 °C): δ 1.34 (broad s, 4 H, C*H*$_2$-pyrrolidine), 3.12 (broad s, 4 H, C*H*$_2$-pyrrolidine), 6.78 (m, 2H, C*H*-aromatic), 7.02 (m, 2 H, C*H-aromatic, overlapped with Bcat signals*). $^{13}C\{^{1}H\}$ NMR (100 MHz, C_6D_6, 25

°C): δ 24.1 (s, *C*H_2-pyrrolidine), 43.8 (s, *C*H_2-pyrrolidine), 107.2 (s, *C*H-aromatic), 119.9 (s, *C*H-aromatic), 147.6 (s, *C*q-aromatic). $^{11}B\{^1H\}$ (128 MHz, C_6D_6, 25 °C) δ 23 ppm.

Reaction of [(^{tBu}PBP)NiBCat] (3) with Et_2NH

10 mg (0.016 mmol) of **3** were dissolved in 0.5 mL of C_6D_6 in a J. Young NMR tube and 2 μL (0.020 mmol) of Et_2NH were added via micro syringe. The reaction was monitored by NMR spectroscopy. The ^{31}P, ^{1}H, ^{13}C and ^{11}B NMR spectra contained resonances due to [(^{tBu}PBP)NiH] (**2**) and Et_2N-Bcat after 3 hours at room temperature.

NMR data for Et_2N-Bcat [3a] ^1H NMR (400 MHz, C_6D_6, 25 °C): *δ* 0.92 (t, $^3J_{HH}$ = 7 Hz, 6H, CH_3), 2.98 (q, $^3J_{HH}$ = 7 Hz, 4 H, CH_2), 6.76 (dd, 2 H, $^3J_{HH}$ = 6, $^4J_{HH}$ = 3.5 Hz, *CH-aromatic-Bcat*), 6.98 (dd, 2 H, $^3J_{HH}$ = 6, $^4J_{HH}$ = 3 Hz, *CH-aromatic-Bcat*). $^{13}C\{^1H\}$ NMR (100 MHz, C_6D_6, 25 °C): δ 14.9 (s, CH_3-NEt_2), 39.9 (s, CH_2-NEt_2), 111.3 (s, *CH-aromatic*), 121.5 (s, *C*H-aromatic), 149.4 (s, *C*q-aromatic). $^{11}B\{^1H\}$ (128 MHz, C_6D_6, 25 °C) δ 26 ppm.

Reaction of [(^{tBu}PBP)NiBCat] (3) with Et_2ND

5 mg (0.009 mmol) of **3** were dissolved in 0.5 mL of C_6D_6 in a J. Young NMR tube and 1 uL (0.009 mmol) of Et_2ND [3b] was added via micro syringe. The reaction was monitored by NMR spectroscopy. The ^{31}P, ^{1}H, ^{13}C and ^{11}B NMR spectra contained resonances due to [(^{tBu}PBP)NiD] (**2-D**) and Et_2N-Bcat after 3 hours at room temperature. ^2H NMR (61 MHz, C_6D_6, 25 °C): *δ* -1.71 ppm (t, 1 H, $^2J_{DP}$ = 5 Hz, Ni-*D*).

Reaction of [(^{tBu}PBP)NiBCat] (3) with NH_3

20 mg (0.032 mmol) of **3** were dissolved in 500 μL of C_6D_6. The solution was transferred to a J. Young valve NMR tube and then ammonia (1 bar) was added to this solution. ^{31}P, ^{11}B, ^{13}C and ^{1}H NMR analysis confirmed the quantitative and instantaneous formation of **[(^{tBu}PBP)NiH]** (**2**) [2] and $HN(Bcat)_2$. It is worth mentioning that when the same reaction was performed using not dry NH_3, complex **[(^{tBu}PBP)Ni(NH_3)][$BCat_2$]** (**9**) was also obtained as the major product.

NMR data for HN-$(Bcat)_2$: ^1H NMR (400 MHz, C_6D_6, 25 °C): *δ* 3.54 (s_{broad}, 1H, N-H), 6.73 (dd, 2 H, $^3J_{HH}$ = 6, $^4J_{HH}$ = 3.5 Hz, *CH-aromatic-Bcat*), 6.98 (dd, 2 H, $^3J_{HH}$ = 6, $^4J_{HH}$ = 3 Hz,

CH-aromatic-Bcat). $^{13}C\{^1H\}$ NMR (100 MHz, C_6D_6, 25 °C): δ 111.9 (s, *C*H-aromatic), 122.2 (s, *C*H-aromatic); $^{11}B\{^1H\}$ (128 MHz, C_6D_6, 25 °C) δ 27 ppm.

NMR data for **[(^{tBu}PBP)Ni(NH_3)][$BCat_2$] (9)**: ^1H NMR (400 MHz, CD_2Cl_2, 25 °C): *δ* 1.41 (vt, 36 H, $^3J_{HP}$ = 7 Hz, *t*Bu), 3.95 (s, 4 H, *C*H_2), 6.71 (s_w, 8 H, *CH-aromatic-Bcat*), 7.05 (s_w, 4 H, *CH-aromatic-Bcat*). $^{13}C\{^1H\}$ NMR (100 MHz, CD_2Cl_2, 25 °C): δ 26.6 (S, *C*H_3- ^{t}Bu), 35.5 (t, $^1J_{PC}$ = 6 Hz, *Cq*-^{t}Bu), 39.9 (t, $^1J_{PC}$ = 20 Hz, *C*H_2), 108.5 (*C*H-aromatic), 108.8 (*C*H-aromatic), 118.1 (*C*H-aromatic), 119.1 (*C*H-aromatic), 138.2 (t, $^3J_{PC}$ = 6 Hz, *C*q-aromatic), 151.8 (s, *C*q-aromatic); $^{11}B\{^1H\}$ (128 MHz, CD_2Cl_2, 25 °C) δ 14, 36 ppm.

Alternative synthesis of R_2N-Bcat species using a platinum catalyst [Pt($SiPh_3$)(I^{t}Bu^{i}Pr)$_2$][BAr^F] (8)[4]

In a glovebox, a J. Young valve NMR tube was charged with HBcat (19 μL, 0.166 mmol), the corresponding amine (pyrrolidine (13.7 μL, 0.166 mmol) or Et_2NH (19 μl, 0.166 mmol)) or NH_3 (2 bar) and a solution of **8** (3.5 mg, 2.12 μmol) in CD_2Cl_2 (600 μL) by means of micro syringes. Evolution of H_2 gas was immediately observed and, after 30 minutes at room temperature, the ^1H and ^{11}B NMR spectra of the mixture showed that the reaction was complete. Then, the volatiles were removed in vacuum. The resulting residue was:

For **5**: dissolved in pentane (2 mL) and the solution was filtered off and dried in vacuum to yield a colorless oil that was distilled to yield 7.7 mg (25 %) of **5**. (72 °C /0.1 mmHg). NMR data were identical to that obtained previously by reaction of [(^{tBu}PBP)NiBCat] (**3**) with diethylamine. See Figures S24-28. Elemental Analysis: (**5**) $C_{10}H_{12}BNO_2$ (189.10) Calcd: C 63.54, H 6.40, N 7.41; found: C 63.53; H 6.27, N 7.23.

For **6**: dissolved in a mixture of CH_2Cl_2: pentane (0.5 mL: 2mL) and allowed to stand at –20 °C for 24 h, affording **6** as colorless crystals (9.5 mg, 30 %). NMR data for **6** were identical to that obtained previously by reaction of [(^{tBu}PBP)NiBCat] (**3**) with pyrrolidine. See Figures S15-19. **6** was also characterized by X-ray diffraction analysis (Figure S47). Compound **6,** which is dimeric in the solid state, in solution exhibits two $^{11}B\{^1H\}$ NMR resonances at 24 and 8 ppm. This implies a monomer-dimer equilibrium that favors a

monomer in solution. (**6**) $C_{10}H_{12}BNO_2$ (191.03) Calcd: C 62.87, H 7.39, N 7.33; found: C 62.81; H 7.35, N 7.34.

For **7**: washed with pentane (3 x 2 mL) and dried in vacuum to yield a white solid (10 mg, 24 %). NMR data for **7** were identical to that obtained previously by reaction of [(^{tBu}PBP)NiBCat] (**3**) with NH_3. See Figures S37-40. Elemental Analysis: (**7**) four different samples were analysed and in all cases a satisfactory analysis was only found when coordinated ammonia and water molecules are considered, according to the molecular formula $C_{10}H_9B_2NO_4{\cdot}0.25\ NH_3{\cdot}0.75\ H_20$ (189.10) Calcd: C 53.26, H 4.19, N 6.47; found: C 53.32; H 4.27, N 6.53.

II- NMR SPECTROSCOPIC DATA

All NMR spectra but those corresponding to complex 9, were recorded using C_6D_6 as solvent.

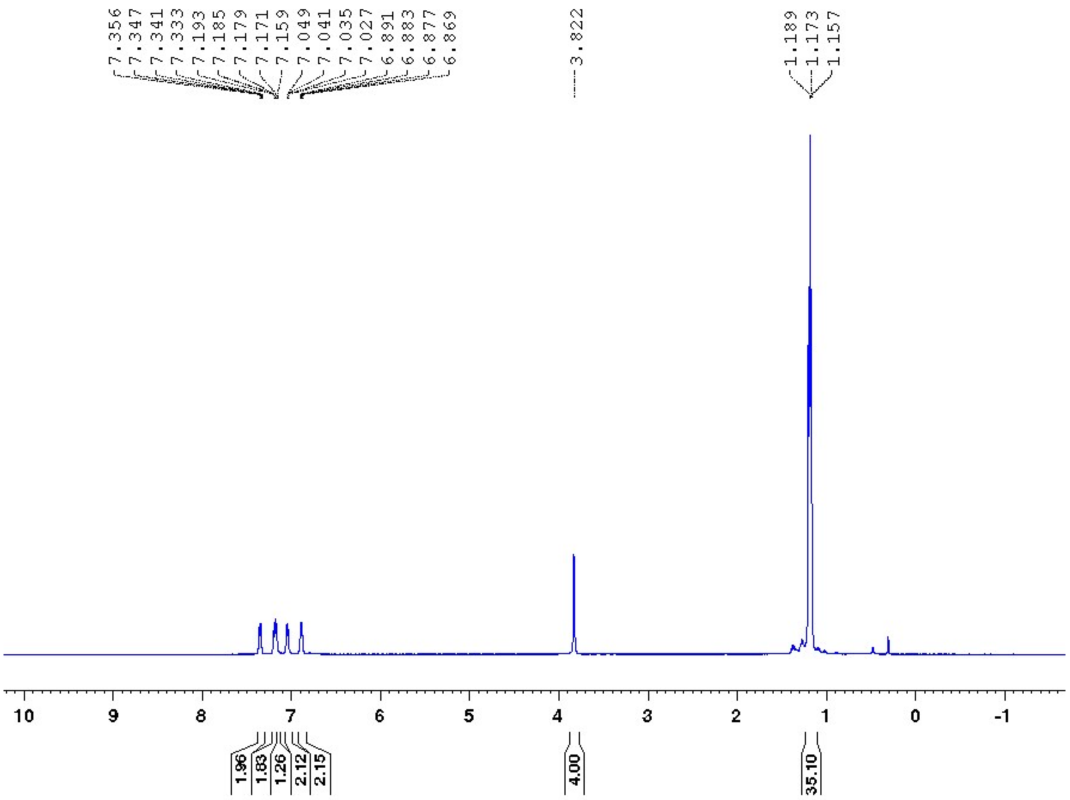

Figure S1: ^{1}H NMR spectrum for complex 3

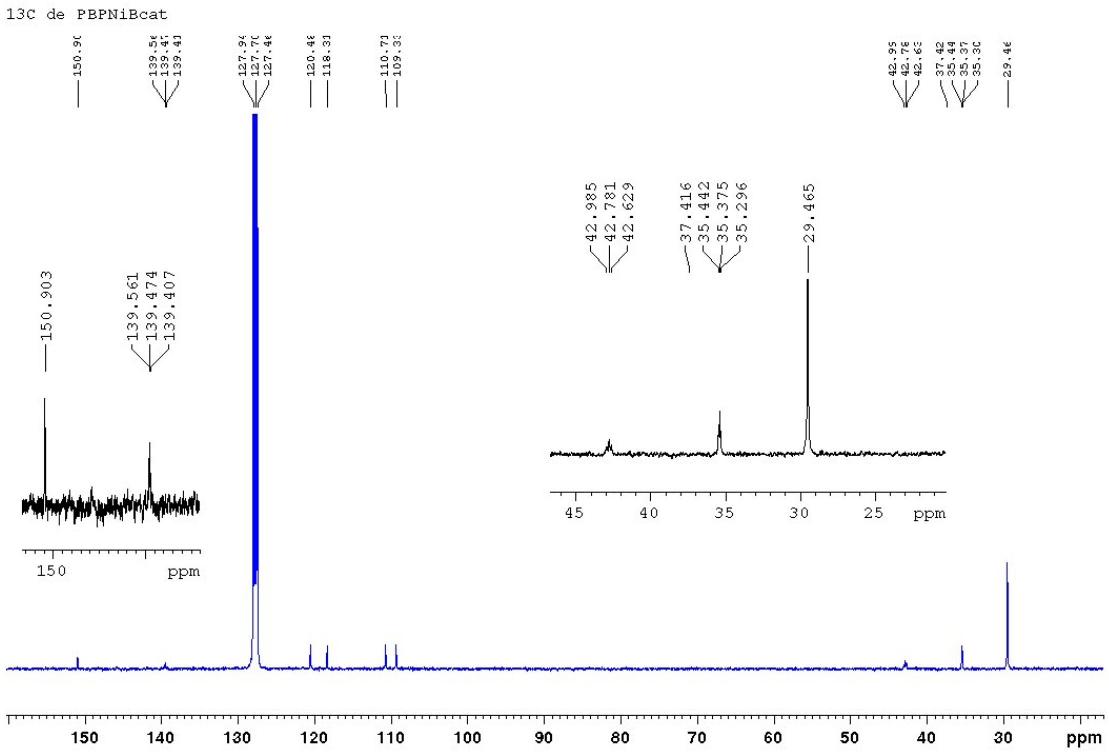

Figure S2: $^{13}C\{^1H\}$ NMR spectrum for complex 3

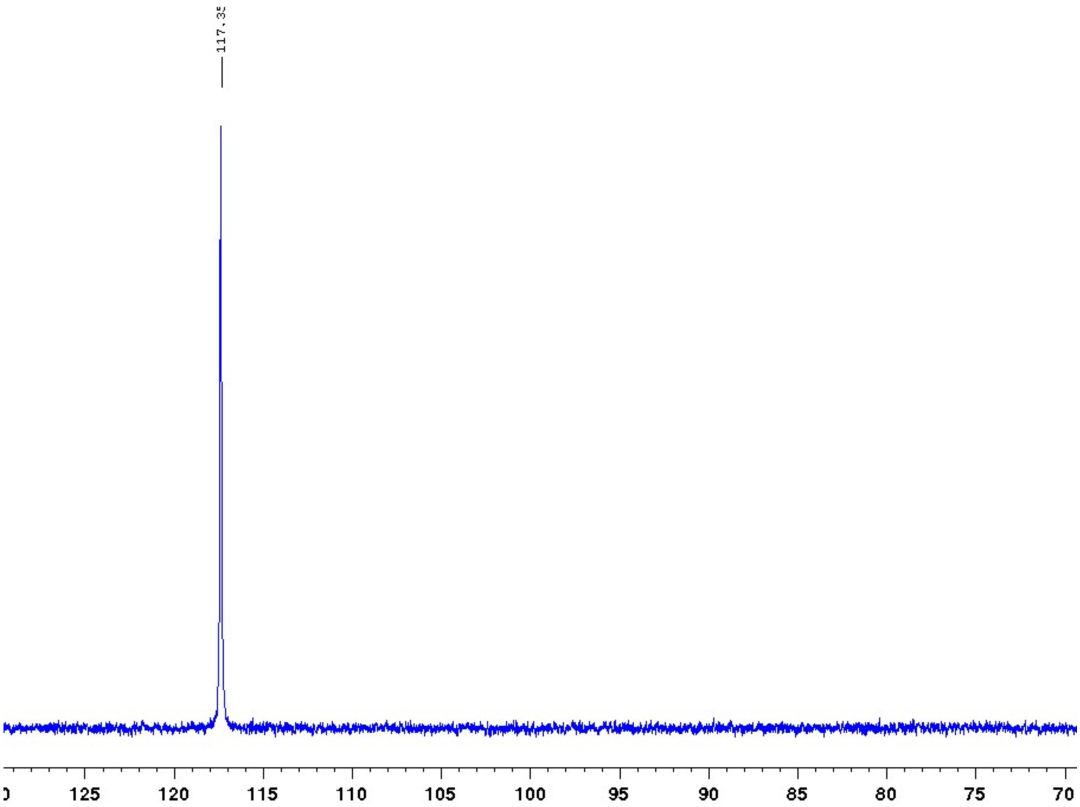

Figure S3: $^{31}P\{^1H\}$ NMR spectrum for complex 3

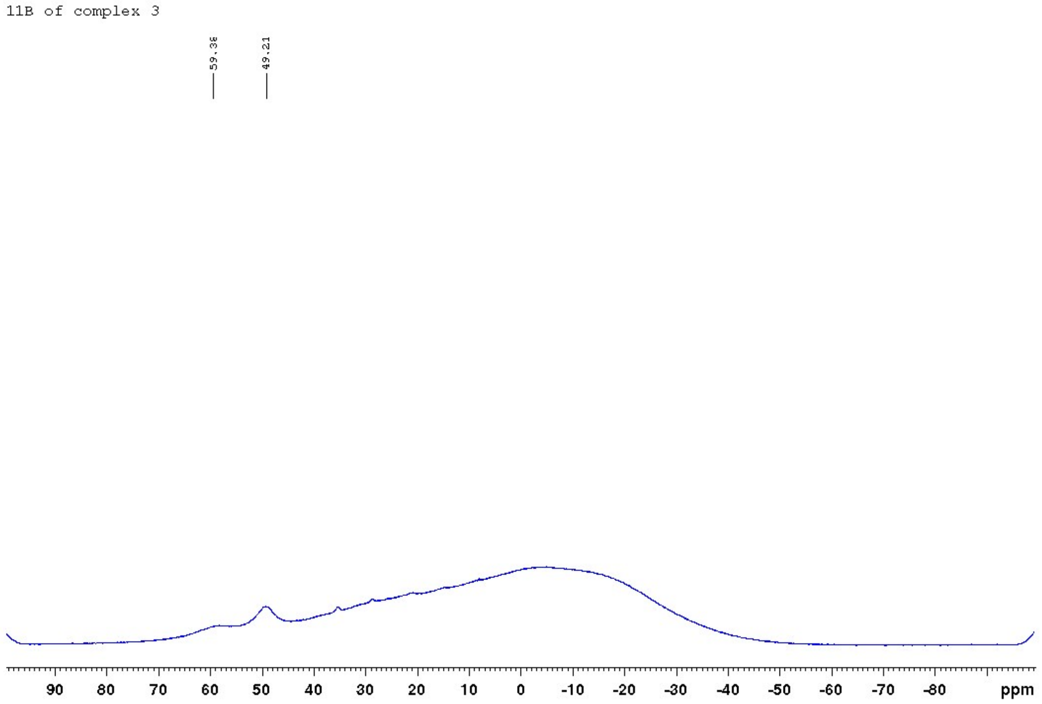

Figure S4: $^{11}B\{^{1}H\}$ NMR spectrum for complex 3

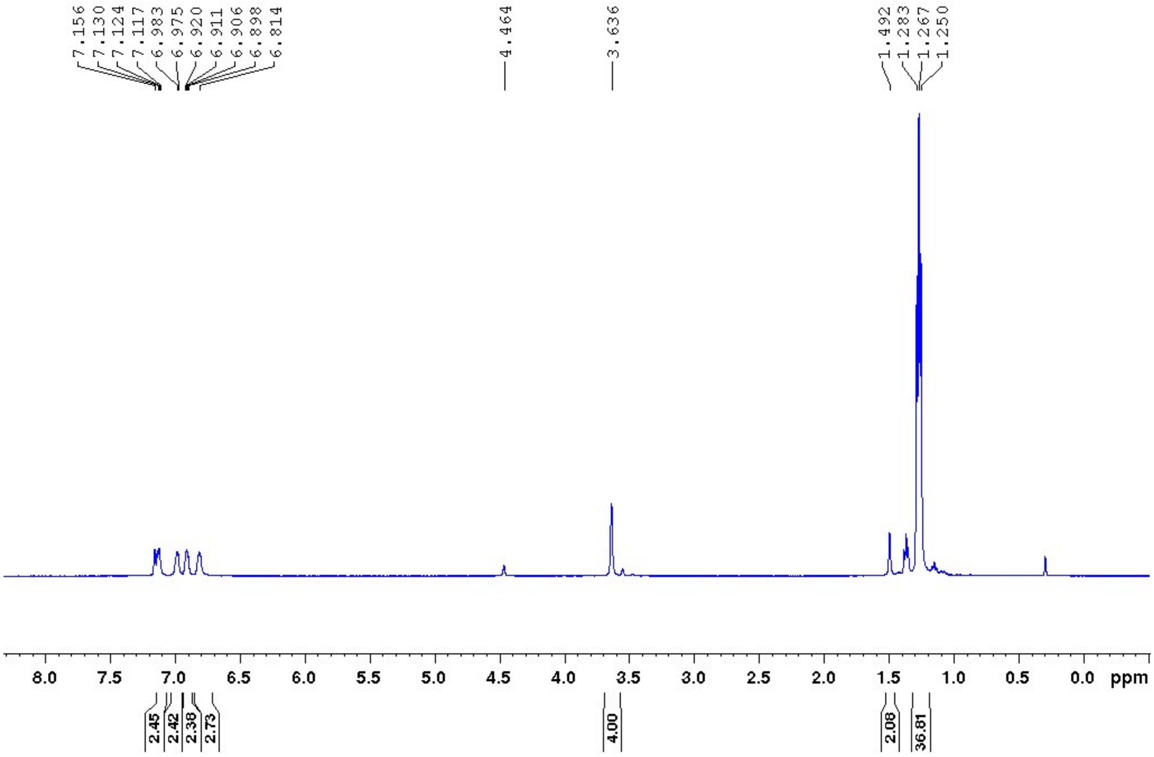

Figure S5: ^{1}H NMR spectrum for complex 4; singlet at 4.46 corresponds to free H_2; small signal at 1.28 ppm corresponds to some decomposition due to traces of water.

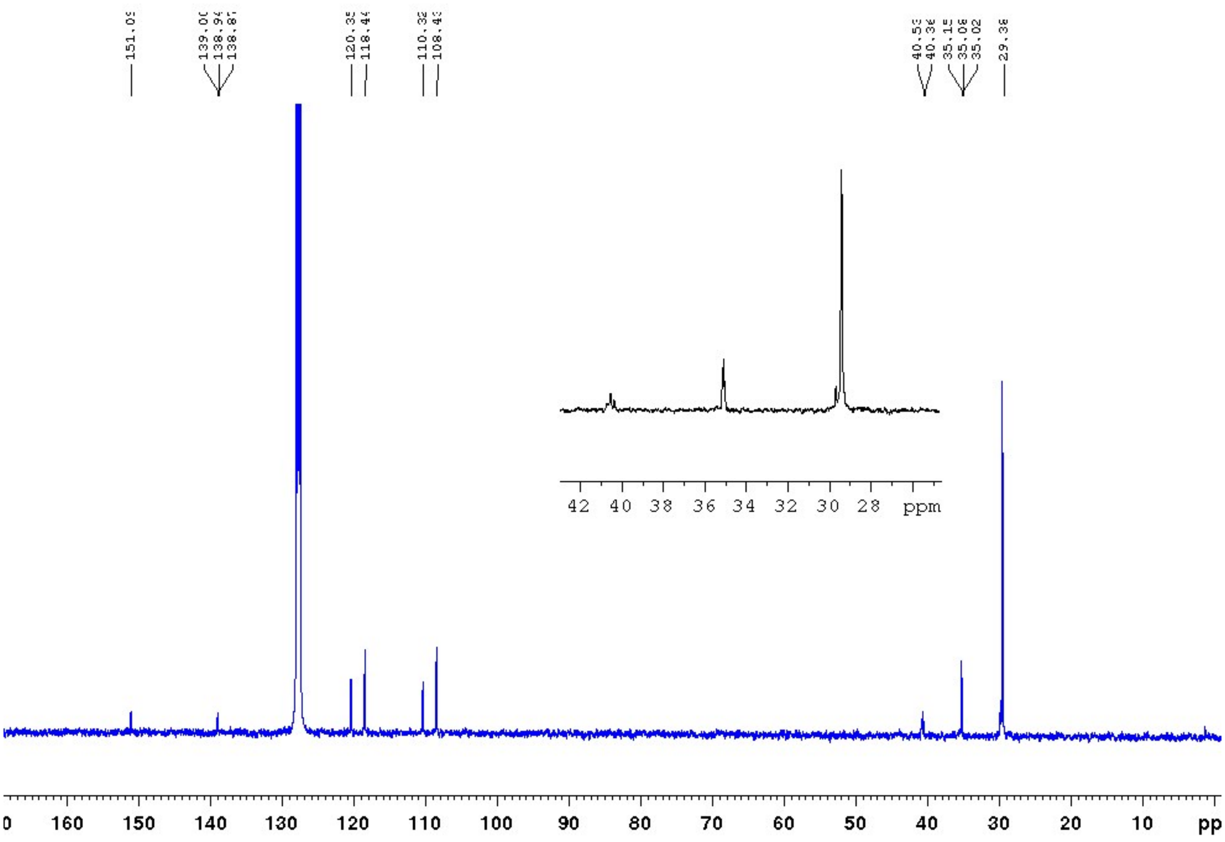

Figure S6: $^{13}C\{^1H\}$ NMR spectrum for complex 4

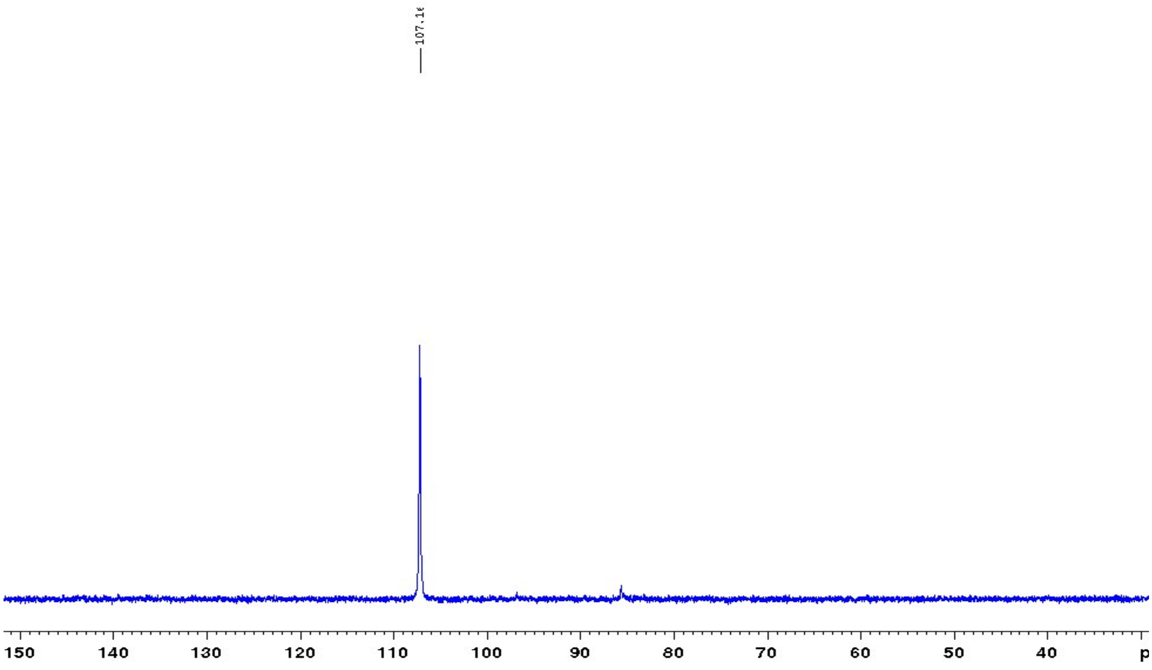

Figure S7: ^{31}P NMR spectrum for complex 4

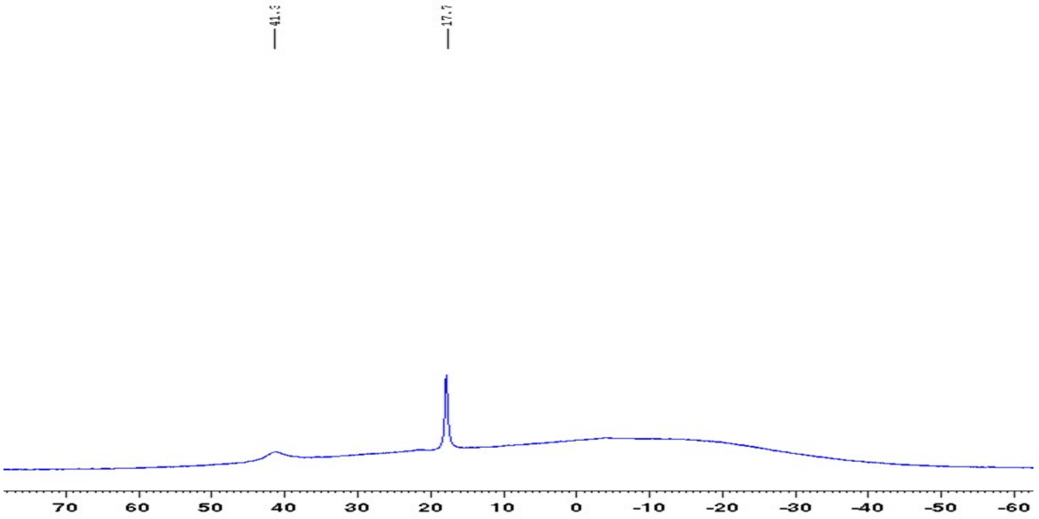

Figure S8: ^{11}B NMR spectrum for complex 4

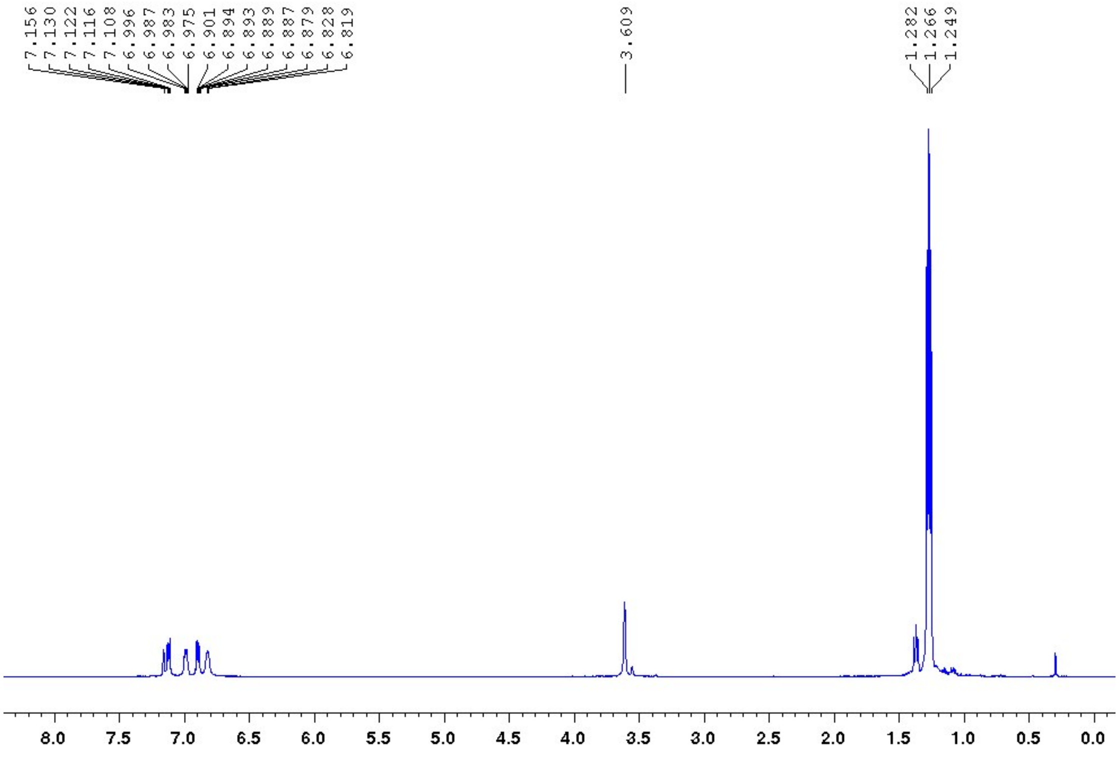

Figure S9: ^{1}H NMR spectrum for complex 4-D_2

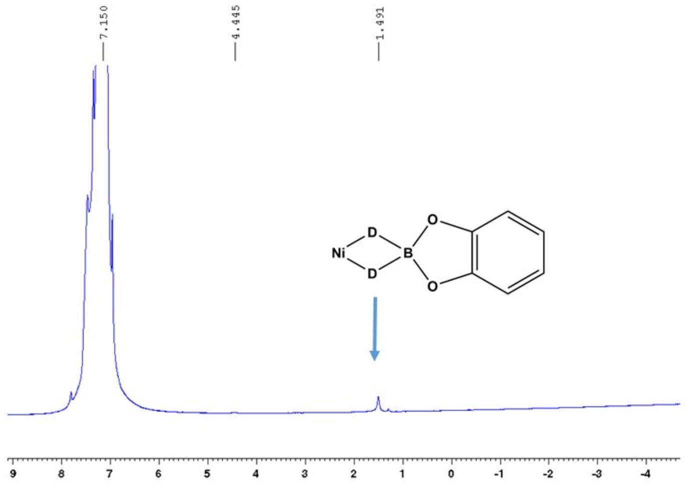

Figure S10: ^{2}H NMR spectrum for complex 4-D_2

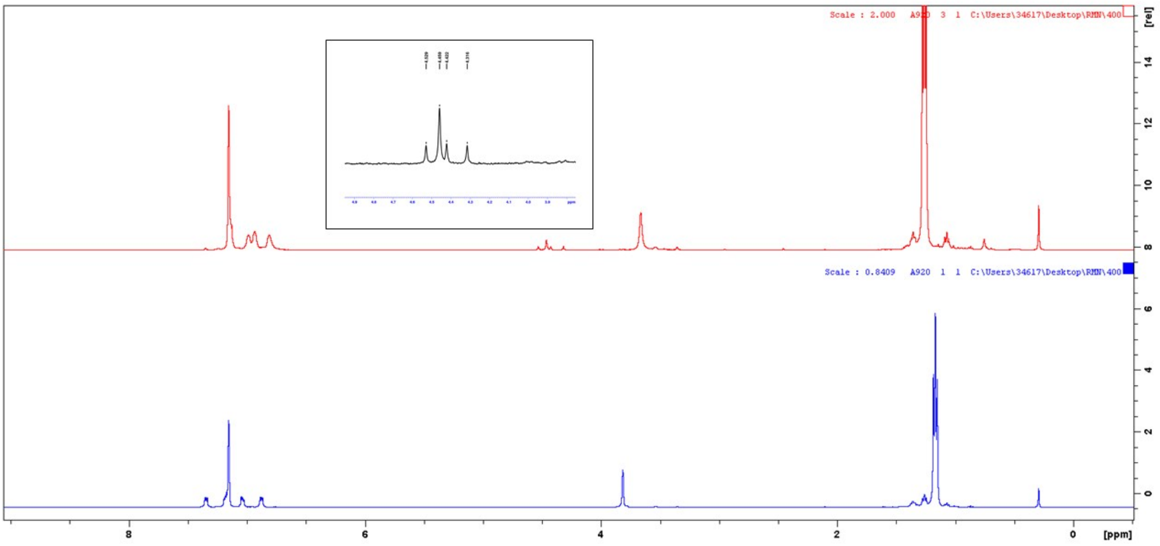

Figure S11: H_2/D_2 Exchange experiment. Bottom (blue): ^{1}H NMR spectrum of 3. Top (red): ^{1}H NMR spectrum of 3 with D_2/H_2; Inset: triplet at 4.42 ppm (J_{DH} = 43.2 Hz) corresponds to HD and singlet at 4.46 ppm corresponds to H_2.

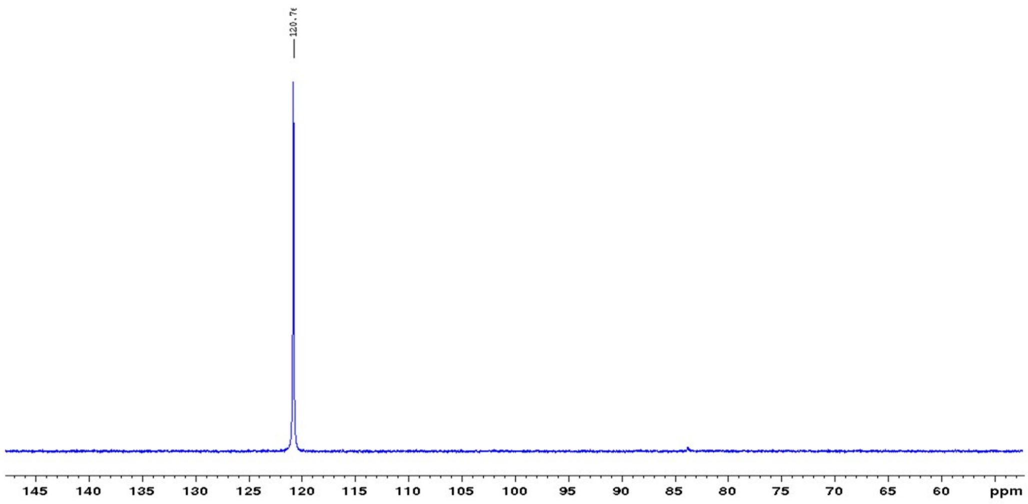

Figure S12: ^{31}P NMR spectrum of the reaction of 1 with pyrrolidine

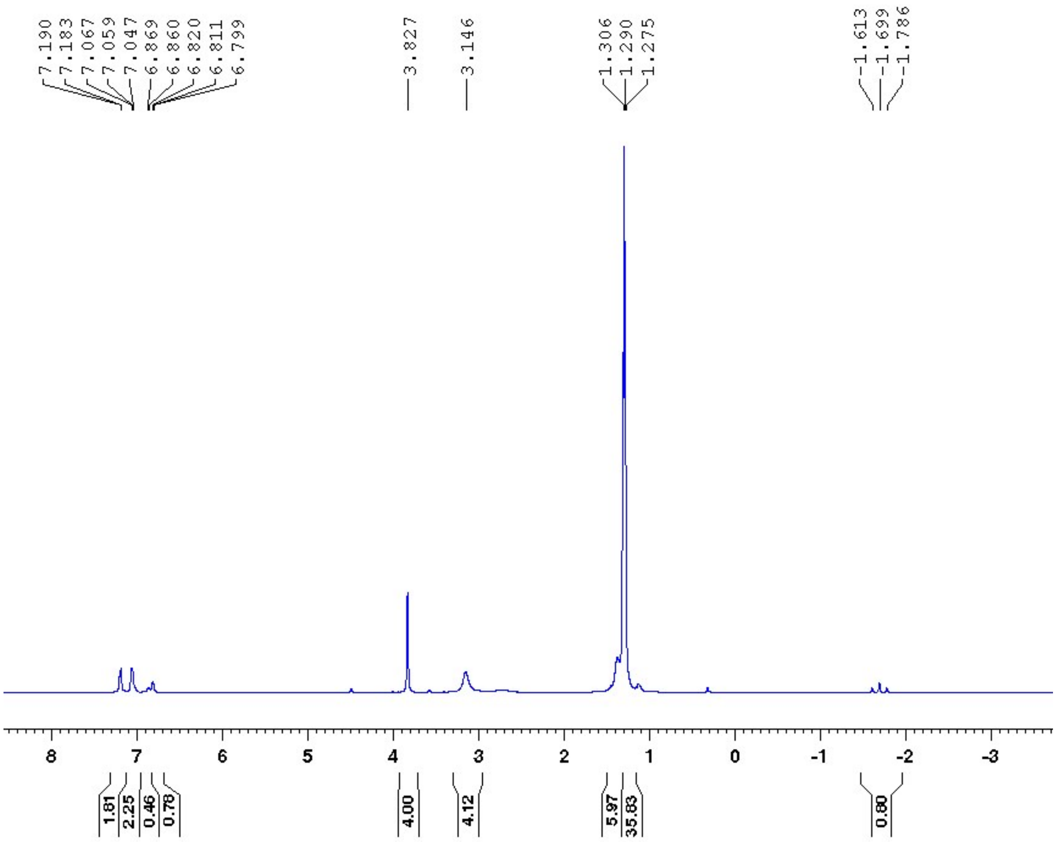

Figure S13: ^{1}H NMR spectrum of the reaction of 1 with pyrrolidine

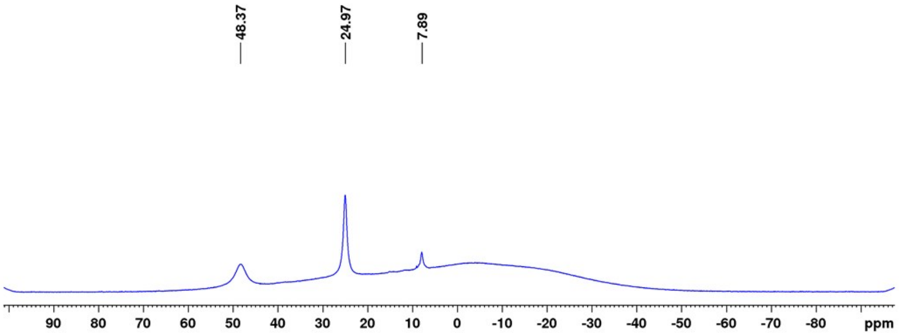

Figure S14: ^{11}B NMR spectrum of the reaction of 1 with pyrrolidine

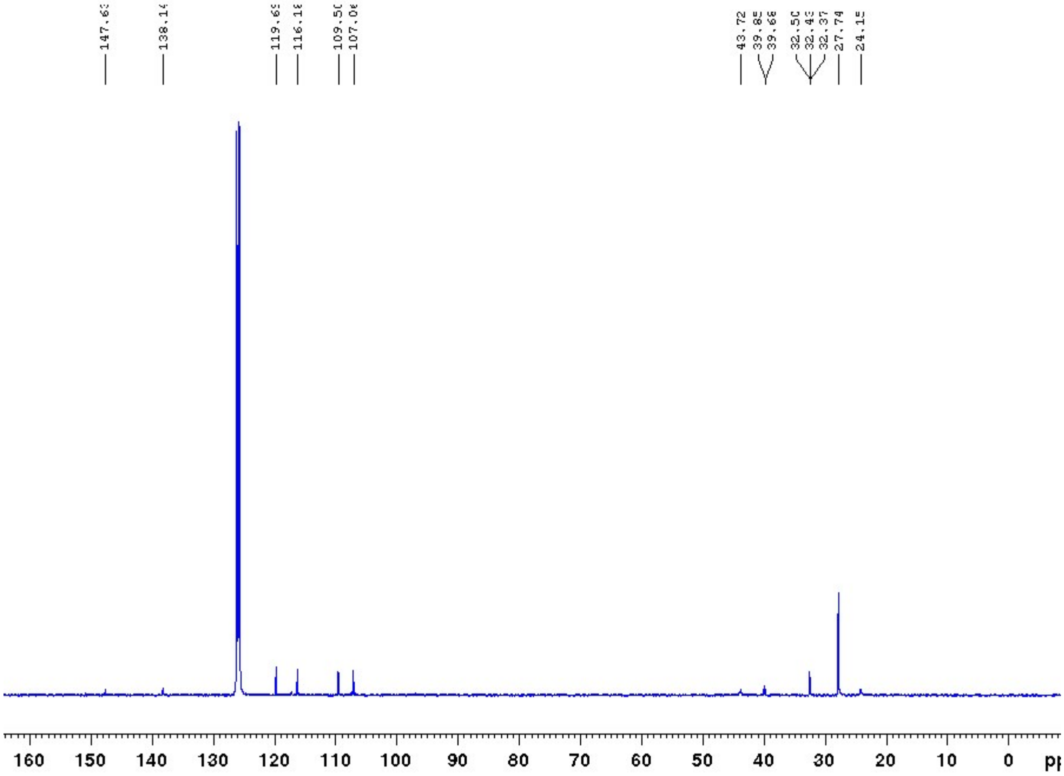

Figure S15: ^{13}C NMR spectrum of the reaction of 1 with pyrrolidine

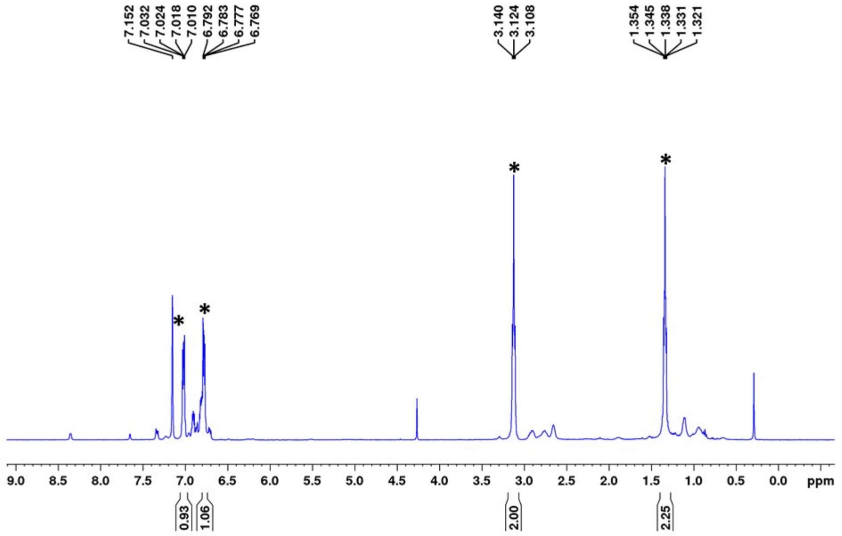

Figure S16: ^{1}H NMR spectrum of the reaction of HBcat with pyrrolidine catalyzed by complex 8. * symbol stands for resonances corresponding to C_4H_4NBcat (major product). The dimer $(C_4H_4NBcat)_2$ is formed as a minor product and was identified by means of ^{11}B NMR and X-ray diffraction analysis (see below).

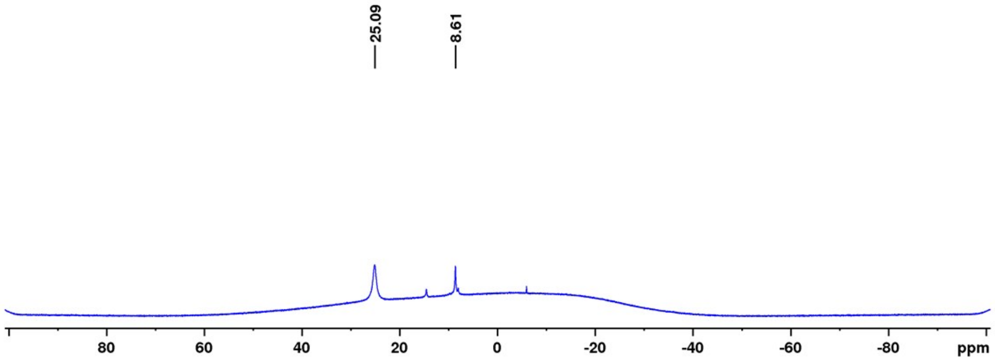

Figure S17: ^{11}B NMR spectrum of the reaction of HBcat with pyrrolidine catalysed by complex 8. Signals at 8.6 ppm and 25.1 ppm correspond to dimer (pyrr-Bcat)$_2$ (minor product) and pyrr-Bcat (major product), respectively.

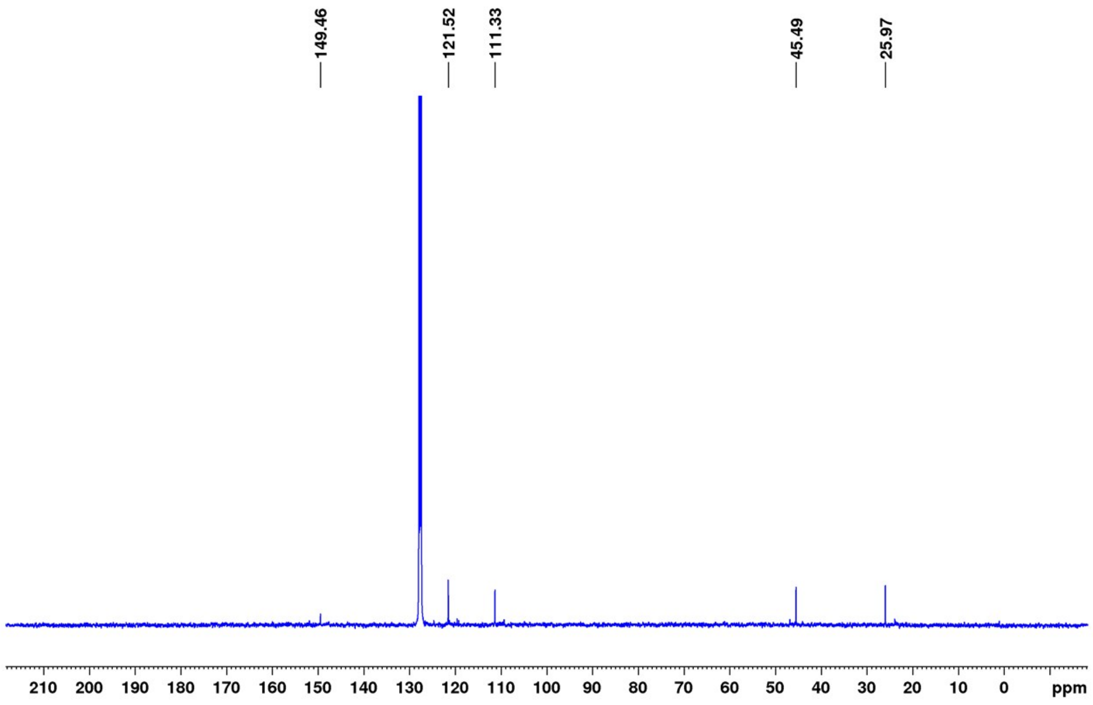

Figure S18: ^{13}C NMR spectrum of the reaction of HBcat with pyrrolidine catalyzed by complex 8.

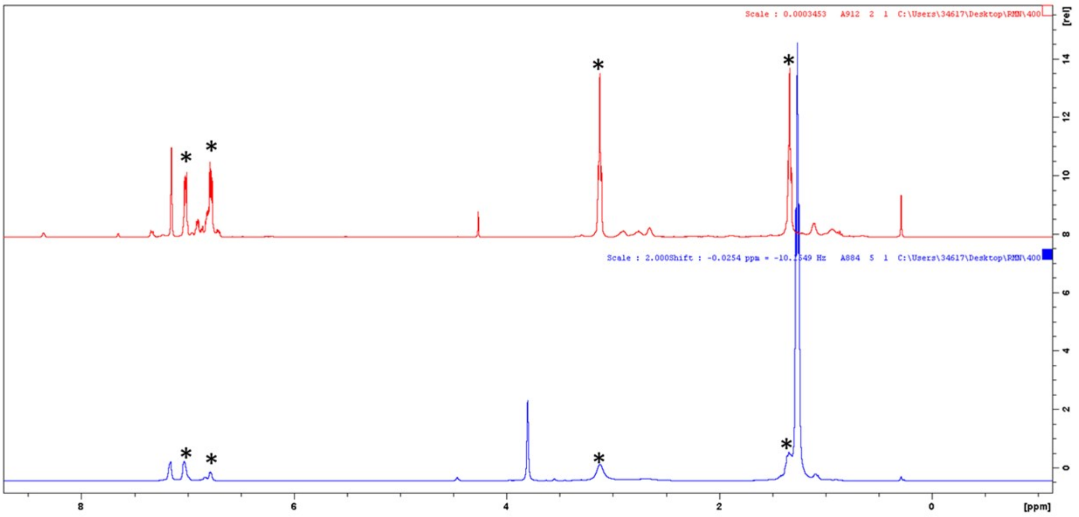

Figure S19: Top (red): ^{1}H NMR spectrum of C_4H_4NBcat generated by catalytic dehydrocoupling of HBcat and pyrrolidine; Bottom (blue): ^{1}H NMR spectrum of the reaction of 1 with pyrrolidine. (* denotes the signals corresponding to C_4H_4NBcat)

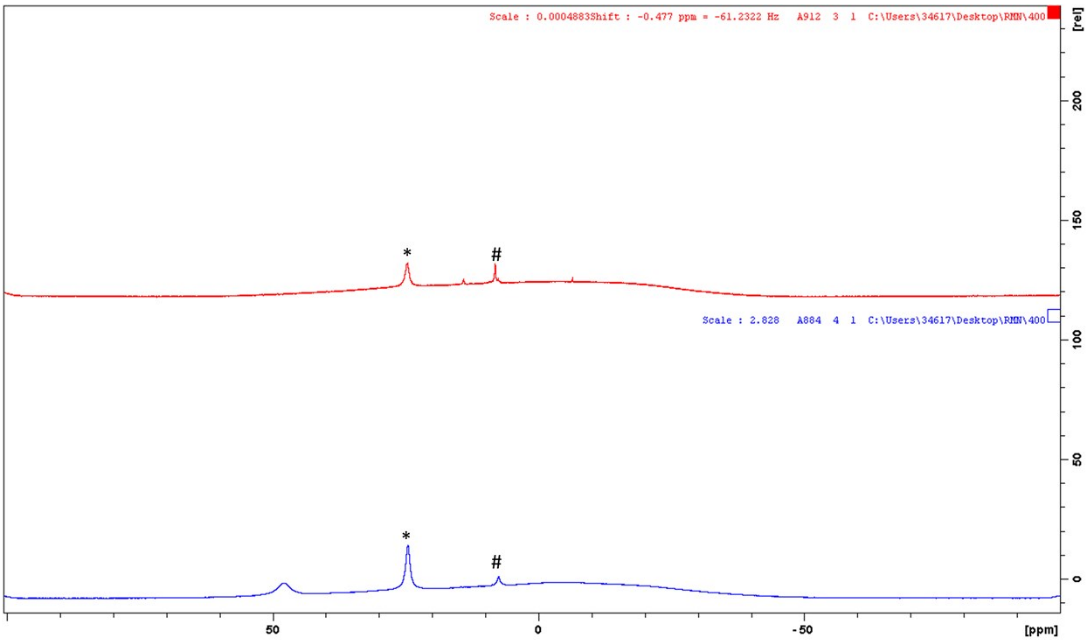

Figure S20: Top (red): ^{11}B NMR spectrum of C_4H_4NBcat generated by catalytic dehydrocoupling of HBcat and pyrrolidine. * symbol stands for resonance corresponding to C_4H_4NBcat (major product); # symbol stands for resonance corresponding to $(C_4H_4NBcat)_2$ which is formed as a minor product. Bottom (blue): ^{11}B NMR spectrum of the reaction of 1 with pyrrolidine.

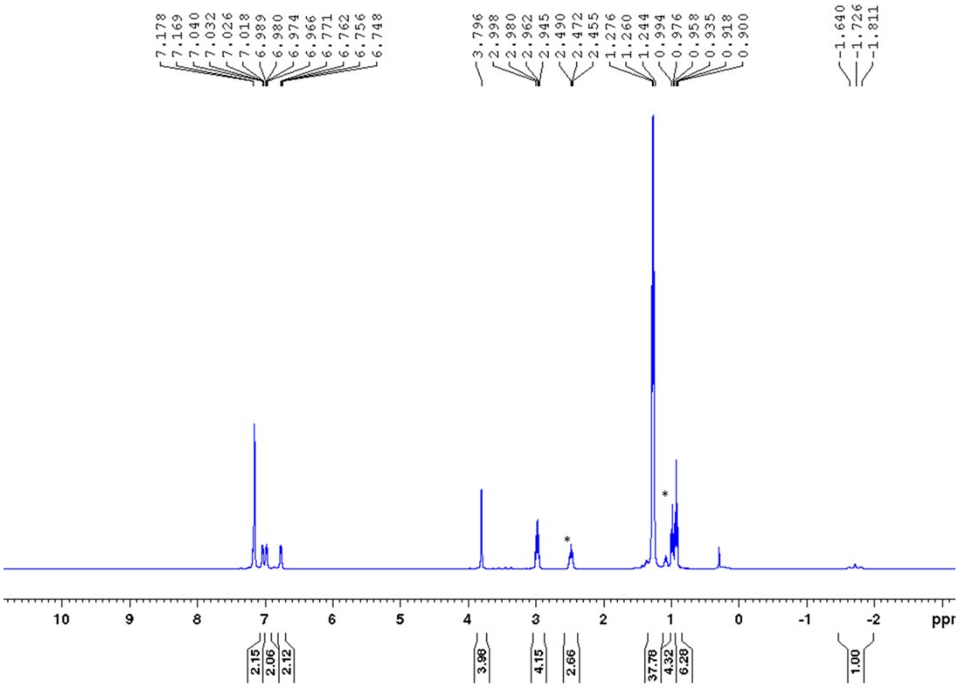

Figure S21: ^1H NMR spectrum of the reaction of 1 with diethylamine. * free $HNEt_2$

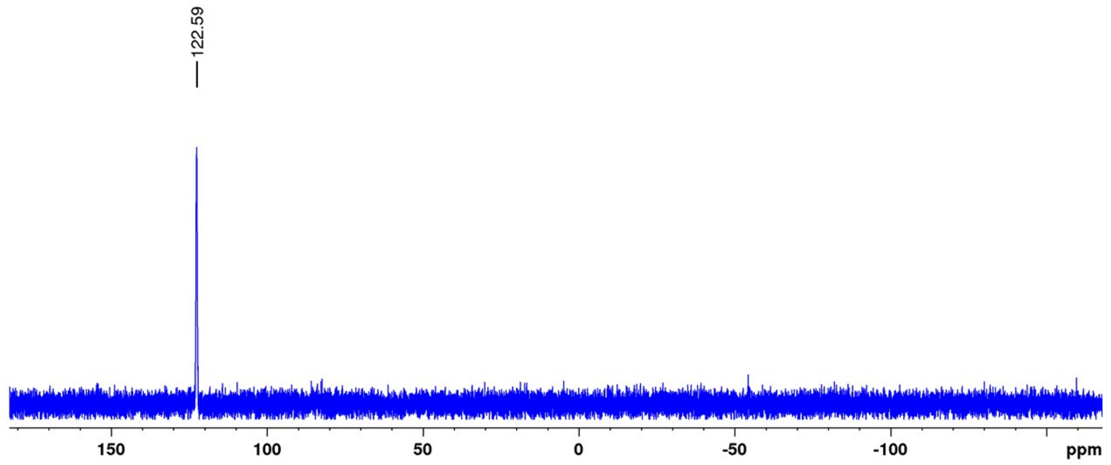

Figure S22: ^{31}P NMR spectrum of the reaction of 1 with diethylamine

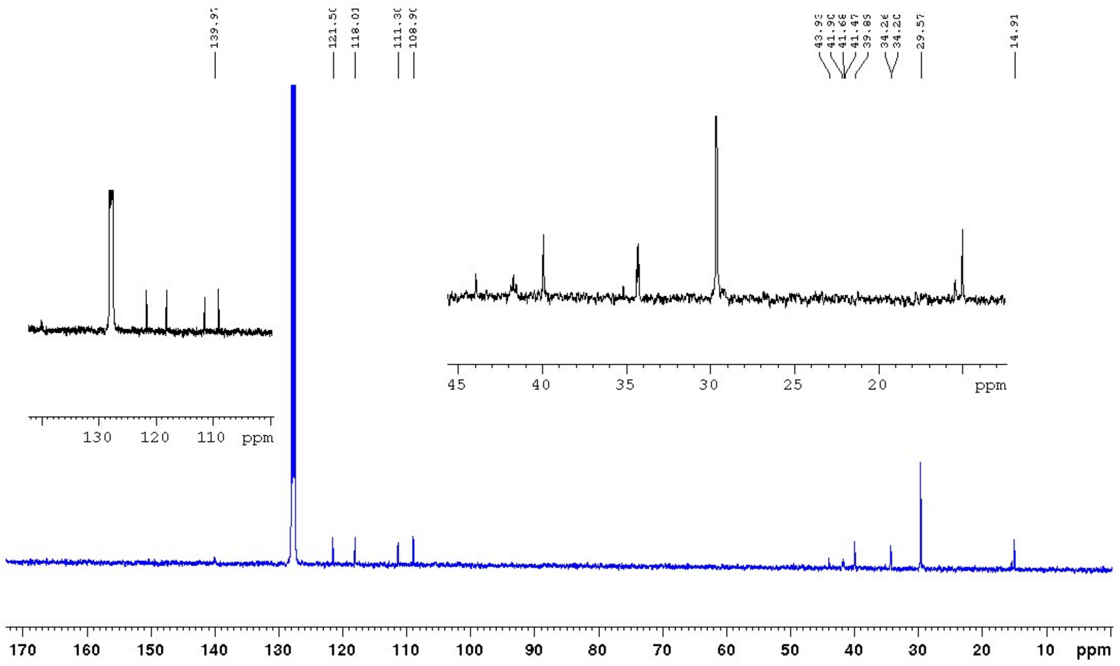

Figure S23: ^{13}C NMR spectrum of the reaction of 1 with diethylamine

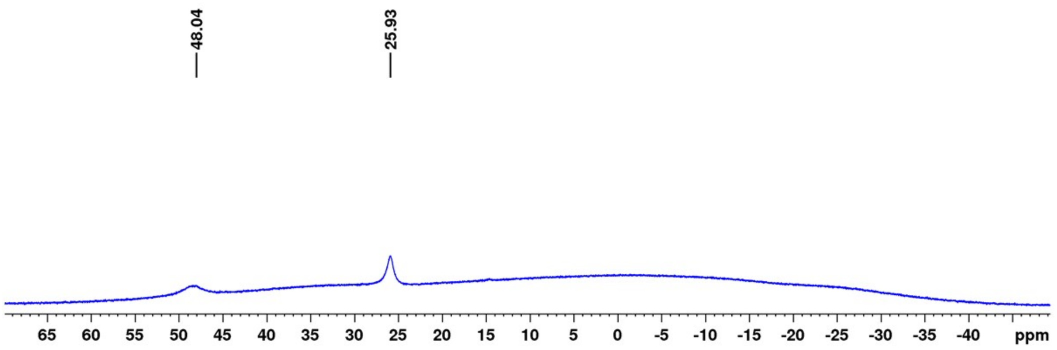

Figure S24: ^{11}B NMR spectrum of the reaction of 1 with diethylamine

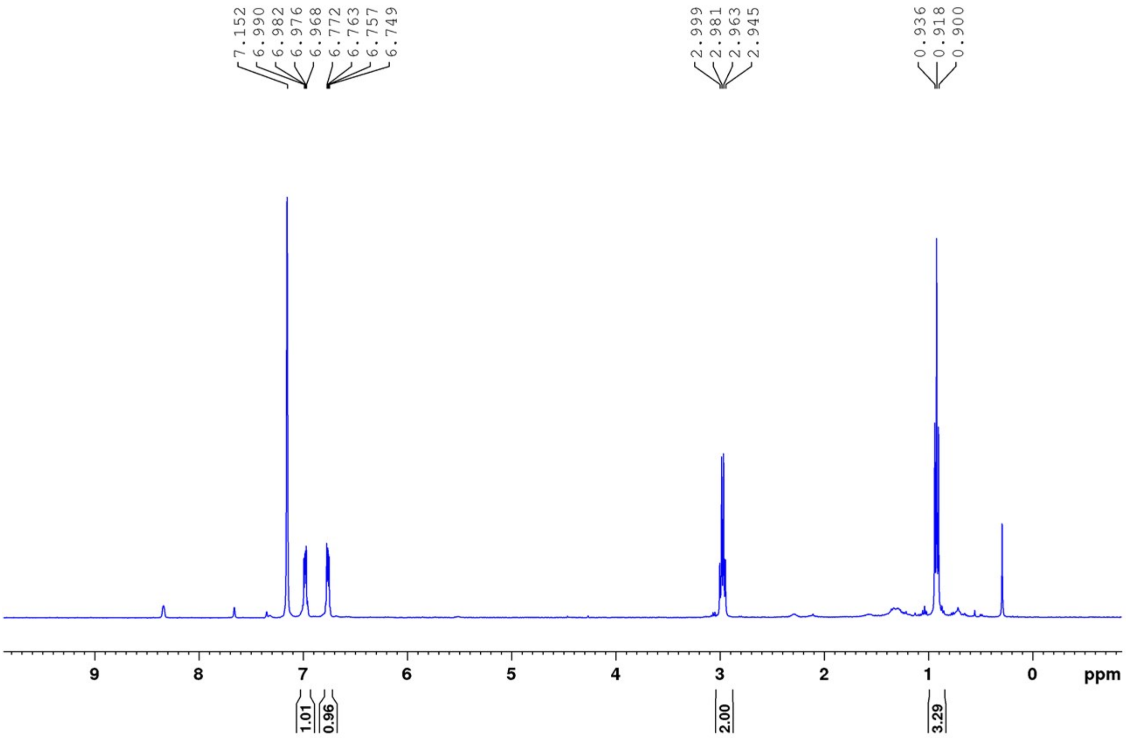

Figure S25: ^{1}H NMR spectrum of the reaction of HBcat with diethylamine catalyzed by complex 8

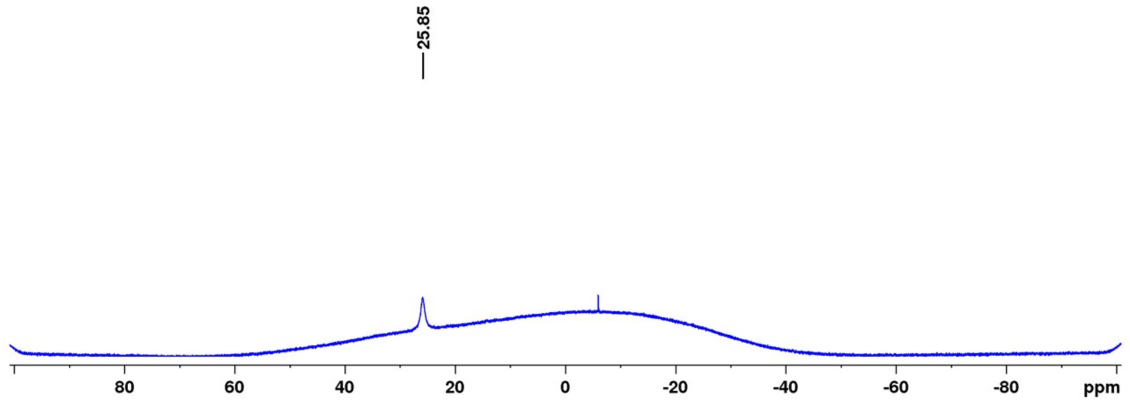

Figure S26: ^{11}B NMR spectrum of the reaction of HBcat with diethylamine catalyzed by complex 8

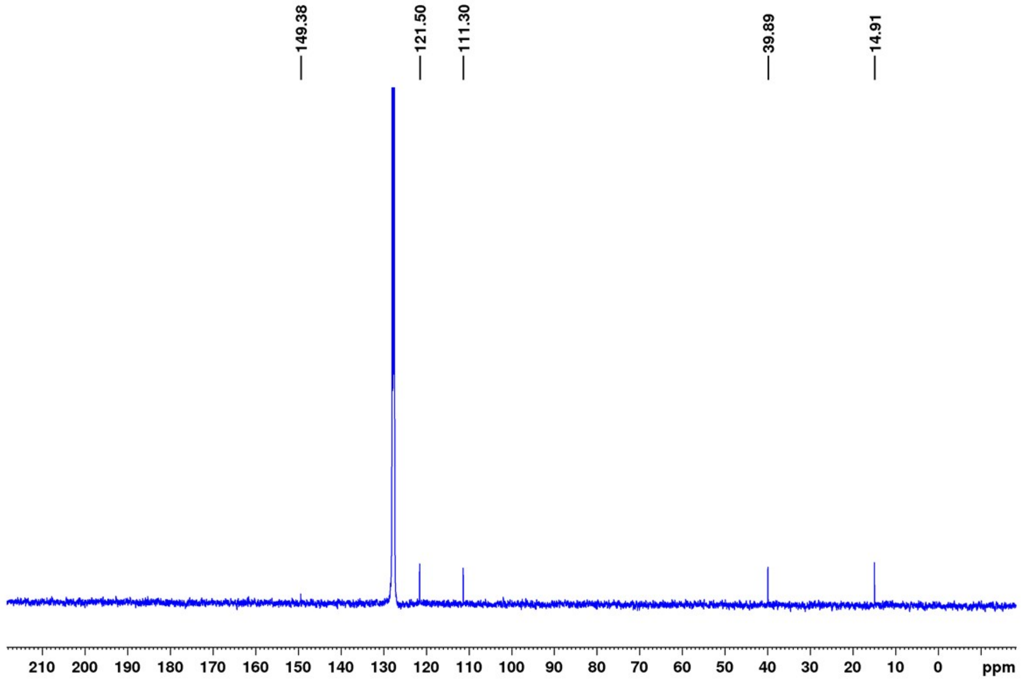

Figure S27: ^{13}C NMR spectrum of the reaction of HBcat with diethylamine catalyzed by complex 8

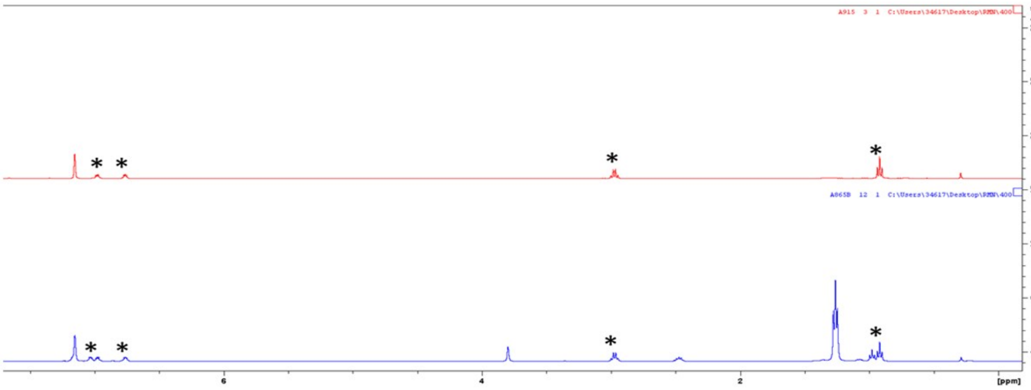

Figure S28: Top (red): ^{1}H NMR spectrum of Et_2NBcat generated by catalytic dehydrocoupling of HBcat and diethylamine; Bottom (blue): ^{1}H NMR spectrum of the reaction of 1 with diethylamine. (* denotes the signals corresponding to Et_2NBcat)

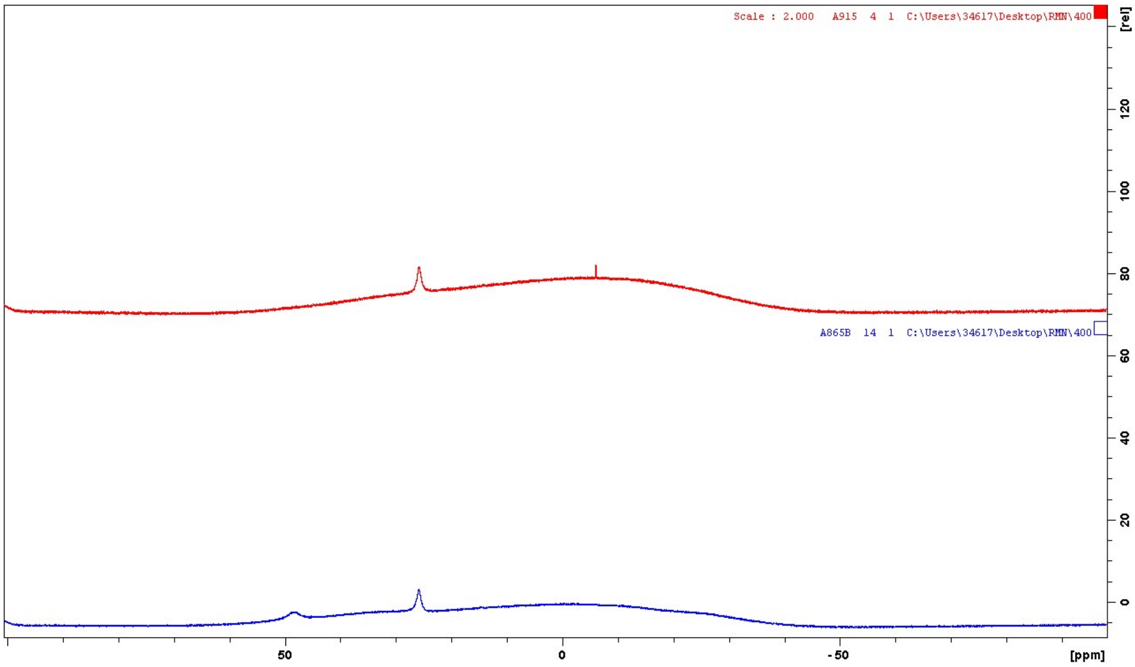

Figure S29: Top (red): ^{11}B NMR spectrum of Et_2NBcat generated by catalytic dehydrocoupling of HBcat and diethylamine; Bottom (blue): ^{11}B NMR spectrum of the reaction of 1 with diethylamine.

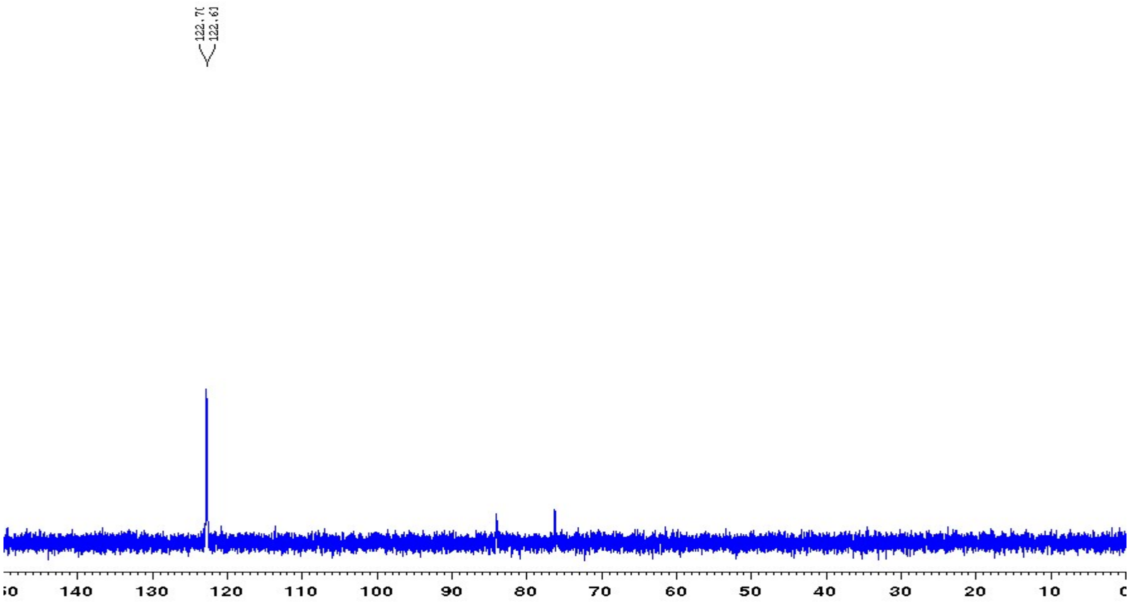

Figure S30: ^{31}P NMR spectrum of the reaction of 1 with Et_2ND

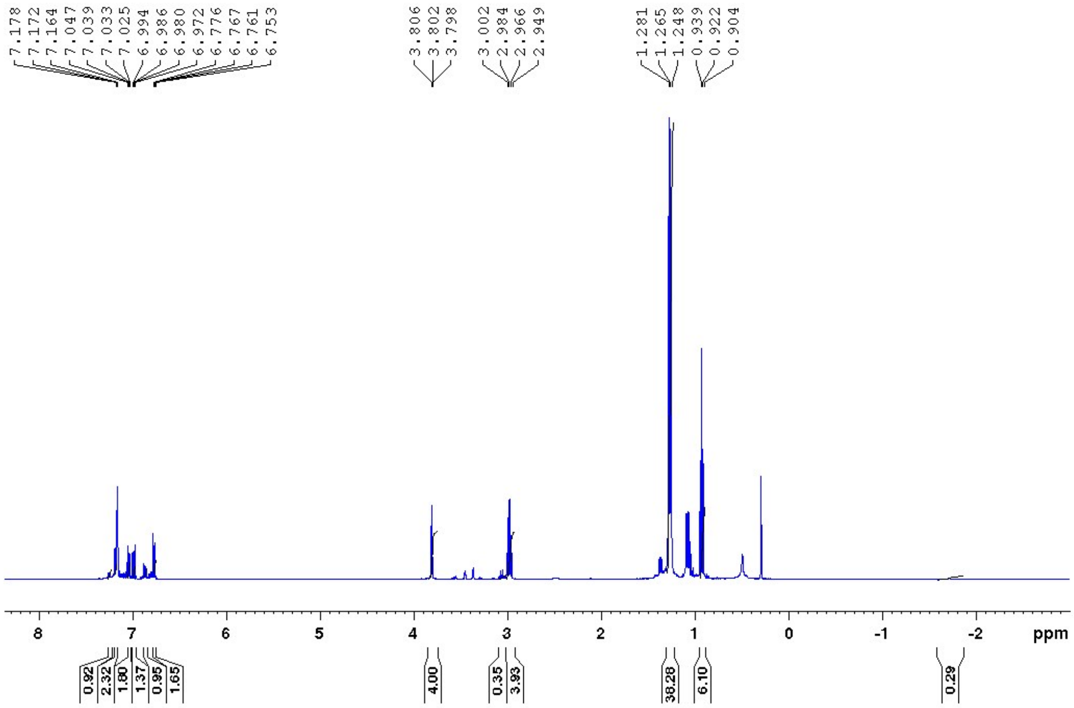

Figure S31: ^{1}H NMR spectrum of the reaction of 1 with Et_2ND (≈80 % deuterated)[3]

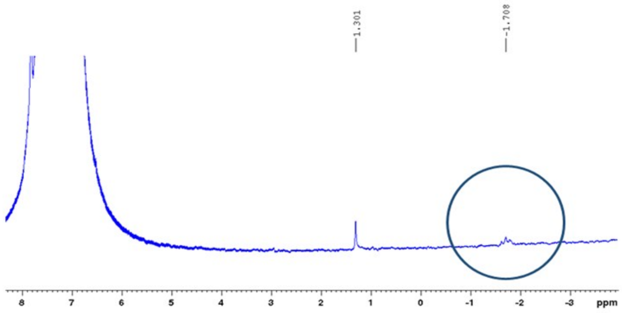

Figure S32: ^{2}H NMR spectrum of the reaction of 1 with Et_2ND; signal at 1.3 ppm corresponds to an unidentified impurity.

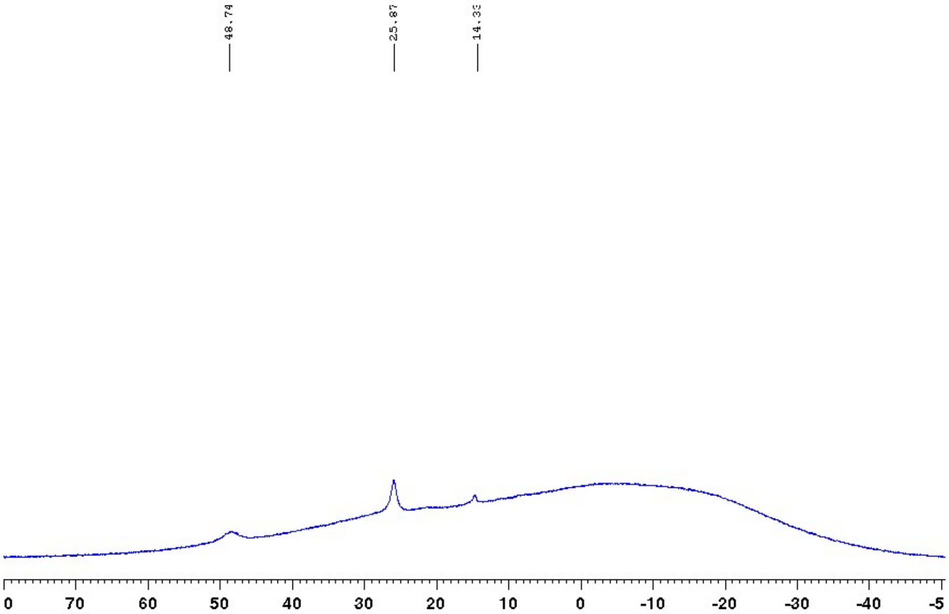

Figure S33: ^{11}B NMR spectrum of the reaction of 1 with Et_2ND

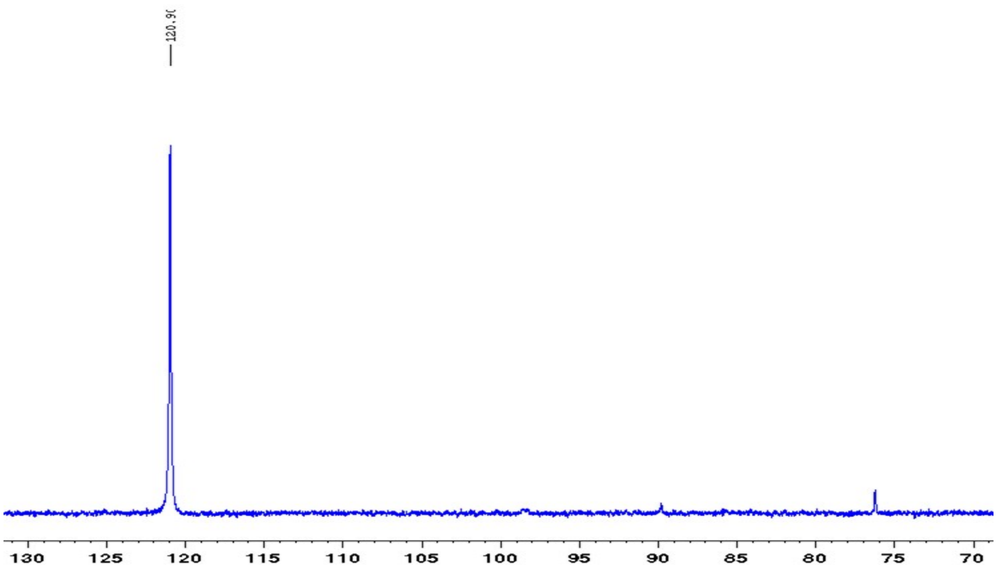

Figure S34: ^{31}P NMR spectrum of the reaction of 1 with NH_3

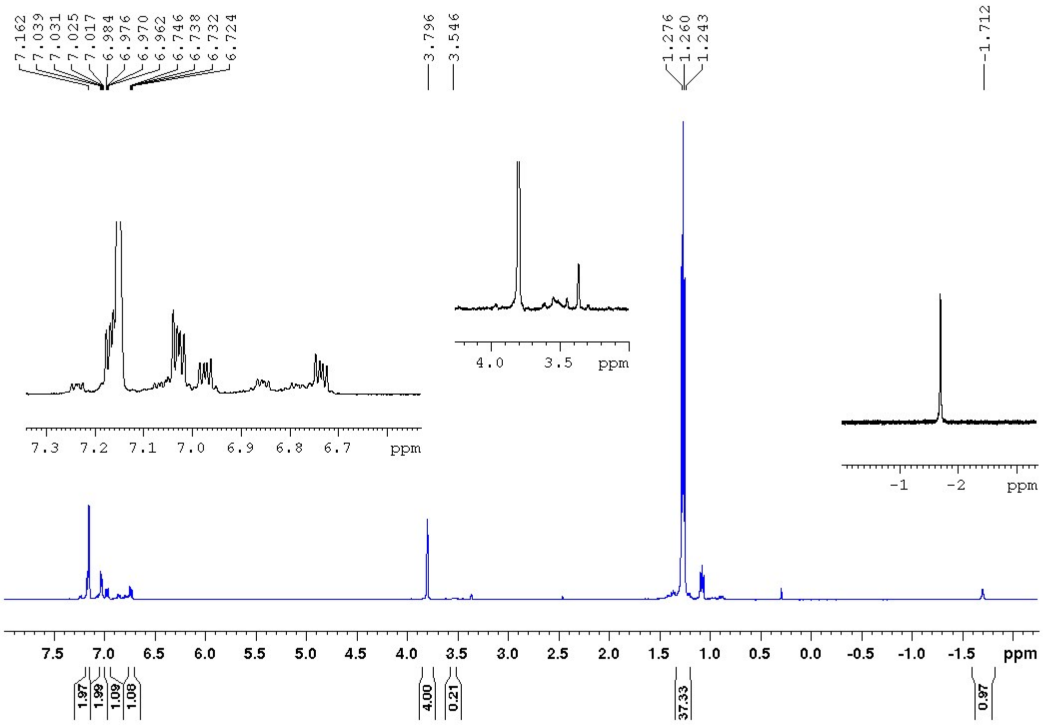

Figure S35: ^{1}H NMR spectrum of the reaction of 1 with NH_3

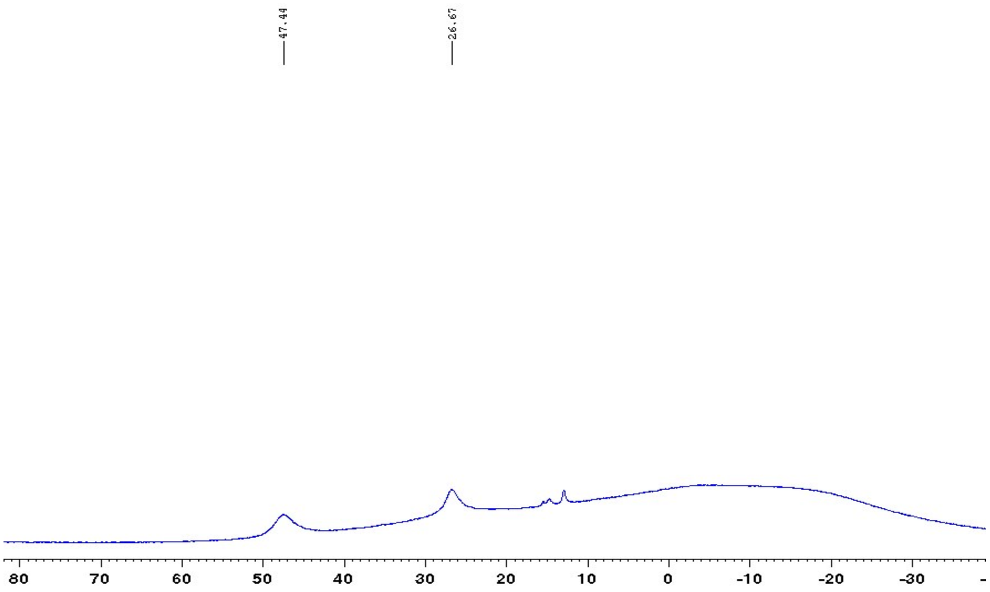

Figure S36: ^{11}B NMR spectrum of the reaction of 1 with NH_3

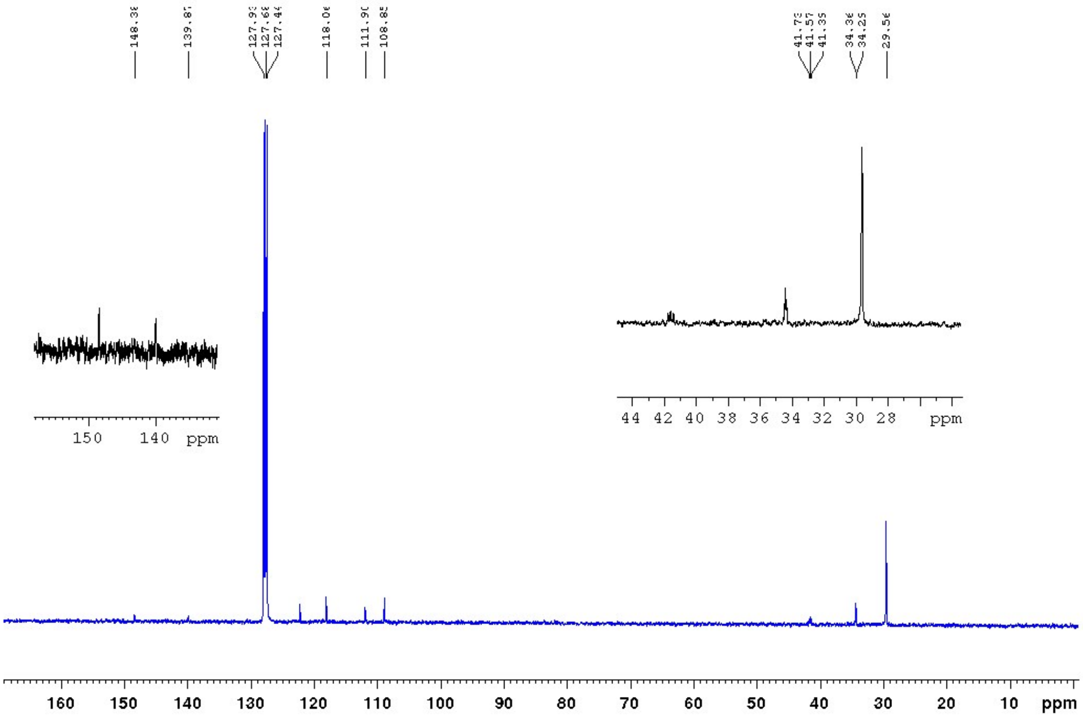

Figure S37: ^{13}C NMR spectrum of the reaction of 1 towards NH_3

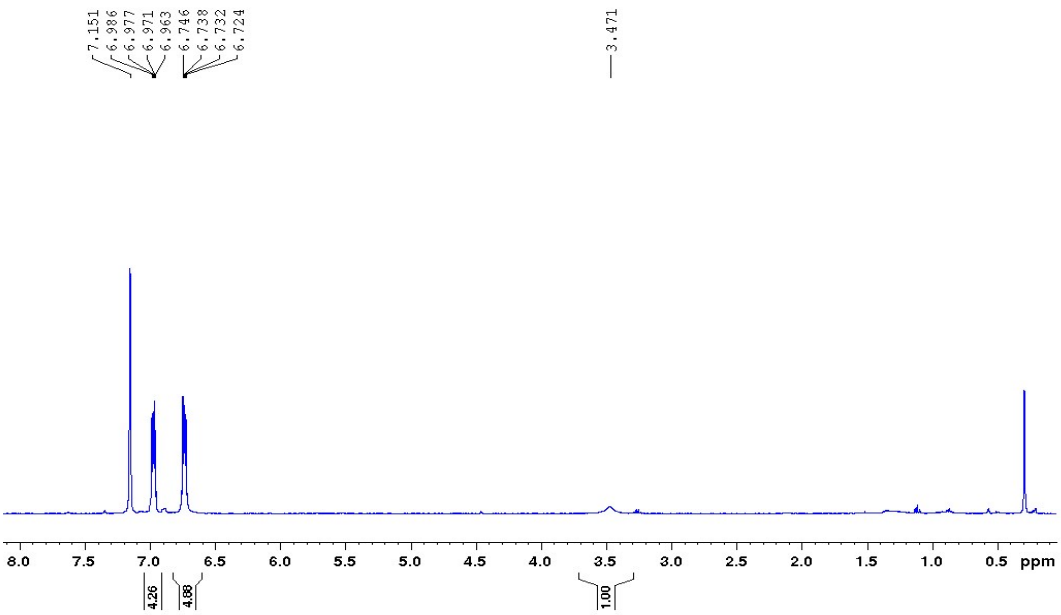

Figure S38: ^{1}H NMR spectrum: $HN(Bcat)_2$ generated by reaction of HBcat and ammonia catalyzed by platinum complex (8).

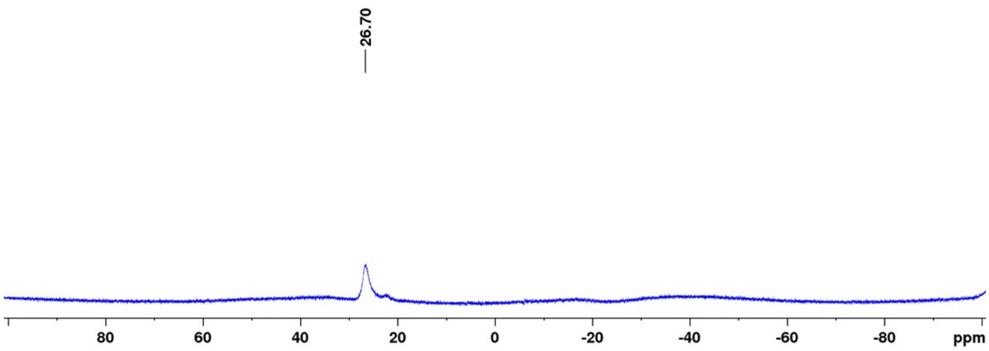

Figure S39: ^{11}B NMR spectrum: $HN(Bcat)_2$ generated by reaction of HBcat and ammonia catalysed by platinum complex 8.

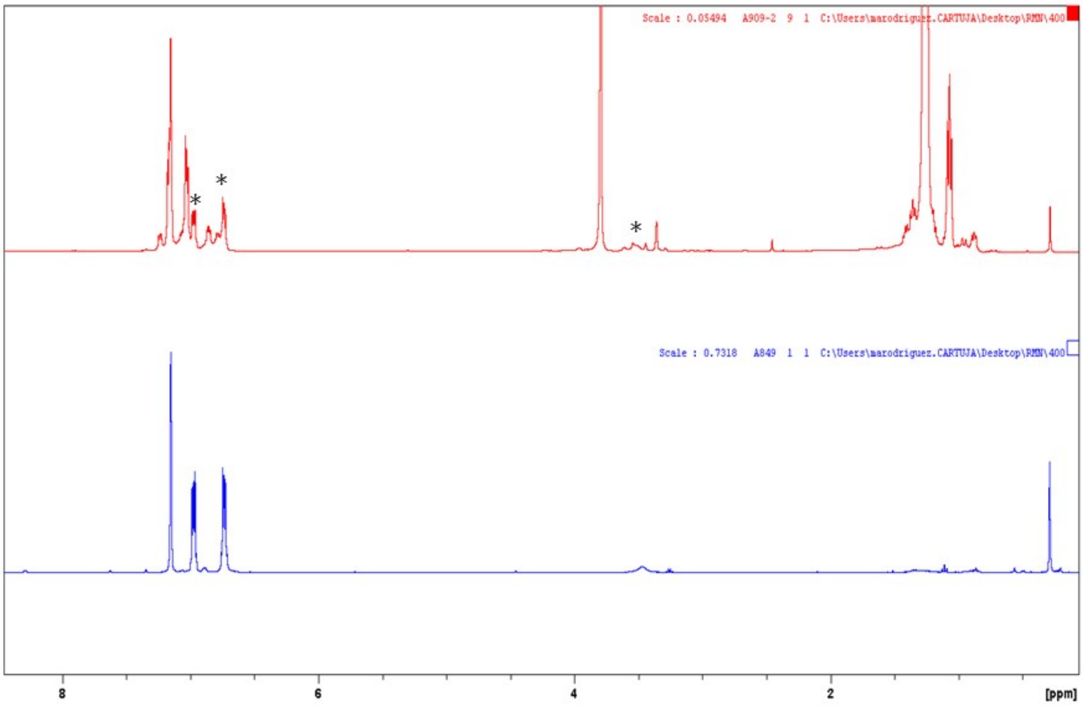

Figure S40: Top (red): ^{1}H NMR spectrum of the reaction of 1 towards NH_3; Bottom (blue): ^{1}H NMR spectrum of $HN(Bcat)_2$ generated by catalytic dehydrocoupling of HBcat and ammonia using 8 as catalyst.

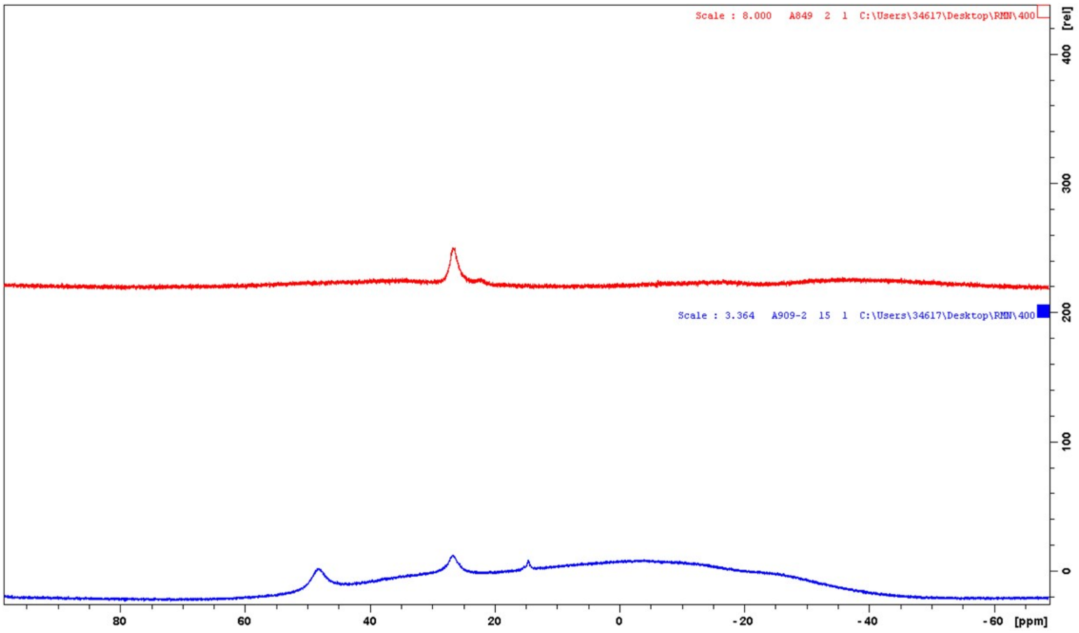

Figure S41: Top (red): ^{11}B NMR spectrum of $HN(Bcat)_2$ generated by catalytic dehydrocoupling of HBcat and ammonia catalysed by 8; Bottom (blue): ^{11}B NMR spectrum of the reaction of 1 towards NH_3.

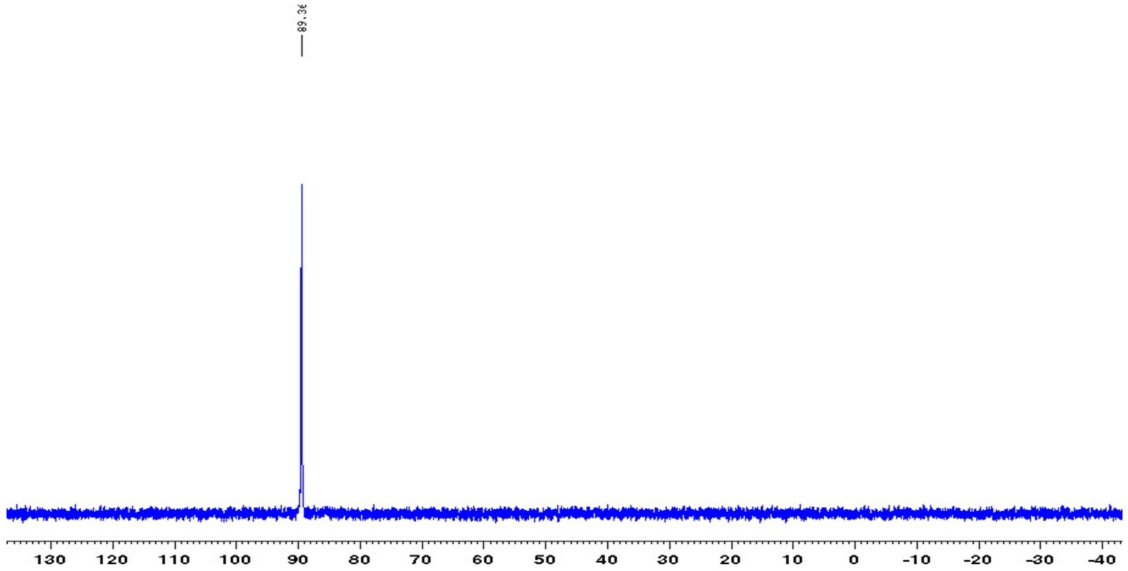

Figure S42: ^{31}P NMR spectrum of the by-product 9 formed during the reaction of 1 with ammonia used as received without previous drying (CD_2Cl_2).

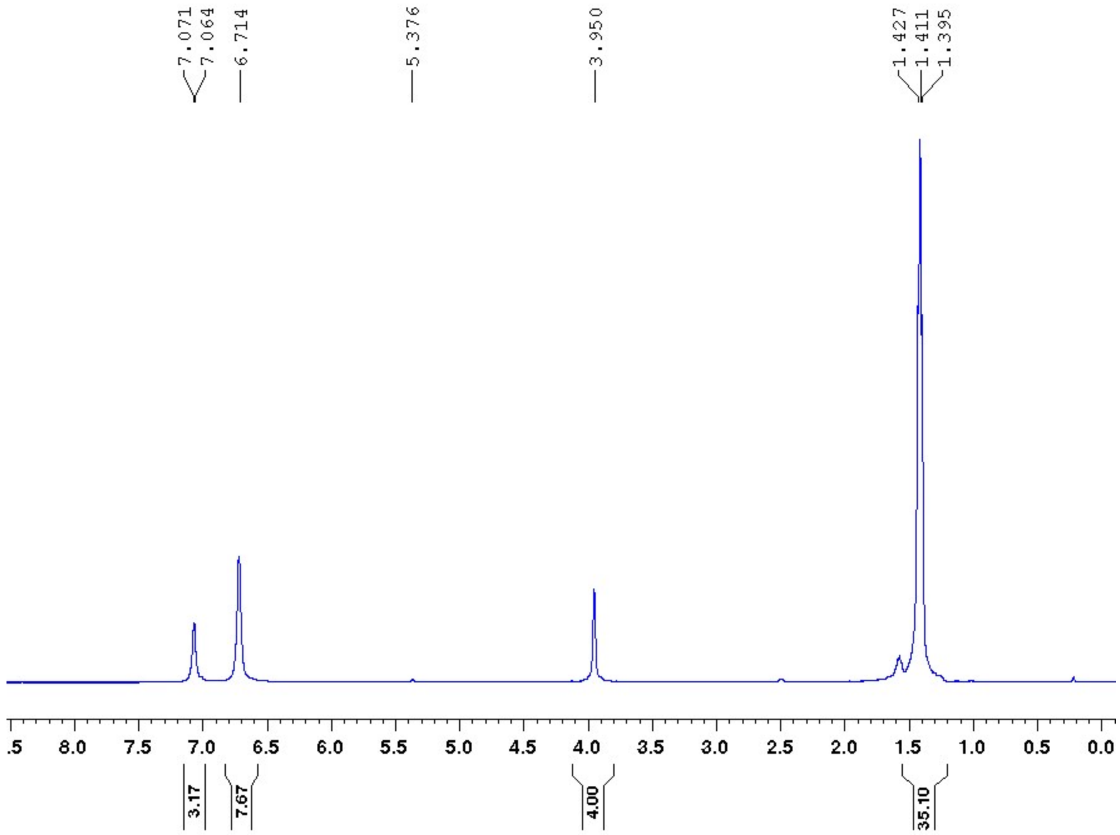

Figure S43: ^{1}H NMR spectrum of the by-product 9 formed during the reaction of 1 with ammonia used as received without previous drying. (CD_2Cl_2).

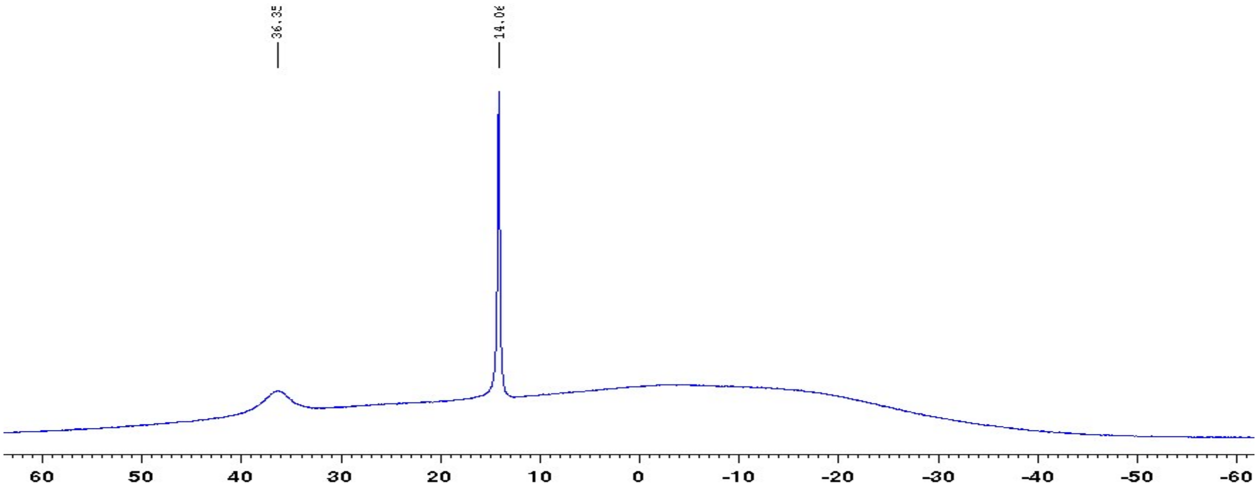

Figure S44: ^{11}B NMR spectrum of the by-product 9 formed during the reaction of 1 with ammonia used as received without previous drying. (CD_2Cl_2)

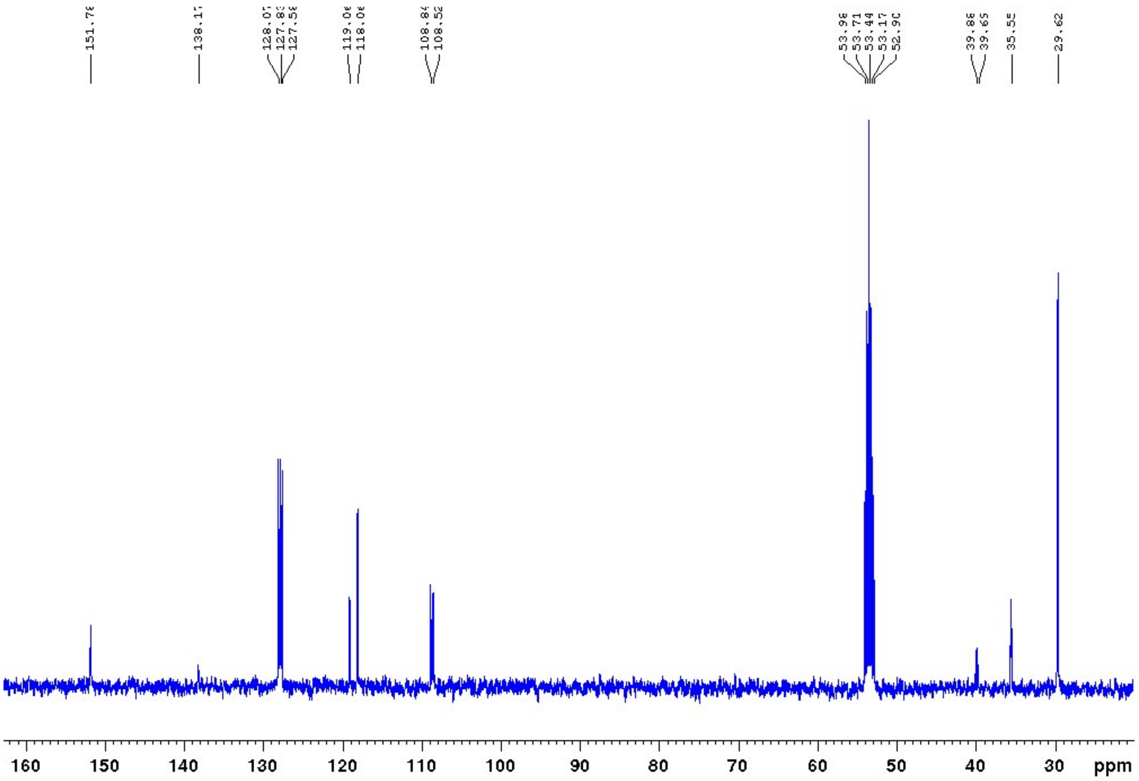

Figure S45: ^{13}C NMR spectrum of the by-product 9 formed during the reaction of 1 with ammonia used as received without previous drying. (CD_2Cl_2)

III-X-RAY STRUCTURAL CHARACTERIZATION

Crystals of **3, 4, 5** and **9** were covered with perfluoropolyether oil (FOMBLIN®, Aldrich) and mounted in a fiber loop. Low-temperature diffraction data were collected on a Bruker D8 Quest APEX-III single crystal diffractometer with a Photon III detector and a IµS 3.0 microfocus X-ray source at the Instituto de Investigaciones Químicas, Sevilla. Data were collected by means of ω and ϕ scans using monochromatic radiation λ (Mo Kα1) = 0.71073 Å. The diffraction images collected were processed and scaled using APEX-III v2018.7-2 software. The structures were solved with SHELXT and was refined against F2 on all data by full-matrix least squares with SHELXL[5], using Olex2[6] as graphical interface. All non-hydrogen atoms were refined anisotropically. Hydrogen atoms were included in the model at geometrically calculated positions and refined using a riding model, unless otherwise noted. The isotropic displacement parameters of all hydrogen atoms were fixed to 1.2 times the U value of the atoms to which they are linked (1.5 times for methyl groups).

A summary of the fundamental crystal and refinement data are given in the Table S1 (complex **3**), Table S2 (complex **4**), Table S3 (complex **5**) and Table S4 (complex **9**). Atomic coordinates, anisotropic displacement parameters and bond lengths and angles can be found in the cif file.

Crystallographic data (excluding structure factors) have been deposited with the Cambridge Crystallographic Data Centre with no. CCDC 2040006-2040007-2040008-2040009.

X-Ray crystal structure of [(^{tBu}PBP)NiBCat] (3)

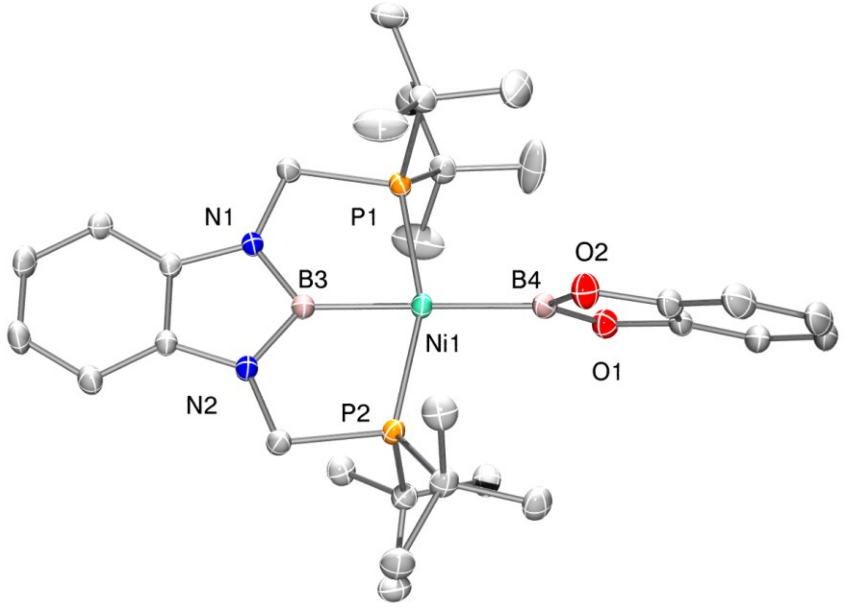

Figure S46. The solid state molecular structure of **[(^{tBu}PBP)NiBCat] (3)**, with thermal ellipsoids set at 50% probability and all hydrogen atoms omitted for clarity. Selected bond lengths (Å) and angles (°): Ni(1)-B(3), 1.942(2); Ni(1)-B(4), 2.015(2); B(3)-N(1), 1.432(4); B(3)-N(2), 1.428(3); Ni(1)-P(1), 2.1700(8); Ni(1)-P(2), 2.1630(8); B(3)-Ni(1)-B(4), 175.0(1); P(2)-Ni(1)-P(1), 157.74(3).

Table S1. Crystal data and structure refinement for **3**

Identification code	ar00419a	
Empirical formula	$C_{30}H_{48}B_2N_2NiO_2P_2$	
Formula weight	610.97	
Temperature/K	193.0	
Crystal system	monoclinic	
Space group	C2/nc	
Unit cell dimensions	a = 22.532(2) Å	α= 90°.
	b = 11.3598(9) Å	β=104.066 (4)°
	c = 26.302(2) Å	γ = 90°.
Volume	6530.4(10) Å^3	
Z	8	
ρ_{calc}g/cm^3	1.243	

μ/mm^{-1}	0.721
F (000)	2608.0
Crystal size	0.17 x 0.13 x 0.13 mm^3
Theta range for data collection	4.04 to 55.698°.
Index ranges	-29 ≤ h ≤ 29, -14 ≤ k ≤ 14, -34 ≤ l ≤ 34
Reflections collected	189783
Independent reflections	7541 [R_{int} = 0.0875, R_{sigma} = 0.0371]
Data/restraints/parameters	7541/18/364
Goodness-of-fit on F^2	1.149
Final R indices [I>2sigma(I)]	R1 = 0.0397, wR2 = 0.0807
R indices (all data)	R1 = 0.0650, wR2 = 0.0961
Largest diff. peak and hole	0.88/-0.29 e.Å^{-3}

X-Ray crystal structure of [(^{tBu}PBP)NiH$_2$Bcat] (4)

The structure was solved by Patterson interpretation and phase extension using DIRDIF2008[7]. Isotropic least-squares refinement on F^2 using SHELXL-2018/3[8] was performed. During the final stages of the refinement, all the positional and anisotropic displacement parameters of all non-H atoms were refined. The H atoms (except those labeled as H1 and H2) were geometrically located and their coordinates were refined riding on their parent atoms. The final positions of the hydrogen atoms of all methyl groups were obtained after a rotating group refinement (the initial torsion angle is derived from a difference Fourier synthesis). H1 and H2 were found from the corresponding difference Fourier map and included in the refinement with isotropic displacement parameters. The crystal suffers from positional disorder. Fortunately, SHELXL-2018/3[8] handles this situation without difficulty with the aid of the PART and FVAR instructions to estimate the ratio of the two components in the crystal. Once completed the refinement, it is concluded that the ratio between the two components is 0.77:0.33 (atoms H1 and H2 could not be located in the minority component). Furthermore, the crystal is actually an aggregate of two individual crystals. PLATON[9]

TwinRotMat algorithm proposed a twin law for the crystal whose use significantly improved the refinement results. The majority domain represents 0.64 of the total.

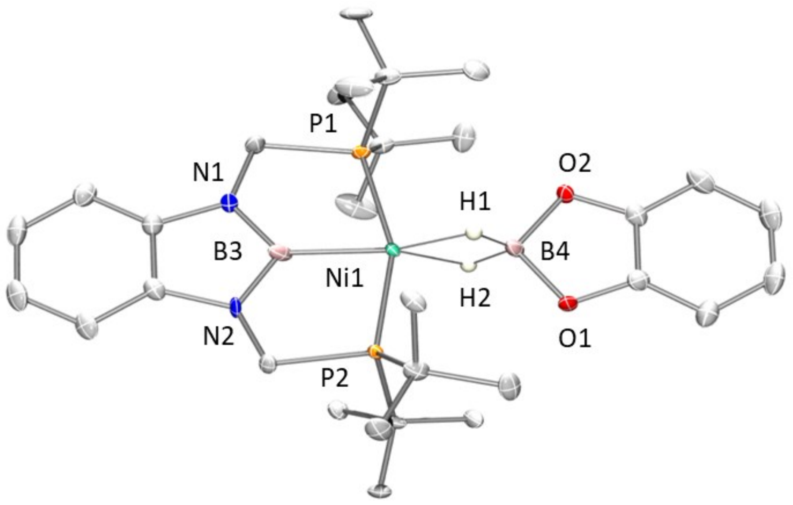

Figure S47. The solid state molecular structure of **[(^{tBu}PBP)NiH$_2$Bcat] (4)**, with thermal ellipsoids set at 50% probability and all hydrogen atoms omitted for clarity. Selected bond lengths (Å) and angles (°): Ni (1)–B (4) = 2.145 (3); Ni (1)–B (3) = 1.966 (2); B (4)–H (1) = 1.190 (2); B (4)–H (2) = 1.267(3); Ni(1)–H(1) = 1.614 (3); Ni(1)–H(2) = 1.750 (2).

Table S2. Crystal data and structure refinement for **4**

Identification code	ar00320a_0m	
Empirical formula	$C_{30}H_{50}B_2N_2NiO_2P_2$	
Formula weight	612.99	
Temperature/K	100.0	
Crystal system	monoclinic	
Space group	Pc	
Unit cell dimensions	a = 9.2940(5) Å	α= 90°.
	b = 15.3277(7) Å	β= 107.920(2)°
	c = 11.7365(5) Å	γ = 90°.
Volume	1590.8(1) Å^3	
Z	2	
ρ_{calc}g/cm^3	1.280	
μ/mm^{-1}	0.39	
F(000)	656	

Crystal size	0.18 x 0.17 x 0.05 mm3
Theta range for data collection	1.8 to 23.7°.
Index ranges	-13 ≤ h ≤ 13, - ≤18 k ≤ 21, -16 ≤ l ≤ 16
Reflections collected	17565
Independent reflections	8805 [R_{int} = 0.028, R_{sigma} = 0.044]
Data/restraints/parameters	7840/2/486
Goodness-of-fit on F^2	1.053
Final R indices [I>2sigma(I)]	R1 = 0.0425, wR2 = 0.0988
R indices (all data)	R1 = 0.0504, wR2 = 0.1029
Largest diff. peak and hole	0.47/-0.35 e.Å^{-3}

X-Ray crystal structure of [C_4H_4NBCat]$_2$ (5)

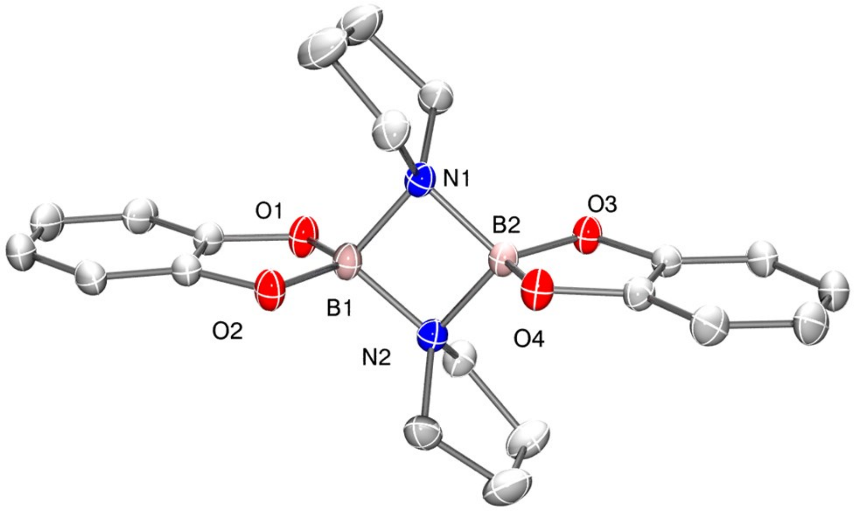

Figure S48. The solid state molecular structure of **[C_4H_4NBCat]$_2$ (5)** with thermal ellipsoids set at 50% probability and all hydrogen atoms omitted for clarity. Selected bond lengths (Å) and angles (°): N(1)-B(2), 1.584(2); N(2)-B(2), 1.622(2); B(1)-N(2), 1.584(2); B(1)-N(1), 1.618(2).

**Table S3**. Crystal data and structure refinement for **5**		
Identification code	ar00320a_0m	
Empirical formula	$C_{20}H_{24}B_2N_2O_4$	
Formula weight	378.03	
Temperature/K	208.0	
Crystal system	monoclinic	
Space group	$P2_1/n$	
Unit cell dimensions	a = 9.273(2) Å	α= 90°.
	b = 18.201(4) Å	β= 106.121(8)°
	c = 11.690(3) Å	γ = 90°.
Volume	1895.4(7) Å^3	
Z	4	
ρ_{calc}g/cm^3	1.325	
μ/mm^{-1}	0.090	
F(000)	800	
Crystal size	0.9 x 0.6 x 0.5 mm^3	
Theta range for data collection	4.262 to 60.966°.	
Index ranges	-13 ≤ h ≤ 12, -25 ≤ k ≤ 25, -16 ≤ l ≤ 16	
Reflections collected	24762	
Independent reflections	5723 [R_{int} = 0.0483, R_{sigma} = 0.0423]	
Data/restraints/parameters	5723/0/253	
Goodness-of-fit on F^2	1.047	
Final R indices [I>2sigma(I)]	R1 = 0.0522, wR2 = 0.1393	
R indices (all data)	R1 = 0.0702, wR2 = 0.1548	
Largest diff. peak and hole	0.47/-0.30e.Å^{-3}	

X-Ray crystal structure of [(^{tBu}PBP)Ni(NH_3)][$BCat_2$] (9)

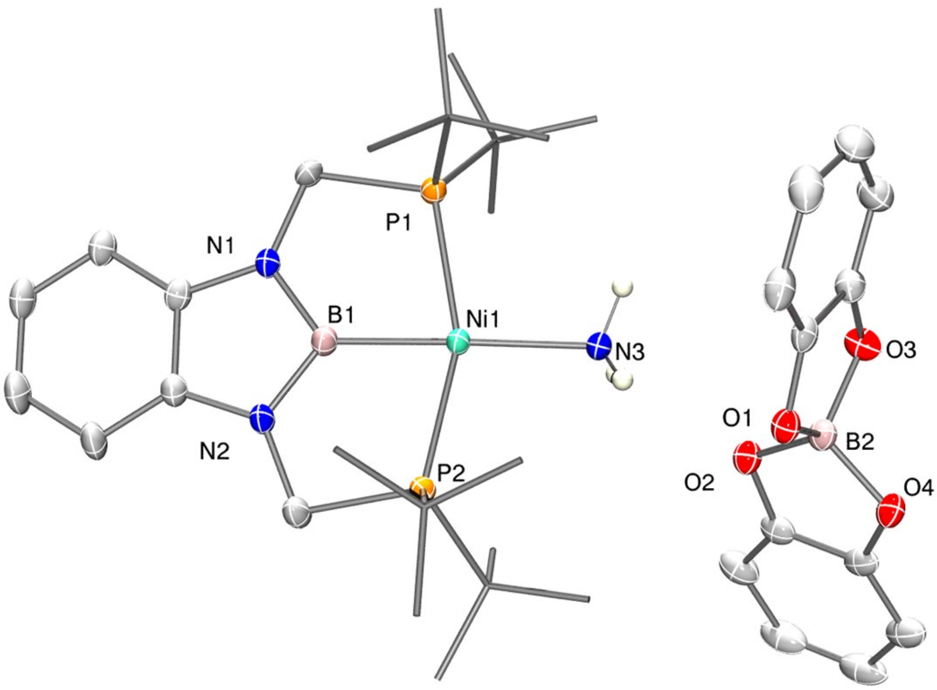

Figure S49. The solid state molecular structure of [(^{tBu}PBP)NiNH_3][$Bcat_2$] (**9**), with thermal ellipsoids set at 50% probability and all hydrogen atoms omitted for clarity. Selected bond lengths (Å) and angles (°): Ni(1)-B(1), 1.913(4); Ni(1)-N(3), 2.001(3); B(1)-N(1), 1.434(4); B(1)-N(2), 1.423(4); Ni(1)-P(1), 2.2238(8); Ni(1)-P(2), 2.2209(9); B(1)-Ni(1)-N(3), 173.7(1); P(2)-Ni(1)-P(1), 156.91(3).

Table S4. Crystal data and structure refinement for 9

Identification code	ar00220a_0m	
Empirical formula	$C_{36}H_{55}B_2N_3O_4P_2$	
Formula weight	736.10	
Temperature/K	193.0	
Crystal system	orthorhombic	
Space group	$P2_12_12$	
Unit cell dimensions	a = 17.5213(7) Å	α= 90°.
	b = 20.1284(7) Å	β= 90°
	c = 12.3444(5) Å	γ = 90°.
Volume	4353.6(3) Å^3	

Z	4
ρ_{calc}g/cm^3	1.123
μ/mm^{-1}	0.555
F(000)	1568.0
Crystal size	0.33 x 0.11 x 0.04 mm^3
Theta range for data collection	4.036 to 56.602°.
Index ranges	-23 ≤ h ≤ 23, -26 ≤ k ≤ 25, -16 ≤ l ≤ 16
Reflections collected	35869
Independent reflections	10775 [R_{int} = 0.0485, R_{sigma} = 0.0567]
Data/restraints/parameters	10775/0/446
Goodness-of-fit on F^2	1.005
Final R indices [I>2sigma(I)]	R1 = 0.0377, wR2 = 0.0884
R indices (all data)	R1 = 0.0479, wR2 = 0.0931
Largest diff. peak and hole	0.23/-0.25e.Å^{-3}
Flack parameter	0.007(6)

IV- COMPUTATIONAL DETAILS

Calculations were performed using the PBE0 functional [10] including the D3 version of Grimme's dispersion correction [11], as implemented in Gaussian 09.[12] Geometry optimizations were performed in solution (solvent = toluene) using the continuum SMD[13] and basis set 1 (BS1). This basis set uses the double-ζ 6-31G(d,p) basis set for the H, C, N, B, O and P atoms and the scalar relativistic Stuttgart−Dresden SDD pseudopotential and its associated double-ζ basis set, complemented with a set of *f* polarization functions, for the Ni atom.[14] The nature of the stationary points was confirmed by frequency analysis. Connections between the transition states and the minima were checked by following the IRC and subsequent geometry optimization until the corresponding minima. All energies in solution were corrected by single-point calculations with the larger basis set 2 (BS2) consisting of the *def2*TZVP basis set for the main group elements, combined with *def2*QZVP basis set for Ni.[15] Gibbs energies in toluene were calculated at 298.15 K. Gibbs energy corrections were obtained based

on vibrational frequencies of the BS1 or structures using the quasi-harmonic approximation. Thermal contributions to the Gibbs energies were corrected by employing the approximation described by Grimme where entropic terms for frequencies below a cut-off of 100 cm^{-1}, were calculated using the free-rotor approximation. [16] The GoodVibes program developed by Paton and Funes-Ardoiz was employed to introduce these corrections. [17] All reported energies in the main text correspond to PBE0-D3/BS2 Gibbs energies in toluene at 298.15K in kcal mol^{-1}. QTAIM[18] and orbital composition studies were conducted with Multiwfn software.[19] Structure and orbital visualization were performed with Chemcraft software.[20] 3D-structures in Figure 6 were generated using CYLview.[21]

Orbital localization of canonical Density Functional Theory orbitals was performed with the CP2K code.[22] The PBE exchange-correlation functional was used.[23] The Quickstep algorithm was used to solve the electronic structure problem,[24] employing a double zeta plus polarization (DZVP) basis set to represent the valence orbitals and plane waves for the electron density (300 Ry cutoff). Goedecker-Teter-Hutter (GTH) type pseudopotentials were used for valence core interactions.[25] Models were treated as isolated in a cubic box of 30 Å edge.

V – COMPARISON OF X-RAY AND OPTIMIZED GEOMETRICAL PARAMETER OF COMPLEX 3

Parameter	Computed structure	Crystal structure
**Ni-B(Bcat)**	1.999 Å	2.015(2) Å
**Ni-B(PBP)**	1.948 Å	1.942(2) Å
**P-Ni-P**	157.27°	157.74(3)°
**B-Ni-B**	179.99°	175.0(1)°
**N-B-B-O**	-103.5°	103.5(3)°
**N-B-N**	104.89°	104.7(2)°
**O-B-O**	106.38°	107.5(2)°

Table S5. Comparison of the computed and experimental geometrical parameters for complex **3**

VI – GEOMETRICAL PARAMETERS OF INTERMEDIATES AND TRANSITION STATES IN THE ENERGY PROFILE OF FIGURE 5

Species 3·H_2

Parameter	Value
H88-H89	0.803 Å
Ni1-B15	1.947 Å
B15-H89	2.387 Å
Ni1-H89	1.747 Å
Ni1-H88	1.744 Å
Ni1-B2	1.976 Å
B2-H88	2.407 Å
B15-H89-Ni1	53.5 °
H89-B15-Ni1	46.2 °
H89-Ni1-H88	26.6 °
H88-B2-Ni1	45.6 °
B2-H88-Ni1	54.1 °
B15-Ni1-B2	172.5 °
Sum of angles around B15	359.6 °
Sum of angles around B2	360.0 °

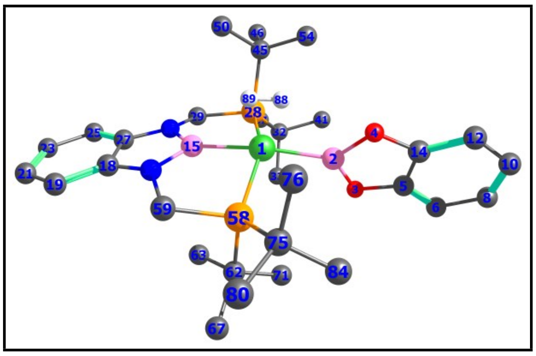

Species TS1

Parameter	Value
H88-H89	1.244 Å
Ni1-B15	1.958 Å
B15-H89	1.632 Å
Ni1-H89	1.503 Å
Ni1-H88	1.495 Å
Ni1-B2	1.932 Å
B2-H88	2.211 Å
B15-H89-Ni1	77.2 °
H89-B15-Ni1	48.4 °
H89-Ni1-H88	49.0 °
H88-B2-Ni1	41.6 °
B2-H88-Ni1	59.1 °
B15-Ni1-B2	176.9 °
Sum of angles around B15	357.6 °
Sum of angles around B2	359.9 °

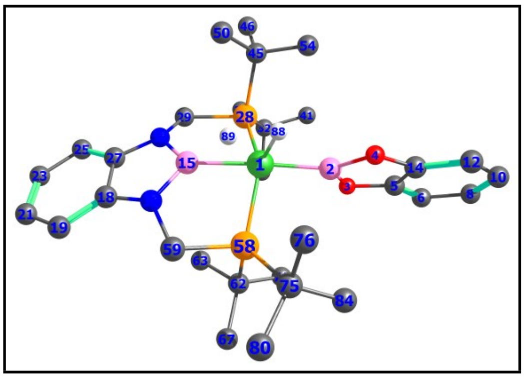

Species Int1

Parameter	Value
H88-H89	2.513 Å
Ni1-B15	2.037 Å
B15-H89	1.284 Å
Ni1-H89	1.636 Å
Ni1-H88	1.614 Å
Ni1-B2	1.979 Å
B2-H88	1.298 Å
B15-H89-Ni1	87.6 °
H89-B15-Ni1	53.3 °
H89-Ni1-H88	101.3 °
H88-B2-Ni1	54.3 °
B2-H88-Ni1	84.9 °
B15-Ni1-B2	179.1 °
Sum of angles around B15	351.7 °
Sum of angles around B2	343.9 °

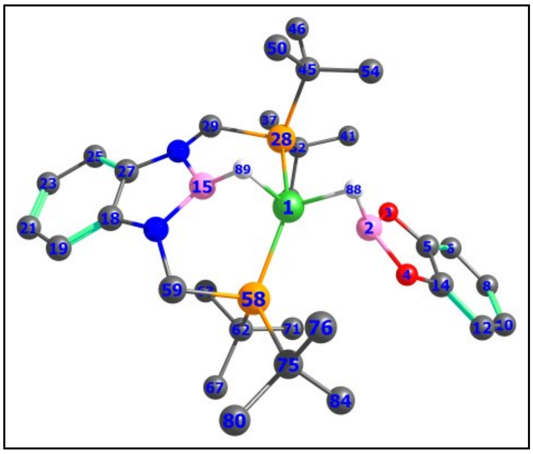

Species Int1’

Parameter	Value
H75-H89	2.800 Å
Ni1-B2	2.054 Å
B2-H89	1.275 Å
Ni1-H89	1.698 Å
Ni1-H75	1.567 Å
Ni1-B76	2.042 Å
B76-H75	1.281 Å
B2-H89-Ni1	86.2 °
H89-B2-Ni1	55.6 °
H89-Ni1-H75	118.0 °
H75-B76-Ni1	50.1 °
B76-H75-Ni1	91.1 °
B2-Ni1-B76	133.0 °
Sum of angles around B2	352.2 °
Sum of angles around B76	346.8 °

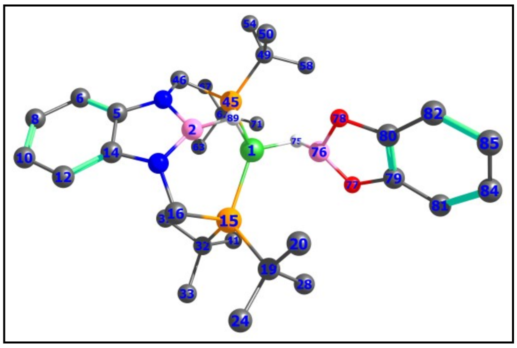

Species TS2

Parameter	Value
H75-H89	2.379
Ni1-B2	1.898
B2-H89	1.703
Ni1-H89	1.515
Ni1-H75	1.619
Ni1-B76	1.993
B76-H75	1.327
B2-H89-Ni1	72.0
H89-B2-Ni1	49.4
H89-Ni1-H75	98.7
H75-B76-Ni1	53.9
B76-H75-Ni1	84.5
B2-Ni1-B76	115.9
Sum of angles around B2	356.9
Sum of angles around B76	339.2

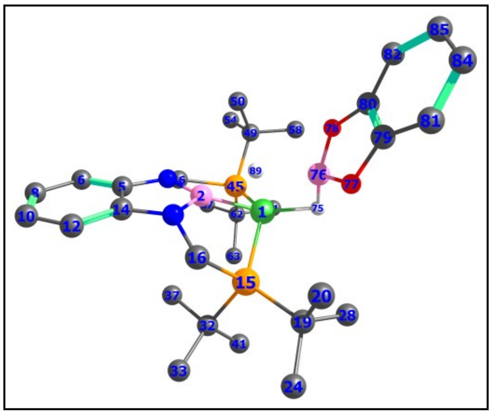

VII– NATURAL CHARGE ANALYSIS OF INTERMEDIATES AND TRANSITION STATES IN THE ENERGY PROFILE OF FIGURE 5

Species 3

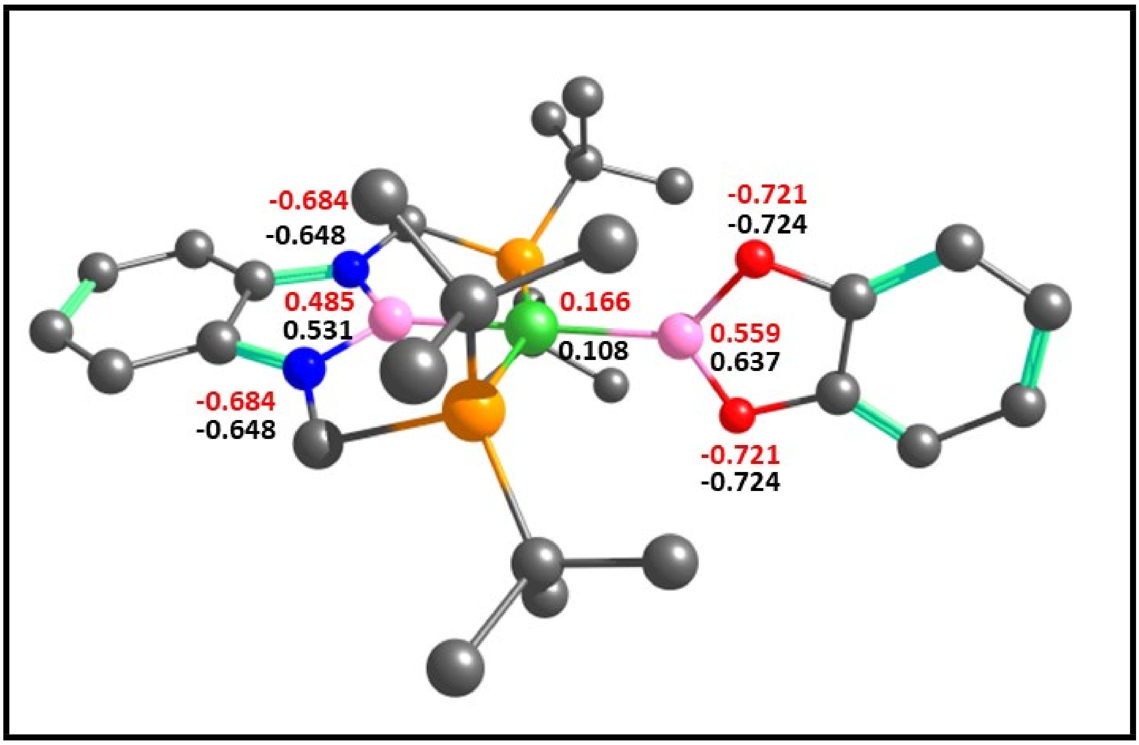

Species 3·H_2

BS1
BS2

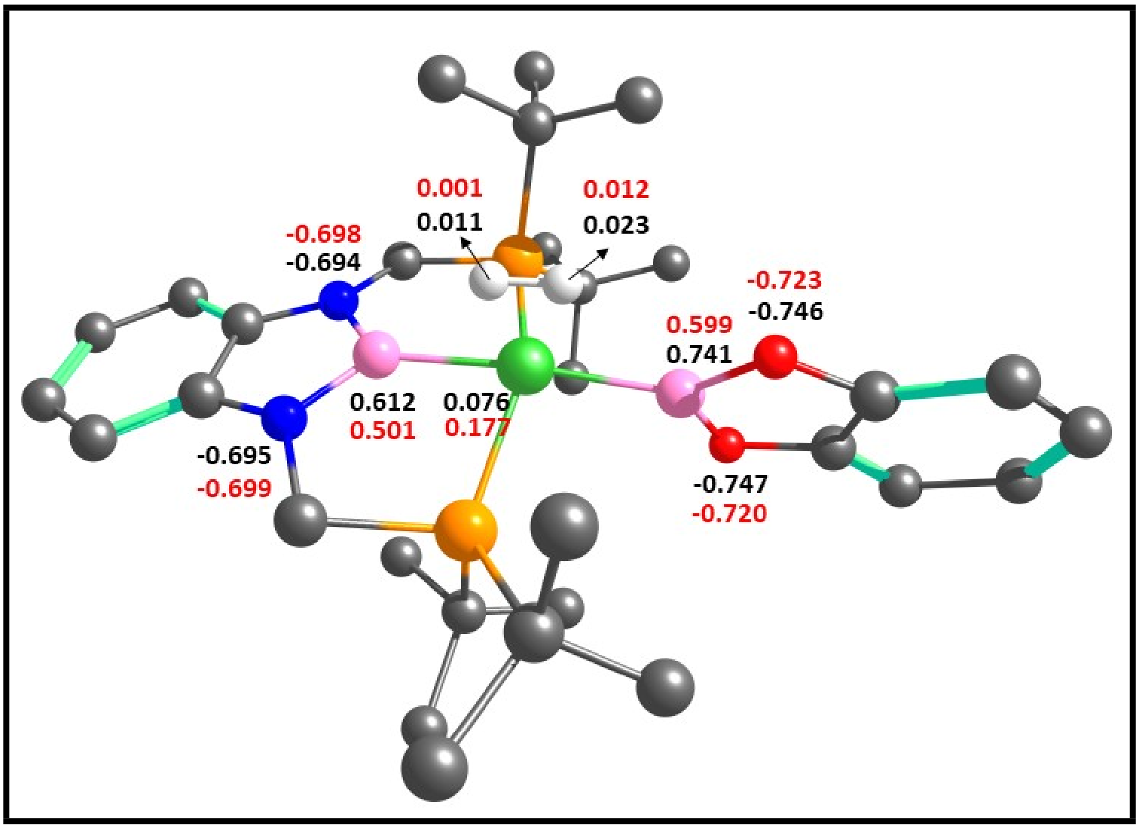

Species TS1

BS1

BS2

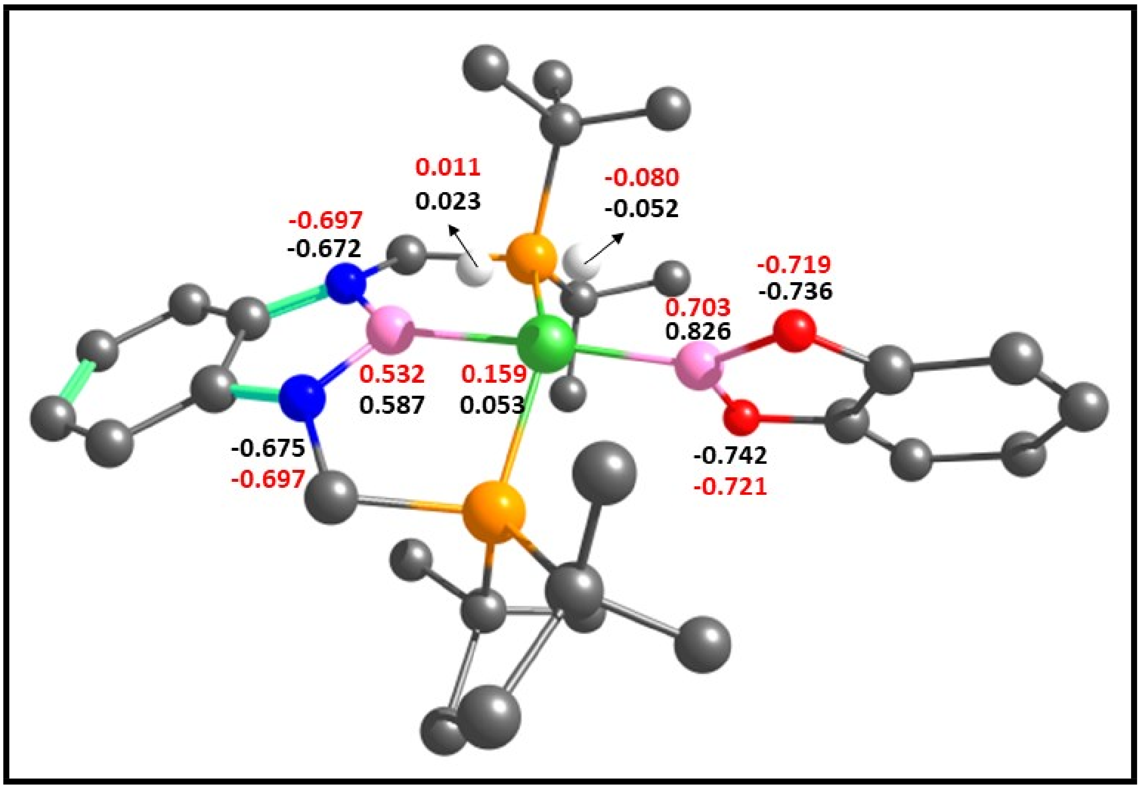

Species Int1

BS1
BS2

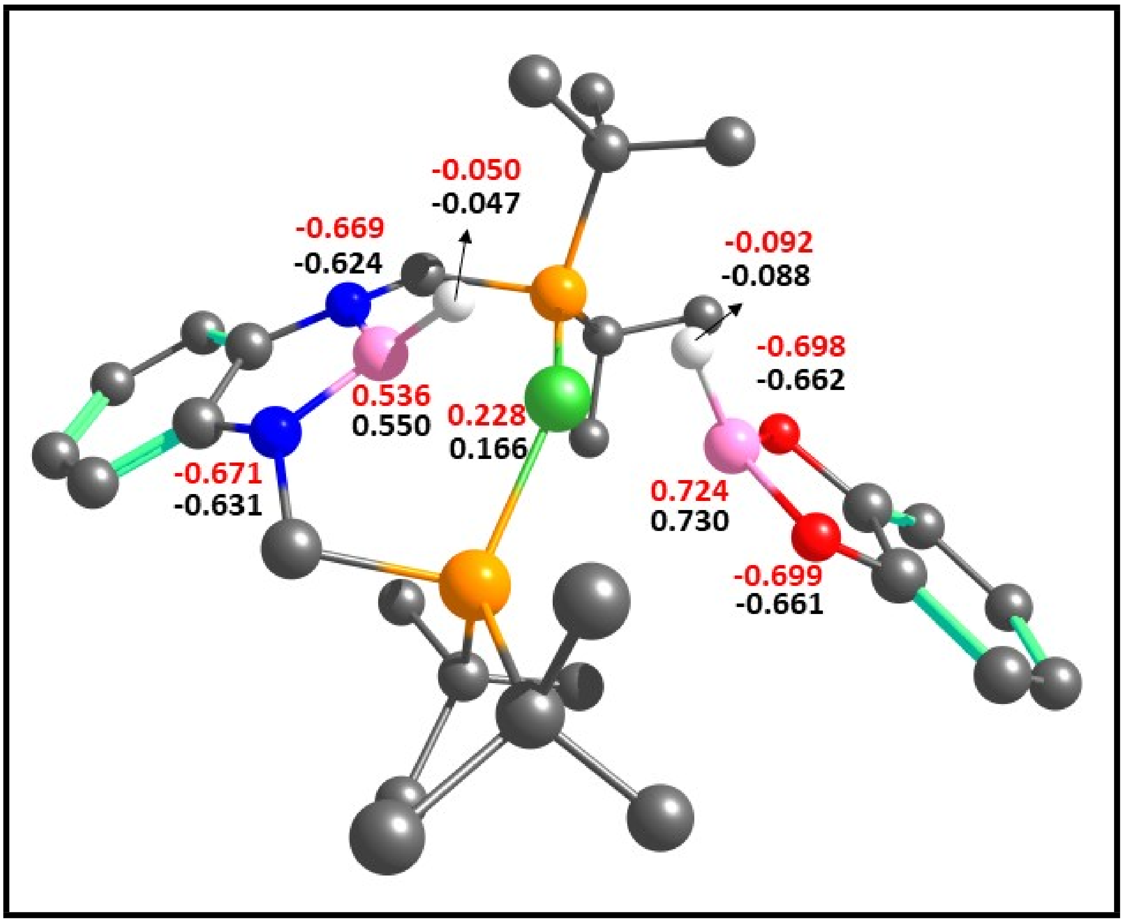

Species Int1’

BS1
BS2

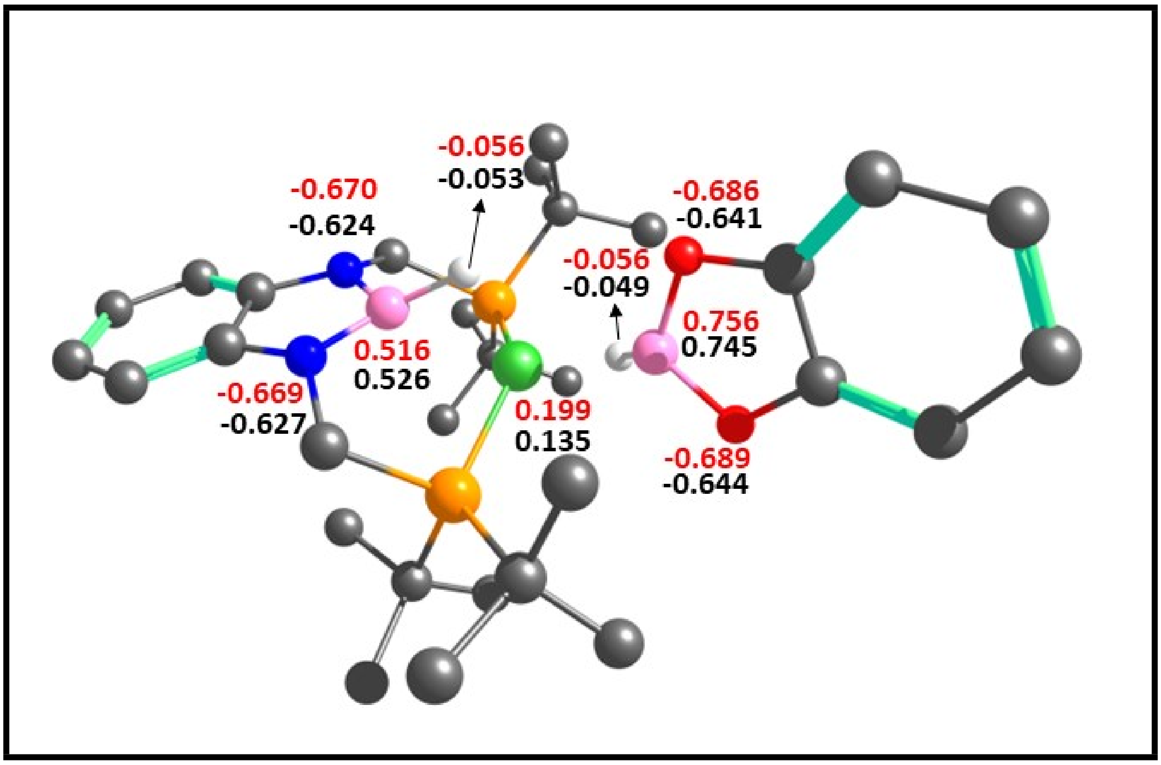

Species TS2

BS1
BS2

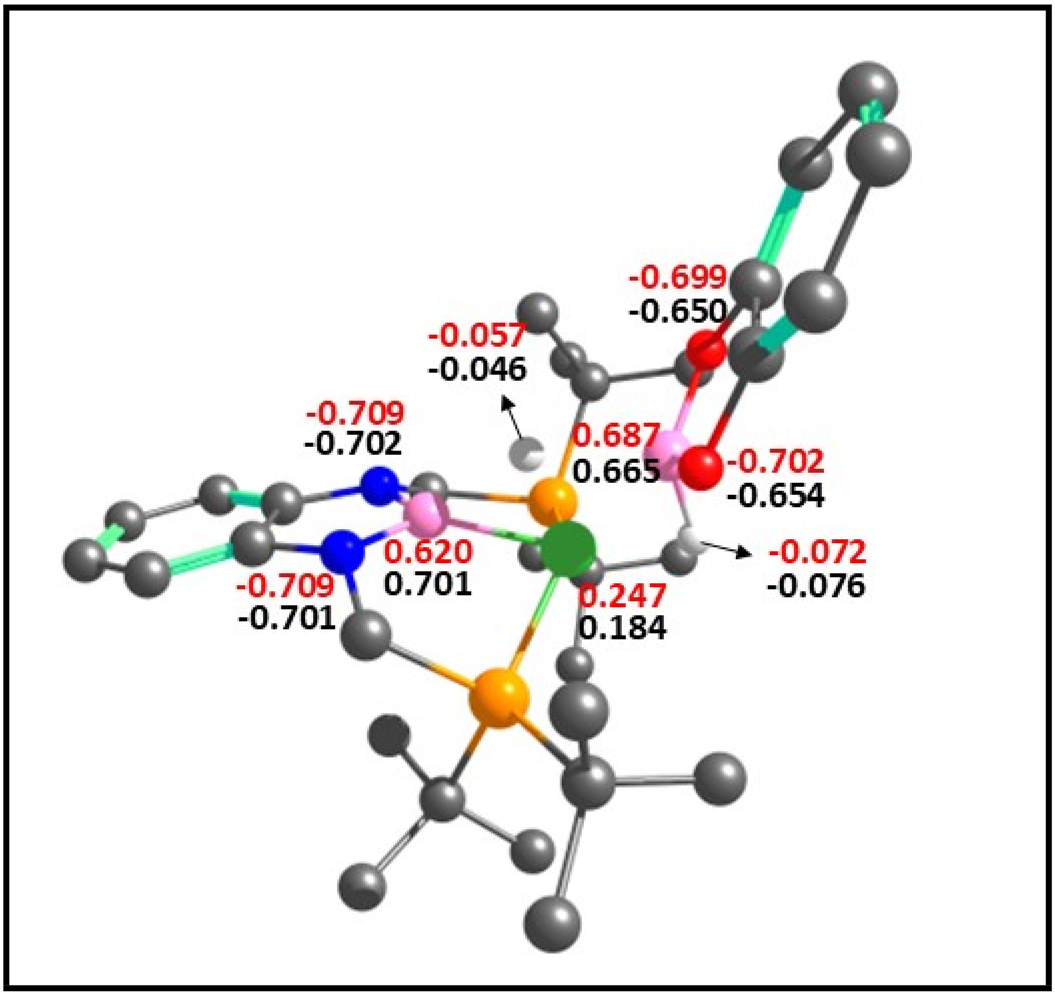

Species 4

BS1
BS2

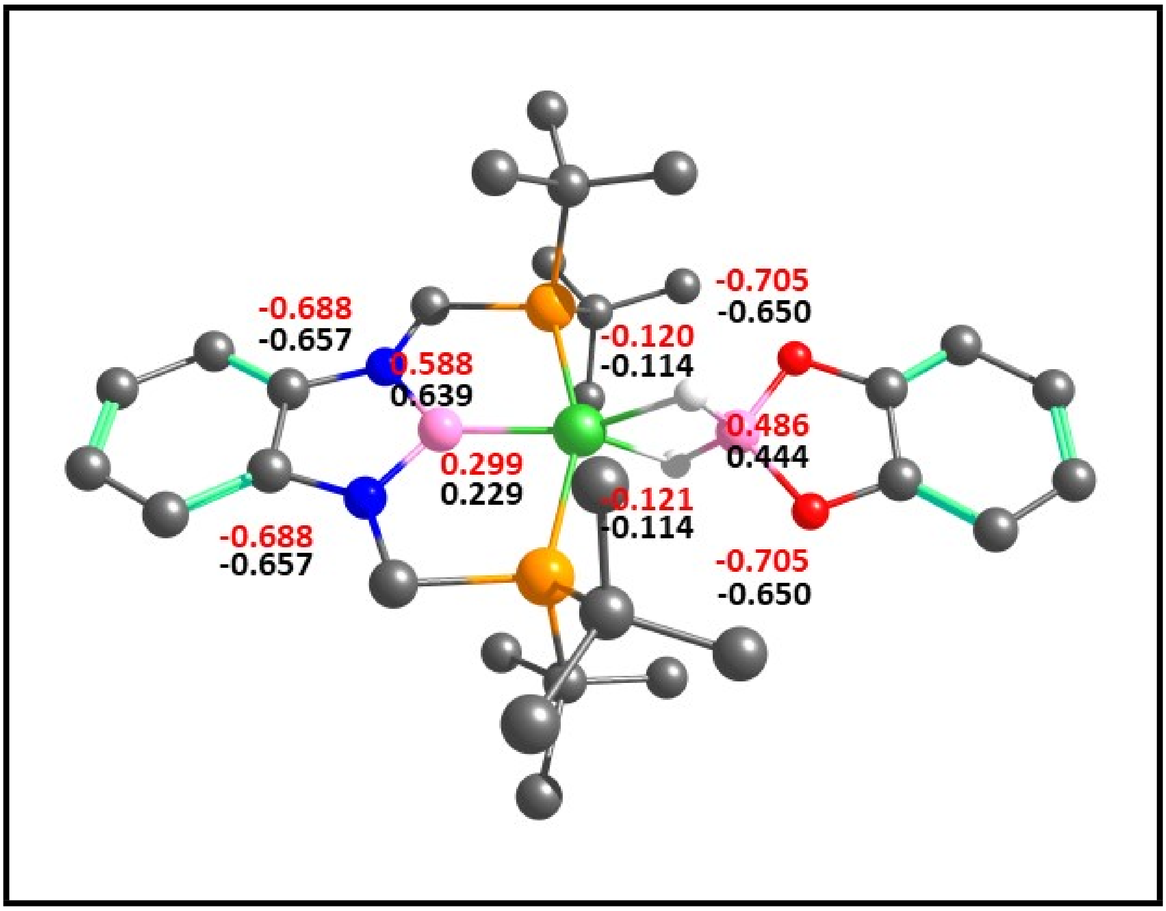

VIII– LOCALIZED MOLECULAR ORBITAL ANALYSIS

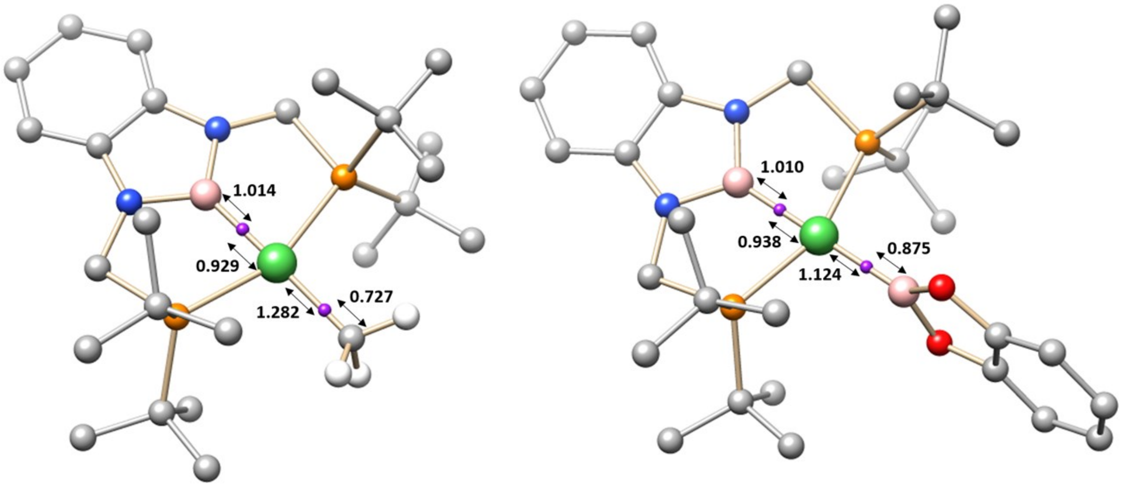

Figure S50. Comparison of the centroids of selected Localized Molecular Orbitals for complexes **1** and **3**. Most of the hydrogen atoms have been omitted for clarity. Selected distances in angstroms.

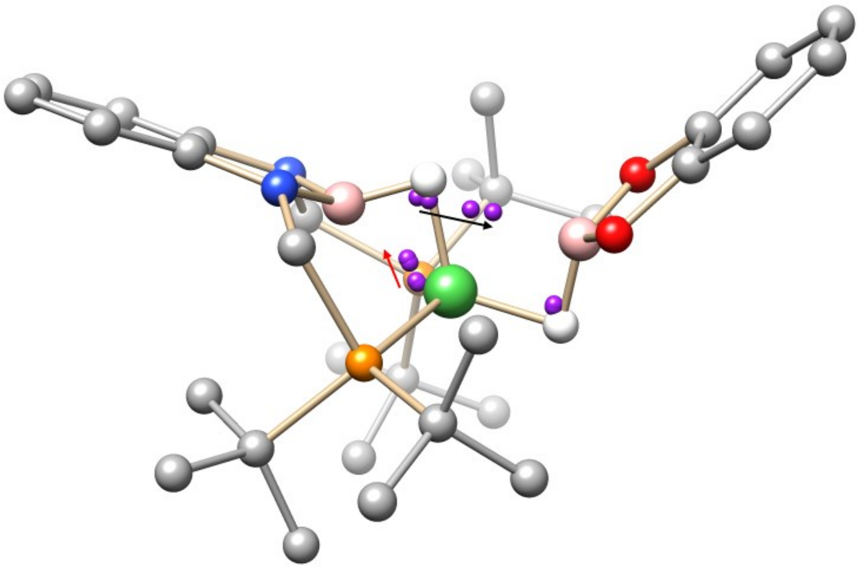

Figure S51. Depiction of the electron rearrangement that takes place during **TS2**. Arrows indicate the direction of the electron movement along the IRC. Most of the hydrogen atoms have been omitted for clarity.

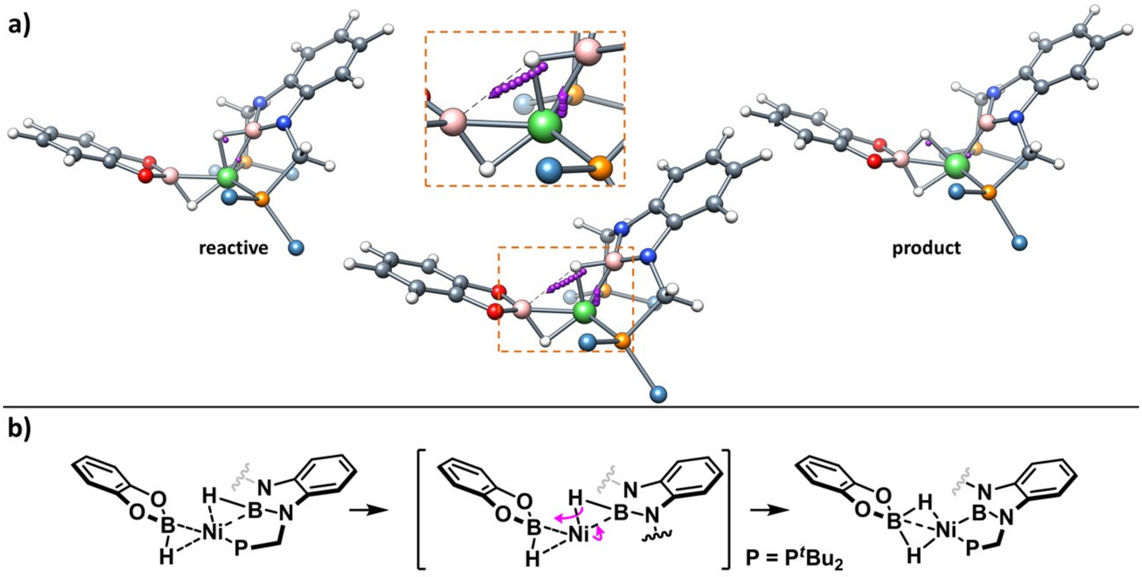

Figure 52. Superposition over reactant structures of the localized orbital centroids (purple dots) along the IRC for the **Int1'** to **4** interconversion through **TS2** and (b) the corresponding arrow-pushing schemes.

IX– COMPARISON OF THE REACTION WITH PBP AND CARBENE LIGANDS

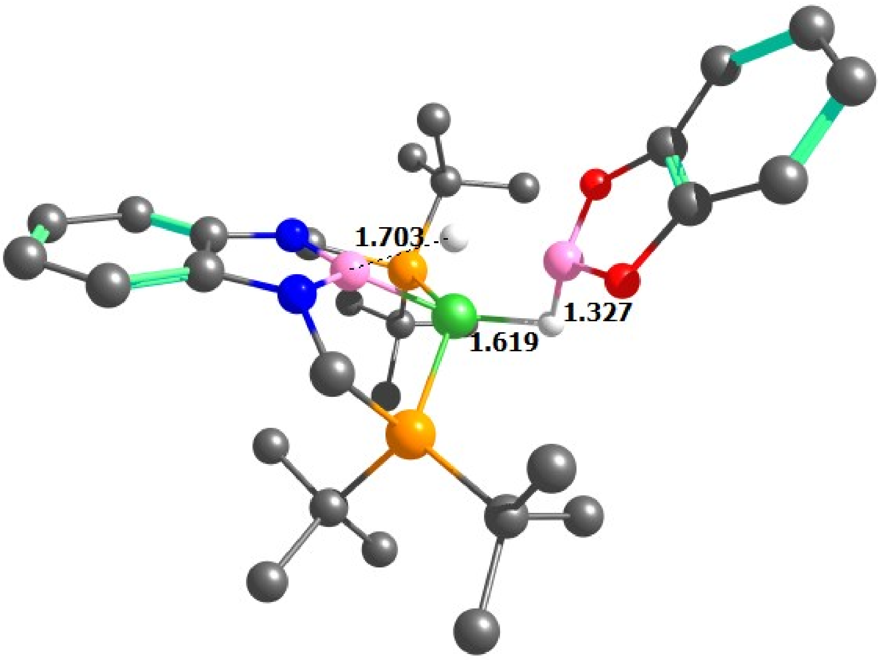

Figure S53. Optimized structure of **TS2**. Most of the hydrogen atoms have been omitted for clarity. Selected distances in angstroms.

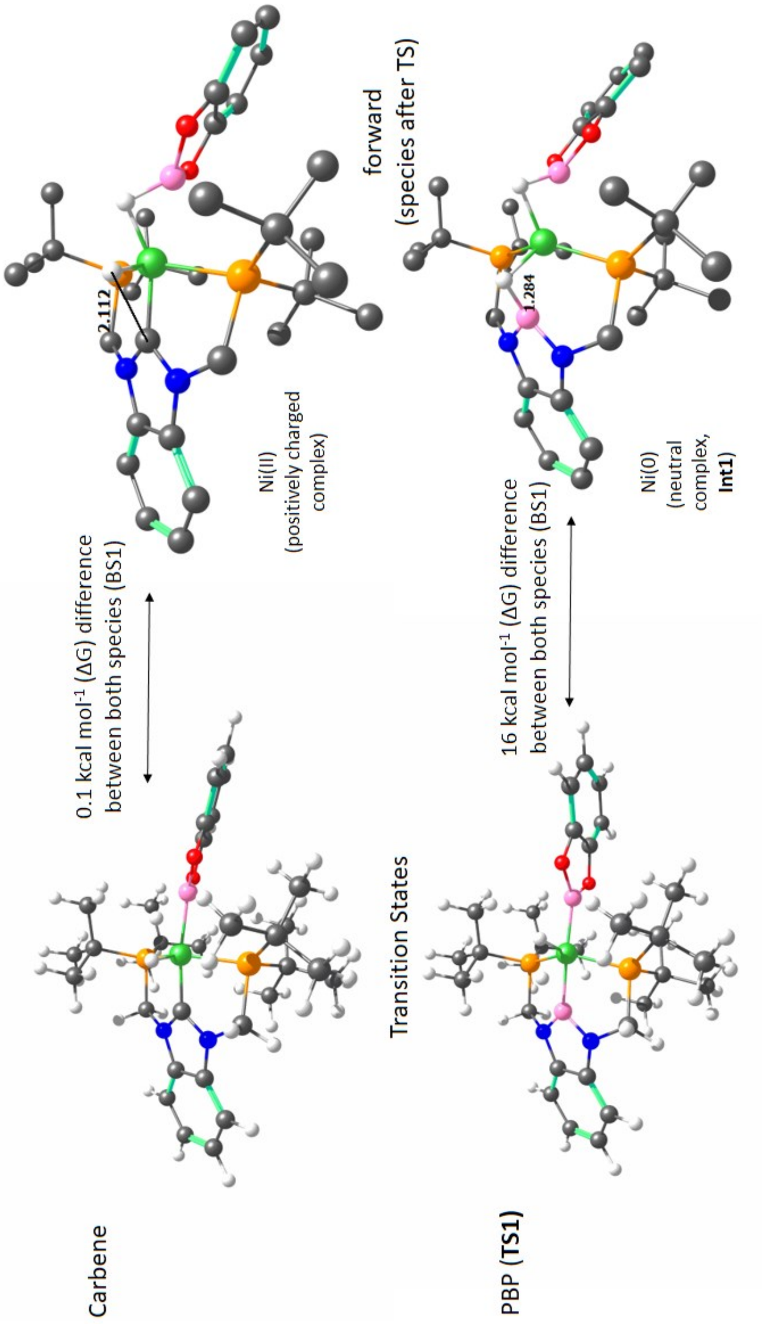

Figure 54. Geometry and Gibbs energy comparison between TS1-Int1 and their carbene analogue.

X– ENERGY PROFILE OF THE REACTION WITH UNCONSTRAINED PBP LIGANDS

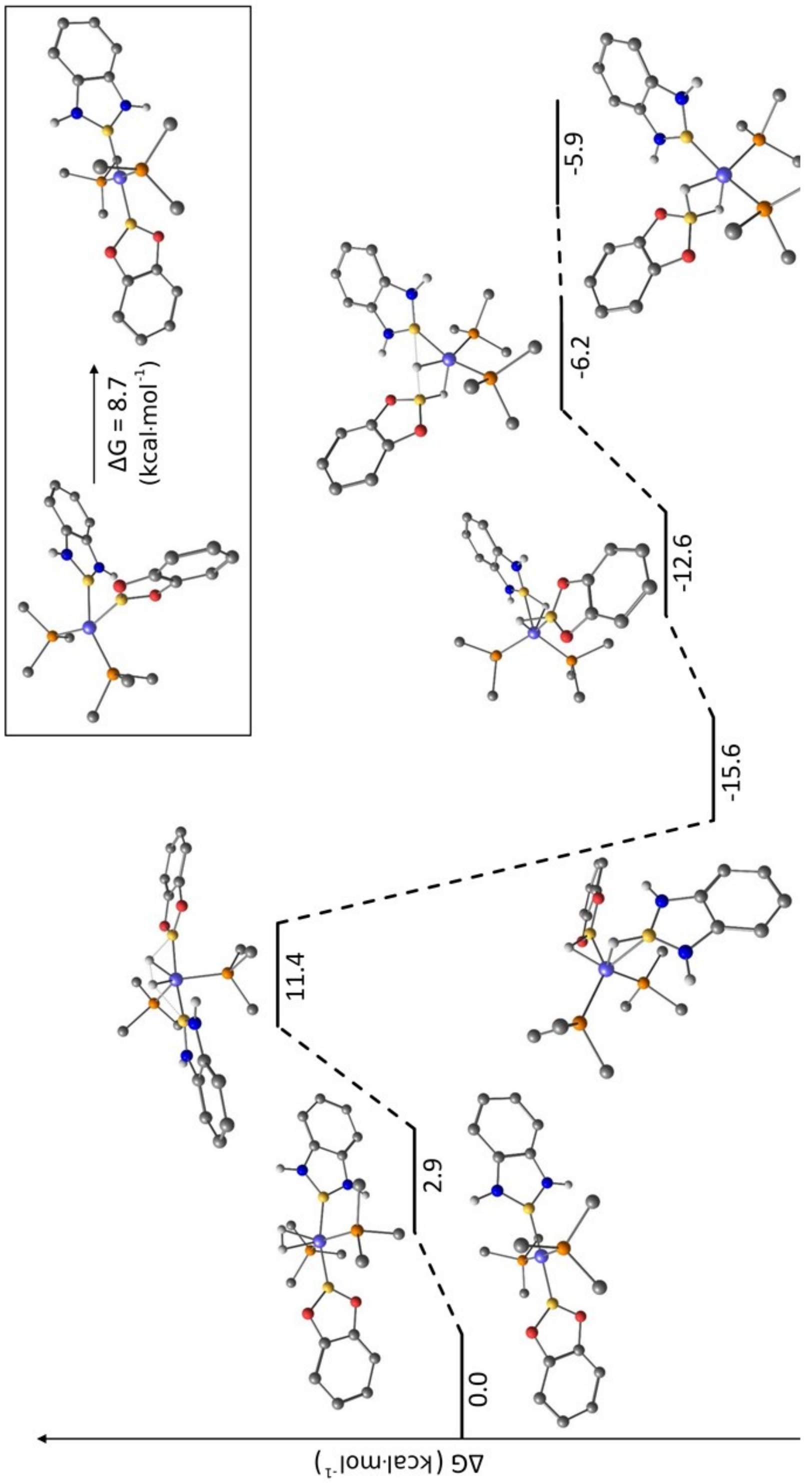

Figure S55. Gibbs Energy difference between the *cis*- and *trans*- boryl isomers of (PMe3)2(B(NH)2R)NiBcat (top). Gibbs Energy profile in toluene for the reaction of **3** (unconstrained) with H_2.

XI – SELECTED MOLECULAR ORBITALS OF 3

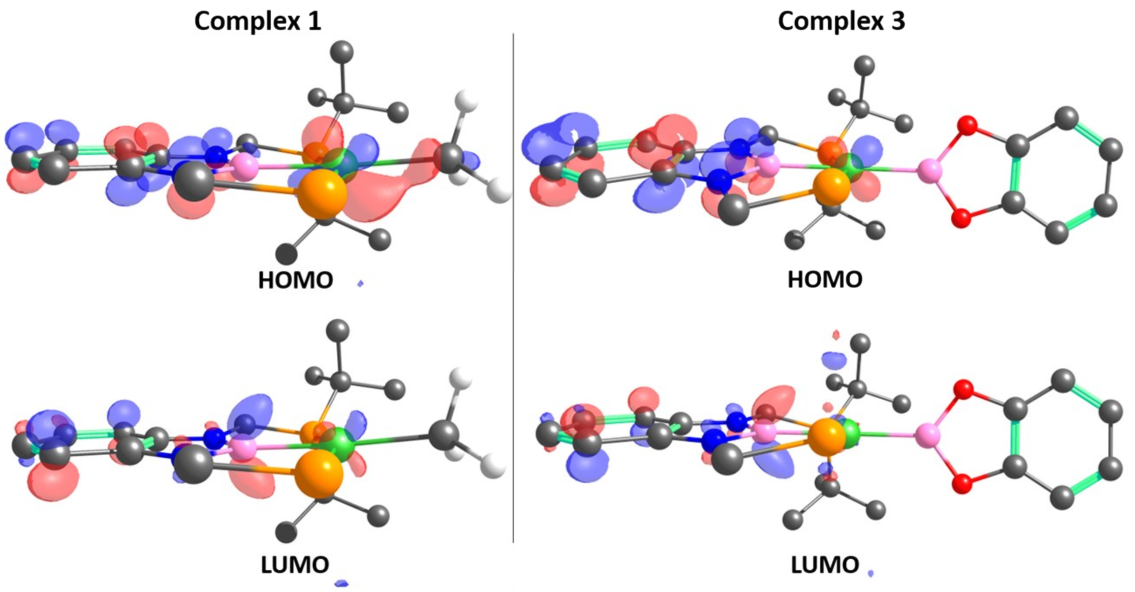

Figure S56. Frontier orbital comparison of complexes **1** and **3**. Some atoms have been omitted for clarity.

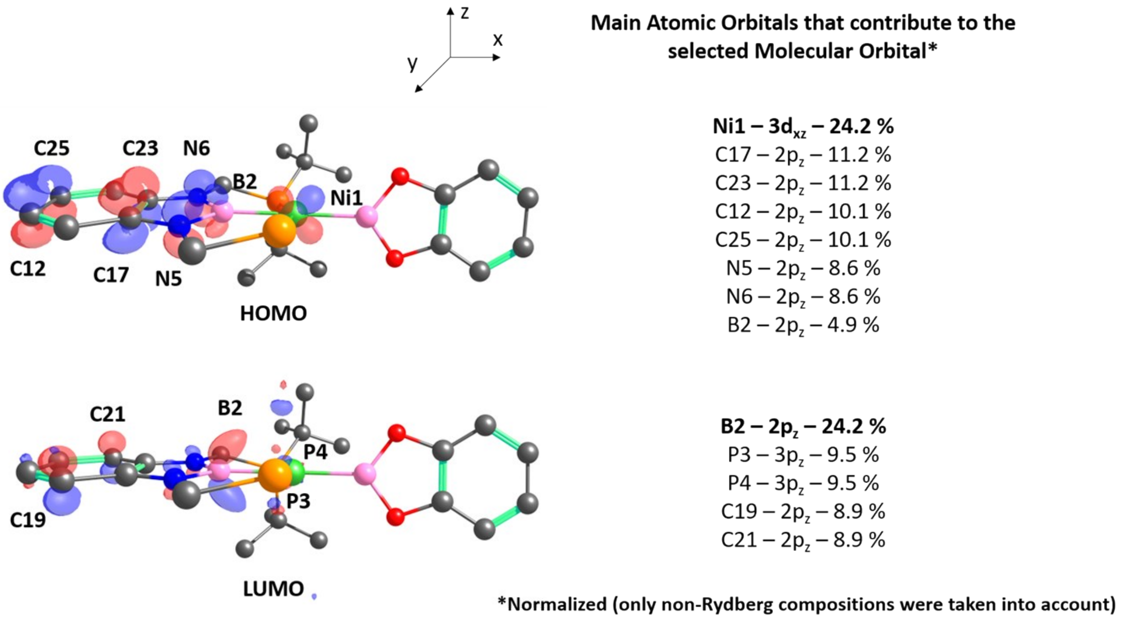

Figure S57. Frontier orbitals of **3** and atomic orbital composition. Some atoms have been omitted for clarity.

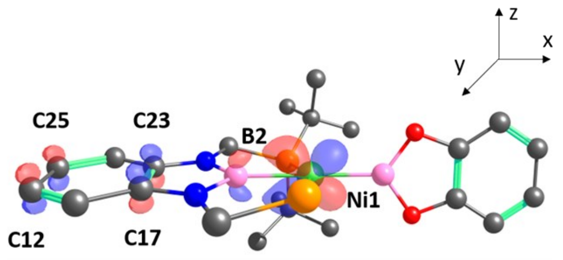

HOMO-6

Main Atomic Orbitals that contribute to the selected Molecular Orbital*

Ni1 – $3d_{xz}$ – 69.6 %
B2 – $2p_z$ – 7.8 %
C17 – $2p_z$ – 4.5 %
C23 – $2p_z$ – 4.5 %
C12 – $2p_z$ – 4.2 %
C25 – $2p_z$ – 4.2 %

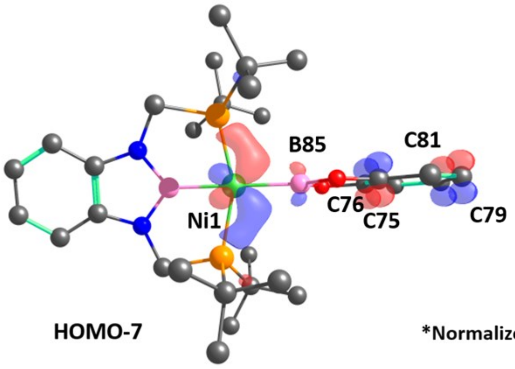

HOMO-7

Ni1 – $3d_{xy}$ – 30.7 %
B85 – $2p_y$ – 7.9 %
C79 – $2p_y$ – 7.2 %
C81 – $2p_y$ – 7.2 %
C75 – $2p_y$ – 7.2 %
C76 – $2p_y$ – 7.2 %
P3 – $3p_y$ – 7.1 %
P4 – $3p_y$ – 7.1 %

***Normalized (only non-Rydberg compositions were taken into account)**

Figure S58. Selected inner molecular orbitals of **3** and atomic orbital composition. Some atoms have been omitted for clarity.

XII – QTAIM ANALYSES

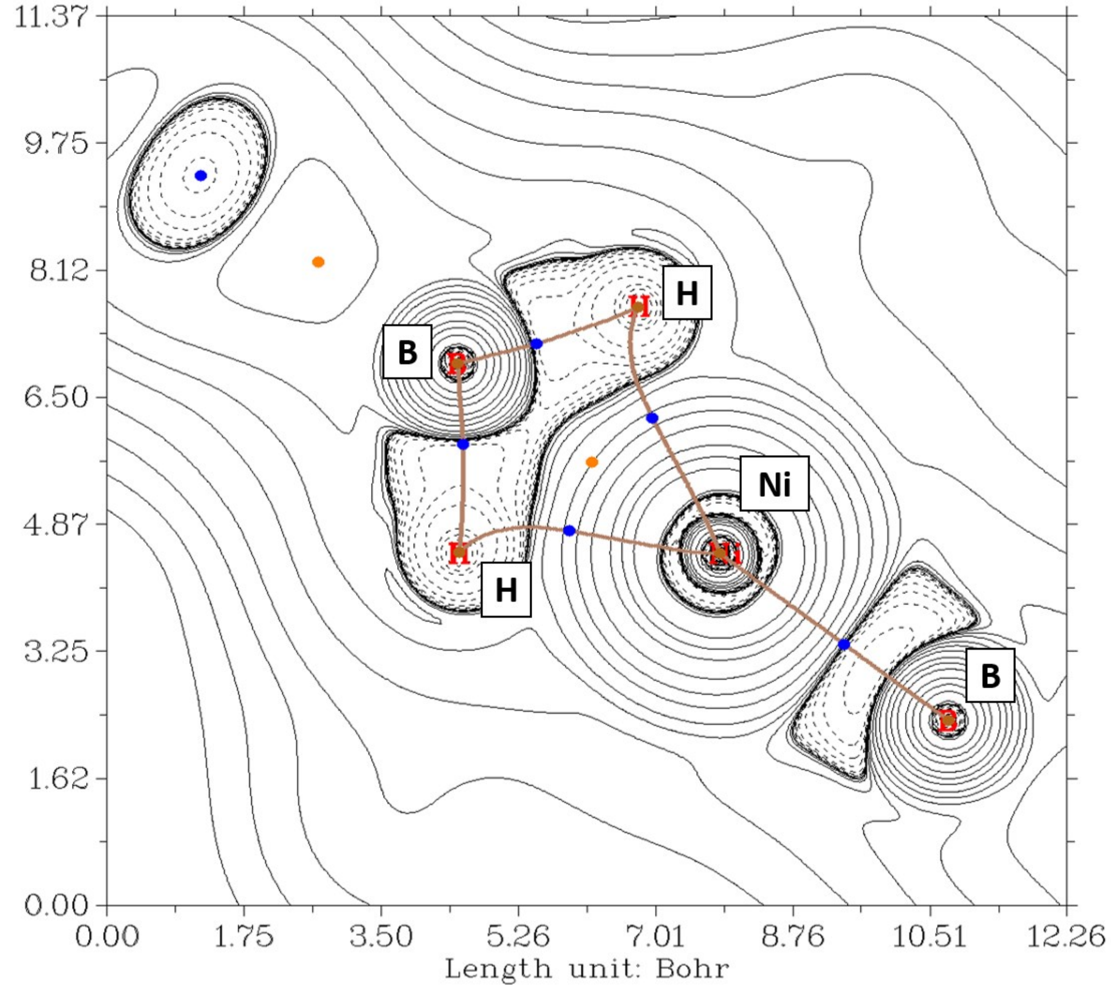

Figure S59. Fragment of the plot of the laplacian of the electron density ($\nabla^2\rho$) of complex **4**. Blue: bond critical point. Orange: ring critical point.

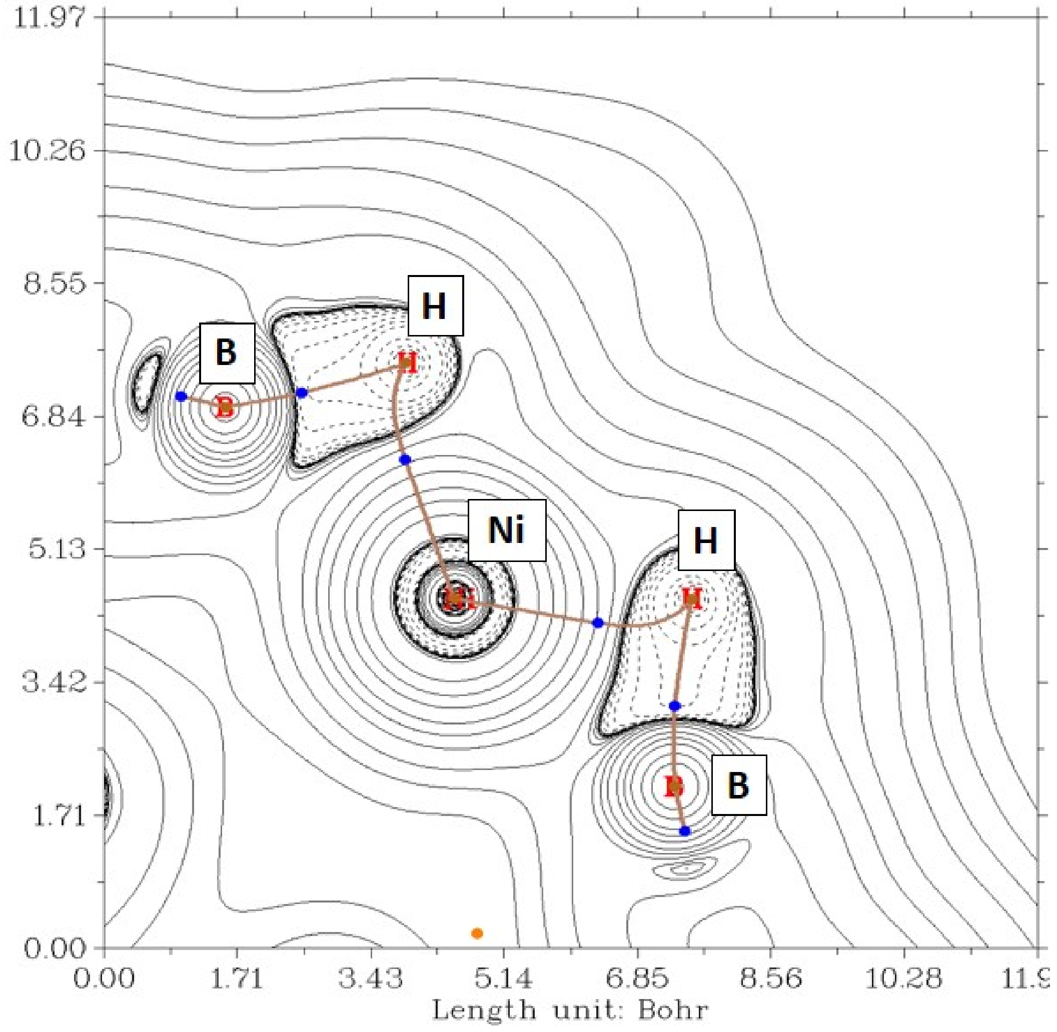

Figure S60. Fragment of the plot of the laplacian of the electron density ($\nabla^2\rho$) of complex **Int1**. Blue: bond critical point. Orange: ring critical point.

XIII – OPTIMIZED GEOMETRIES (ENERGY PROFILE SPECIES)

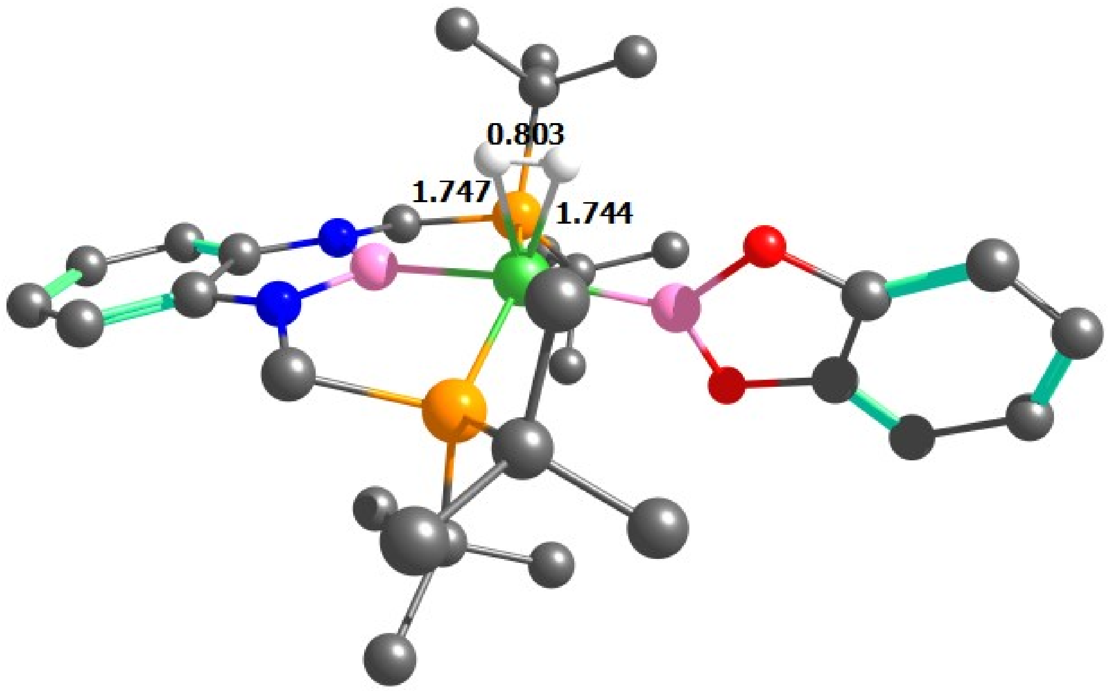

Figure S61. Optimized structure of **3·H$_2$**. Most of the hydrogen atoms have been omitted for clarity. Selected distances in angstroms.

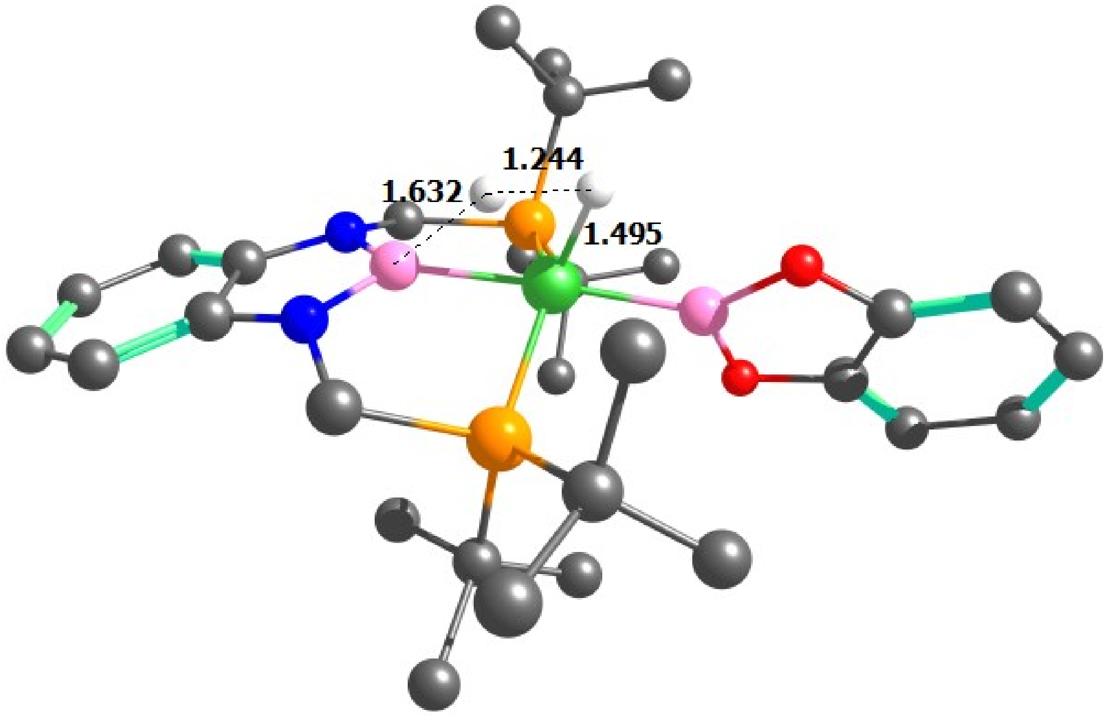

Figure S62. Optimized structure of **TS1**. Most of the hydrogen atoms have been omitted for clarity. Selected distances in angstroms.

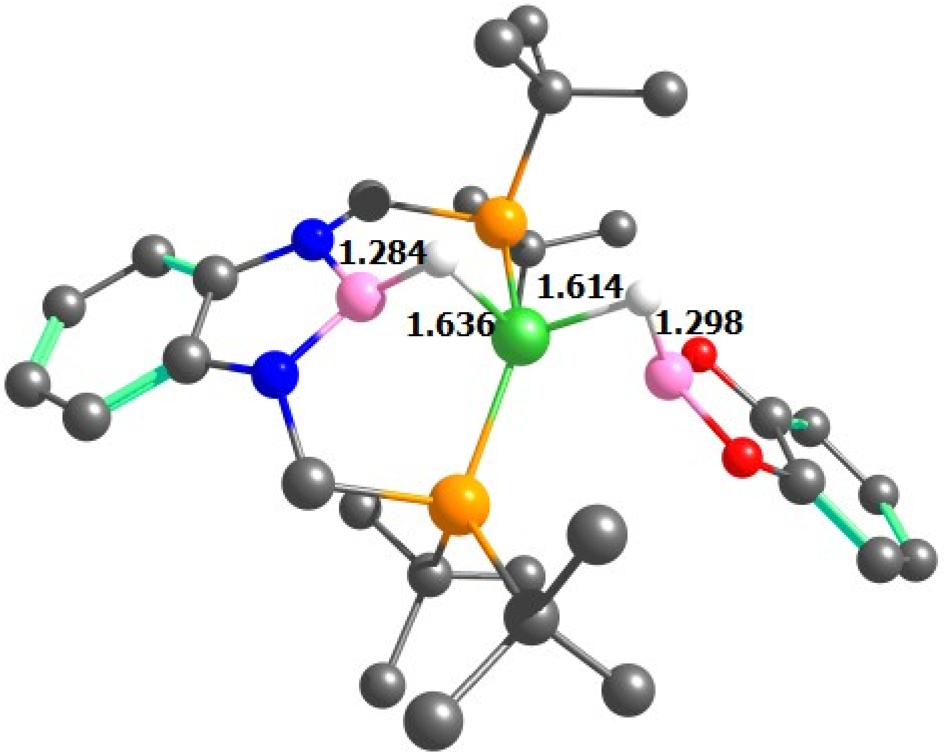

Figure S63. Optimized structure of **Int1**. Most of the hydrogen atoms have been omitted for clarity. Selected distances in angstroms.

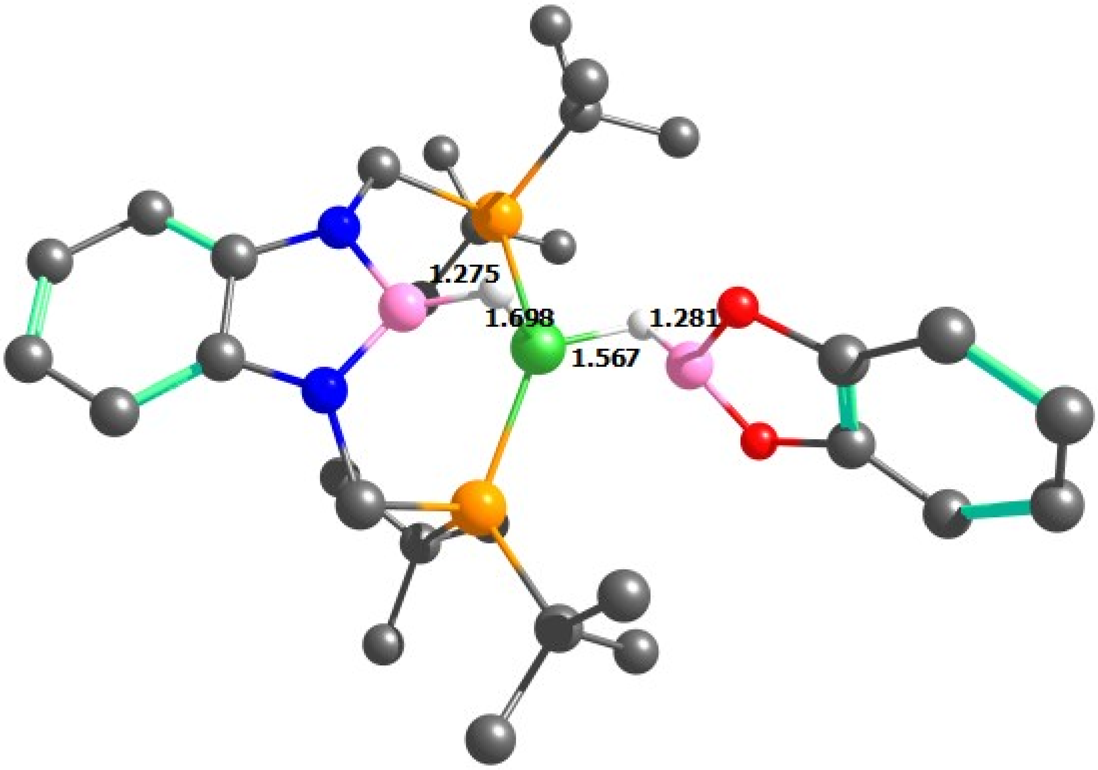

Figure S64. Optimized structure of **Int1'**. Most of the hydrogen atoms have been omitted for clarity. Selected distances in angstroms.

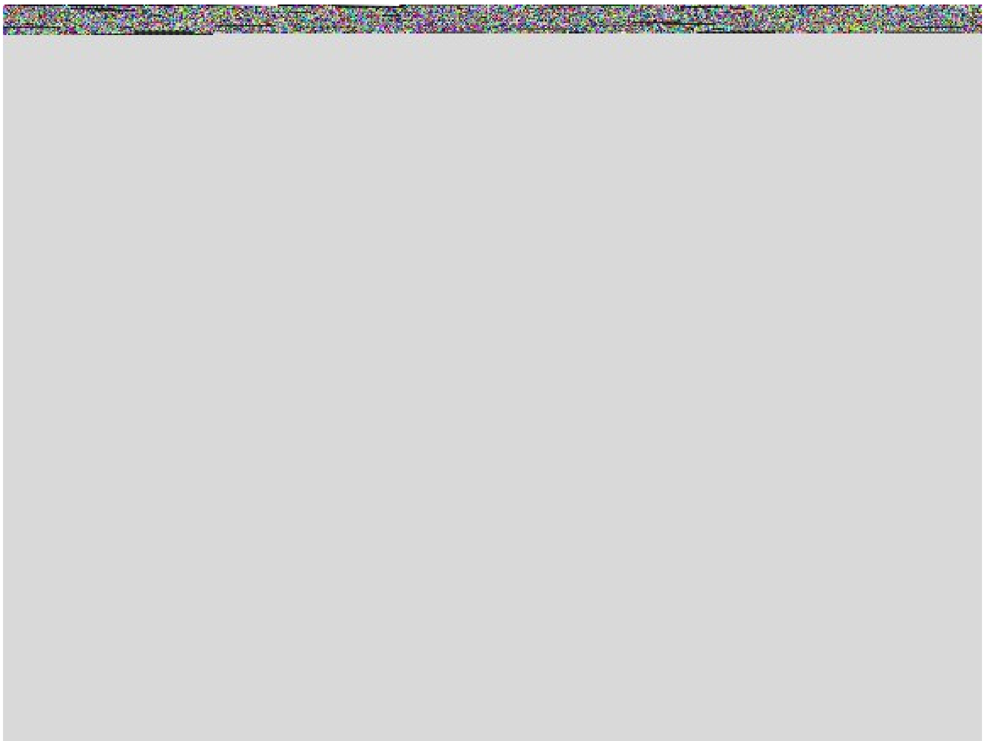

Figure S65. Optimized structure of **TS2**. Most of the hydrogen atoms have been omitted for clarity. Selected distances in angstroms.

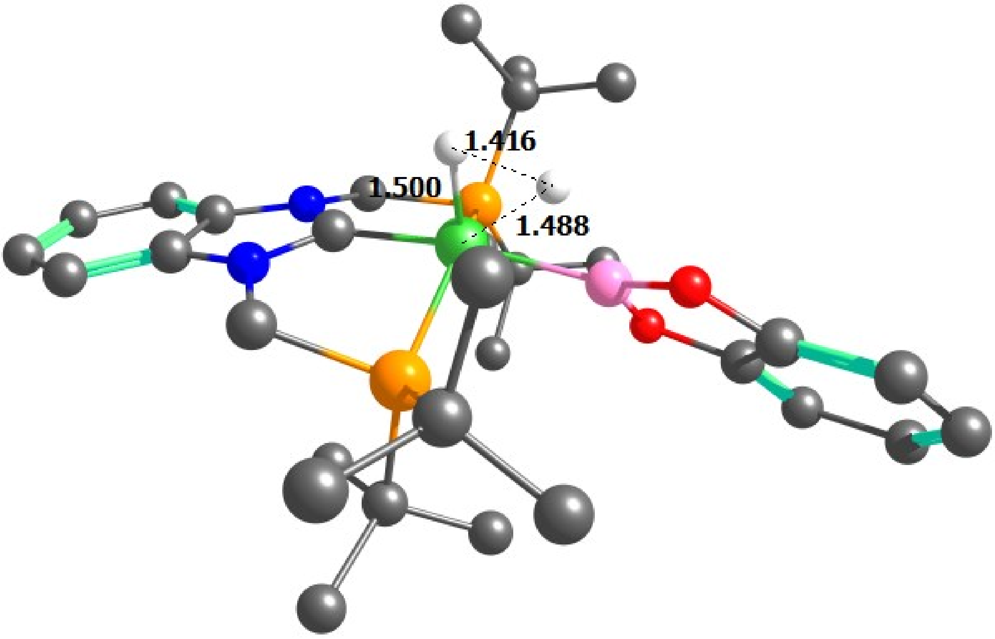

Figure S66. Optimized structure of **TS1** in which the boryl fragment of the PBP has been replaced by a carbene fragment. Most of the hydrogen atoms have been omitted for clarity. Selected distances in angstroms. The computed structure has an overall charge of +1.

XIV – CARTESIAN COORDINATES OF THE OPTIMIZED STRUCTURES

H_2

H	0.000000000	0.000000000	0.372139000
H	0.000000000	0.000000000	-0.372139000

Complex **3**

Ni	-0.056486000	-0.000023000	0.000618000
B	1.891630000	0.000332000	0.001154000
P	0.370840000	2.126807000	-0.057329000
P	0.371605000	-2.126754000	0.058033000
N	2.766103000	1.115422000	-0.224452000
N	2.766452000	-1.114552000	0.226352000
C	0.252954000	2.881408000	1.675558000
C	-0.509155000	3.168192000	-1.366359000
C	2.194812000	-2.402958000	0.496676000
H	2.295906000	-2.679650000	1.552841000
H	2.643877000	-3.204894000	-0.102863000
C	6.475010000	0.682964000	-0.147978000
H	7.419642000	1.207313000	-0.262399000
C	2.194043000	2.403467000	-0.495590000
H	2.295199000	2.679563000	-1.551908000
H	2.642672000	3.205947000	0.103551000
C	4.081748000	0.699522000	-0.146599000
C	0.254673000	-2.880867000	-1.675140000
C	5.278072000	1.389205000	-0.298273000
H	5.278935000	2.451030000	-0.529158000
C	5.278533000	-1.388175000	0.297042000
H	5.279758000	-2.450008000	0.527888000
C	4.081975000	-0.698532000	0.147040000
C	-0.508481000	-3.168787000	1.366503000
C	6.475238000	-0.681901000	0.145028000
H	7.420049000	-1.206220000	0.258101000
C	-1.116752000	-2.556529000	-2.275595000
H	-1.302327000	-1.480010000	-2.289219000
H	-1.150624000	-2.928045000	-3.307960000
H	-1.939283000	-3.024718000	-1.729764000
C	0.488769000	-4.391051000	-1.707601000
H	-0.316253000	-4.940682000	-1.212129000
H	0.514744000	-4.726993000	-2.751952000
H	1.440022000	-4.682346000	-1.250686000
C	1.320029000	-2.178426000	-2.529857000
H	1.165626000	-1.094269000	-2.544669000
H	2.340467000	-2.367342000	-2.183112000
H	1.247217000	-2.547431000	-3.560631000
C	-0.512923000	-2.268229000	2.610435000
H	-1.072722000	-1.346813000	2.420376000

H	-0.991715000	-2.796548000	3.444665000
H	0.500461000	-1.997294000	2.929324000
C	0.188949000	-4.491658000	1.699192000
H	-0.375650000	-4.997220000	2.492897000
H	0.231362000	-5.173908000	0.847547000
H	1.206609000	-4.351266000	2.074231000
C	-1.954460000	-3.452156000	0.956693000
H	-2.493771000	-3.874228000	1.813905000
H	-2.480280000	-2.542318000	0.657452000
H	-2.017167000	-4.179417000	0.142124000
C	-1.955349000	3.451268000	-0.957084000
H	-2.494568000	3.872686000	-1.814671000
H	-2.480964000	2.541435000	-0.657477000
H	-2.018504000	4.178949000	-0.142925000
C	-0.512907000	2.267399000	-2.610122000
H	0.500680000	1.996779000	-2.928635000
H	-1.072407000	1.345814000	-2.420064000
H	-0.991606000	2.795414000	-3.444591000
C	0.188052000	4.491178000	-1.699122000
H	-0.376566000	4.996552000	-2.492931000
H	0.230310000	5.173549000	-0.847568000
H	1.205756000	4.350874000	-2.074082000
C	-1.118606000	2.556704000	2.275518000
H	-1.303912000	1.480134000	2.289087000
H	-1.152950000	2.928229000	3.307867000
H	-1.941035000	3.024684000	1.729364000
C	1.318260000	2.179653000	2.530941000
H	1.245061000	2.549184000	3.561499000
H	1.164132000	1.095462000	2.546256000
H	2.338734000	2.368633000	2.184344000
C	0.486480000	4.391680000	1.707716000
H	-0.318451000	4.940891000	1.211635000
H	0.511739000	4.727920000	2.751988000
H	1.437901000	4.683160000	1.251265000
C	-4.196754000	-0.023714000	0.696213000
C	-4.196558000	0.022733000	-0.697406000
C	-5.371633000	-0.049339000	1.424123000
C	-5.371204000	0.048216000	-1.425685000
C	-6.568106000	-0.024819000	0.696310000
H	-5.360032000	-0.086534000	2.508989000
C	-6.567896000	0.023554000	-0.698240000
H	-5.359298000	0.085418000	-2.510549000
H	-7.513979000	-0.043439000	1.230174000
H	-7.513601000	0.042065000	-1.232404000
B	-2.055475000	-0.000315000	-0.000329000
O	-2.910070000	0.039626000	-1.141866000
O	-2.910404000	-0.040430000	1.141073000

Complex **3·H$_2$**

Ni	-0.082264000	0.051974000	-0.469022000
B	-2.043514000	0.031287000	-0.227865000
O	-2.791060000	0.014320000	0.982095000
O	-2.994770000	-0.000446000	-1.291079000
C	-4.112314000	-0.026057000	0.654472000
C	-5.215157000	-0.054640000	1.487127000
H	-5.104833000	-0.046596000	2.566928000
C	-6.471486000	-0.094599000	0.869582000
H	-7.365133000	-0.118367000	1.486610000
C	-6.596439000	-0.105084000	-0.520296000
H	-7.585891000	-0.136616000	-0.967246000
C	-5.471345000	-0.075607000	-1.353830000
H	-5.558155000	-0.083277000	-2.435778000
C	-4.236377000	-0.035387000	-0.733876000
B	1.863781000	-0.004218000	-0.464791000
N	2.688706000	-1.162379000	-0.669623000
N	2.769734000	1.045497000	-0.098407000
C	4.007207000	-0.839967000	-0.399145000
C	5.163270000	-1.609548000	-0.409202000
H	5.126544000	-2.659599000	-0.686621000
C	6.371439000	-0.998133000	-0.057357000
H	7.285405000	-1.585493000	-0.061316000
C	6.420477000	0.350289000	0.295938000
H	7.371858000	0.800881000	0.564483000
C	5.262274000	1.135069000	0.311469000
H	5.299661000	2.183783000	0.593694000
C	4.057520000	0.539182000	-0.035036000
P	0.392437000	2.137131000	0.028663000
C	2.234637000	2.265486000	0.436784000
H	2.687715000	3.167279000	0.007216000
H	2.374015000	2.310166000	1.524271000
C	-0.393589000	2.935847000	1.560794000
C	-0.399066000	1.829530000	2.621846000
H	0.607380000	1.452684000	2.836186000
H	-1.022622000	0.992953000	2.302312000
H	-0.808550000	2.226723000	3.559560000
C	0.367769000	4.143287000	2.120719000
H	-0.147370000	4.486563000	3.026827000
H	0.404437000	4.987843000	1.431815000
H	1.392272000	3.897775000	2.414915000
C	-1.840367000	3.341867000	1.270401000
H	-2.404144000	2.534384000	0.796948000
H	-1.901188000	4.231262000	0.636988000
H	-2.343587000	3.579893000	2.215798000
C	0.246039000	3.295874000	-1.473324000

C	0.491779000	4.770894000	-1.152078000
H	1.452622000	4.938296000	-0.654851000
H	-0.300212000	5.197898000	-0.532242000
H	0.509526000	5.339889000	-2.090232000
C	1.296955000	2.847456000	-2.499453000
H	1.164731000	3.432788000	-3.417848000
H	1.196504000	1.791602000	-2.758628000
H	2.321694000	3.010819000	-2.152250000
C	-1.144720000	3.140710000	-2.099410000
H	-1.942589000	3.478823000	-1.434061000
H	-1.356989000	2.103251000	-2.367856000
H	-1.197453000	3.747208000	-3.012487000
P	0.309978000	-2.083197000	-0.129059000
C	2.049040000	-2.445883000	-0.778761000
H	2.554672000	-3.212184000	-0.178993000
H	2.004310000	-2.803764000	-1.814917000
C	0.476778000	-2.547454000	1.705163000
C	1.654189000	-1.740259000	2.269827000
H	1.706130000	-1.906252000	3.353159000
H	1.521621000	-0.667790000	2.097868000
H	2.617483000	-2.037597000	1.845031000
C	0.735587000	-4.033175000	1.958387000
H	0.938105000	-4.179712000	3.026935000
H	1.605641000	-4.412748000	1.412287000
H	-0.129953000	-4.652408000	1.709302000
C	-0.788966000	-2.113063000	2.447654000
H	-1.674433000	-2.675394000	2.144586000
H	-0.998408000	-1.056214000	2.280652000
H	-0.645319000	-2.274887000	3.523624000
C	-0.741649000	-3.364399000	-1.056156000
C	-0.991666000	-2.767157000	-2.447295000
H	-0.061325000	-2.519354000	-2.971098000
H	-1.602104000	-1.863120000	-2.384164000
H	-1.530666000	-3.500397000	-3.060332000
C	-0.055873000	-4.724954000	-1.227878000
H	-0.716197000	-5.379608000	-1.810988000
H	0.142549000	-5.229588000	-0.281289000
H	0.885977000	-4.652413000	-1.779538000
C	-2.094334000	-3.556897000	-0.368110000
H	-2.753991000	-4.135564000	-1.026608000
H	-2.591602000	-2.603803000	-0.165121000
H	-2.007327000	-4.107815000	0.571986000
H	-0.582231000	0.105431000	-2.138533000
H	0.215118000	0.030545000	-2.190783000

TS1

Ni	-0.096939000	0.042174000	-0.637292000
B	-2.013159000	0.002500000	-0.396607000
O	-2.667518000	0.148559000	0.857887000
O	-3.025752000	-0.207819000	-1.368599000
C	-4.006605000	0.020447000	0.637596000
C	-5.047969000	0.083972000	1.543756000
H	-4.865962000	0.255840000	2.599882000
C	-6.340028000	-0.087031000	1.031435000
H	-7.187977000	-0.045920000	1.708905000
C	-6.557983000	-0.308590000	-0.329220000
H	-7.573134000	-0.437754000	-0.693221000
C	-5.494268000	-0.368878000	-1.237814000
H	-5.652732000	-0.540109000	-2.297760000
C	-4.223700000	-0.197612000	-0.721447000
B	1.855195000	0.024455000	-0.792708000
N	2.681058000	-1.151485000	-0.871919000
N	2.740346000	1.106468000	-0.444845000
C	3.974351000	-0.815222000	-0.509776000
C	5.110726000	-1.596589000	-0.349903000
H	5.084890000	-2.662317000	-0.559849000
C	6.285152000	-0.977117000	0.093122000
H	7.183299000	-1.573940000	0.225252000
C	6.318994000	0.389108000	0.366608000
H	7.242102000	0.845989000	0.711843000
C	5.178798000	1.184988000	0.204313000
H	5.203159000	2.248813000	0.424223000
C	4.010858000	0.581512000	-0.238180000
P	0.340628000	2.148480000	0.006056000
C	2.211092000	2.295363000	0.171804000
H	2.590742000	3.224072000	-0.271232000
H	2.470076000	2.307678000	1.238376000
C	-0.252982000	2.912462000	1.643508000
C	-0.144169000	1.786322000	2.676258000
H	0.878843000	1.407116000	2.772165000
H	-0.799593000	0.954473000	2.412860000
H	-0.446720000	2.165384000	3.660850000
C	0.582714000	4.100995000	2.134097000
H	0.178511000	4.433710000	3.098745000
H	0.554862000	4.958150000	1.460406000
H	1.630378000	3.838034000	2.307372000
C	-1.721821000	3.332615000	1.543447000
H	-2.351505000	2.520405000	1.172322000
H	-1.862075000	4.210614000	0.907431000
H	-2.084141000	3.597254000	2.544969000
C	-0.033348000	3.340709000	-1.433164000
C	0.229406000	4.813065000	-1.110807000

H	1.254011000	4.992801000	-0.768682000
H	-0.462045000	5.208870000	-0.363022000
H	0.086892000	5.405296000	-2.023694000
C	0.867527000	2.940709000	-2.610595000
H	0.659659000	3.611780000	-3.453265000
H	0.667968000	1.920063000	-2.943117000
H	1.934222000	3.023733000	-2.380981000
C	-1.491020000	3.163928000	-1.873025000
H	-2.204961000	3.445328000	-1.095065000
H	-1.694013000	2.129043000	-2.160435000
H	-1.682266000	3.804337000	-2.743390000
P	0.336686000	-2.087218000	-0.097633000
C	2.044553000	-2.442342000	-0.821197000
H	2.607683000	-3.143929000	-0.195040000
H	1.942103000	-2.889908000	-1.816320000
C	0.580159000	-2.465506000	1.743412000
C	1.753378000	-1.584160000	2.195519000
H	1.834738000	-1.629636000	3.288431000
H	1.602296000	-0.538993000	1.909567000
H	2.711483000	-1.914300000	1.781757000
C	0.900428000	-3.921631000	2.080727000
H	1.162701000	-3.991627000	3.144180000
H	1.752336000	-4.310924000	1.513150000
H	0.043112000	-4.580687000	1.918494000
C	-0.678771000	-2.029507000	2.498039000
H	-1.524775000	-2.696940000	2.318025000
H	-0.988282000	-1.023909000	2.209585000
H	-0.477299000	-2.036136000	3.576622000
C	-0.721820000	-3.421180000	-0.933819000
C	-0.993611000	-2.916728000	-2.357737000
H	-0.071083000	-2.728777000	-2.918338000
H	-1.577516000	-1.994011000	-2.351864000
H	-1.560559000	-3.682145000	-2.902893000
C	-0.033871000	-4.788683000	-1.027383000
H	-0.703644000	-5.482550000	-1.551421000
H	0.185557000	-5.226594000	-0.052730000
H	0.896631000	-4.752776000	-1.601867000
C	-2.060626000	-3.578849000	-0.209411000
H	-2.731808000	-4.192601000	-0.822817000
H	-2.560152000	-2.619695000	-0.044374000
H	-1.950861000	-4.081256000	0.755530000
H	-0.557280000	0.082442000	-2.059171000
H	0.679596000	0.061406000	-1.923912000

TS1 (carbene instead of boryl)

Ni	0.045284000	0.050945000	-0.709682000
B	-1.964542000	0.123998000	-0.607416000
O	-2.517446000	0.869781000	0.435787000
O	-2.980718000	-0.558168000	-1.280799000
C	-3.863688000	0.595923000	0.432449000
C	-4.842876000	1.054761000	1.291015000
H	-4.611832000	1.727766000	2.109946000
C	-6.142971000	0.601062000	1.044268000
H	-6.949515000	0.934145000	1.690148000
C	-6.426250000	-0.268695000	-0.011387000
H	-7.448576000	-0.597473000	-0.170027000
C	-5.423115000	-0.728223000	-0.871288000
H	-5.631490000	-1.403875000	-1.694010000
C	-4.145231000	-0.271249000	-0.616386000
N	2.508125000	-1.335764000	-0.554912000
N	2.779498000	0.799875000	-0.266528000
C	3.853228000	-1.160734000	-0.265995000
C	4.908625000	-2.054377000	-0.132369000
H	4.775420000	-3.119620000	-0.290036000
C	6.147540000	-1.514835000	0.201882000
H	6.999821000	-2.177719000	0.312464000
C	6.322086000	-0.137265000	0.394273000
H	7.306233000	0.241251000	0.651626000
C	5.264402000	0.758041000	0.259633000
H	5.399554000	1.824461000	0.407710000
C	4.028476000	0.219856000	-0.074146000
P	0.505166000	2.231793000	-0.058043000
C	2.368362000	2.128693000	0.127993000
H	2.880653000	2.892168000	-0.463716000
H	2.641688000	2.270347000	1.177214000
C	0.002746000	3.116442000	1.548344000
C	-0.022035000	2.020064000	2.619174000
H	0.956512000	1.542985000	2.745489000
H	-0.758425000	1.247219000	2.387009000
H	-0.289923000	2.466002000	3.584562000
C	0.969700000	4.217761000	2.003692000
H	0.586357000	4.636724000	2.941917000
H	1.052706000	5.041605000	1.294269000
H	1.975701000	3.846859000	2.220033000
C	-1.398292000	3.721849000	1.417133000
H	-2.129767000	2.992890000	1.066832000
H	-1.413401000	4.587844000	0.750835000
H	-1.719763000	4.069718000	2.406165000
C	0.301531000	3.369374000	-1.562321000
C	0.736438000	4.811874000	-1.294614000
H	1.761492000	4.878920000	-0.916118000

H	0.073630000	5.326074000	-0.595179000
H	0.703903000	5.367664000	-2.239300000
C	1.165060000	2.797896000	-2.695808000
H	0.990958000	3.394232000	-3.598991000
H	0.907435000	1.760098000	-2.921623000
H	2.237237000	2.846984000	-2.480163000
C	-1.163028000	3.348264000	-2.018151000
H	-1.861652000	3.648622000	-1.234554000
H	-1.459212000	2.360984000	-2.384047000
H	-1.281936000	4.048210000	-2.853442000
P	0.004957000	-2.145931000	-0.051138000
C	1.741556000	-2.556576000	-0.637137000
H	2.211540000	-3.331252000	-0.023947000
H	1.695172000	-2.905049000	-1.672917000
C	0.053137000	-2.439428000	1.810851000
C	1.228359000	-1.612129000	2.347763000
H	1.195454000	-1.618313000	3.443343000
H	1.171825000	-0.569601000	2.019857000
H	2.199564000	-2.019769000	2.049206000
C	0.239733000	-3.895326000	2.237010000
H	0.360616000	-3.930657000	3.326405000
H	1.135892000	-4.347792000	1.799702000
H	-0.623040000	-4.518118000	1.988138000
C	-1.247404000	-1.878108000	2.397523000
H	-2.128497000	-2.450688000	2.097475000
H	-1.402242000	-0.837126000	2.099661000
H	-1.191138000	-1.911127000	3.491889000
C	-0.987302000	-3.473653000	-0.961711000
C	-1.141148000	-2.993781000	-2.411640000
H	-0.176415000	-2.819616000	-2.899140000
H	-1.730948000	-2.079007000	-2.479867000
H	-1.658893000	-3.773532000	-2.983089000
C	-0.277568000	-4.835370000	-0.973495000
H	-0.923004000	-5.547614000	-1.501409000
H	-0.101048000	-5.239661000	0.024157000
H	0.673398000	-4.815387000	-1.513515000
C	-2.369538000	-3.643167000	-0.326265000
H	-2.973222000	-4.294219000	-0.969283000
H	-2.906232000	-2.698684000	-0.226512000
H	-2.311825000	-4.118446000	0.655938000
H	-1.029717000	0.341426000	-1.696846000
H	0.206592000	-0.142201000	-2.188874000
C	1.867337000	-0.149728000	-0.561842000

Int1

Ni	-0.084485000	0.043339000	-0.881415000
B	-2.054700000	0.184355000	-0.754598000
O	-2.627287000	1.190383000	0.073358000
O	-3.055748000	-0.799572000	-0.981371000
C	-3.866754000	0.753509000	0.424447000
C	-4.781126000	1.336221000	1.280947000
H	-4.566804000	2.275493000	1.780754000
C	-5.989223000	0.653163000	1.473795000
H	-6.735303000	1.076497000	2.139923000
C	-6.250307000	-0.556505000	0.830668000
H	-7.196822000	-1.060525000	1.002873000
C	-5.314553000	-1.139810000	-0.033819000
H	-5.507976000	-2.080376000	-0.539647000
C	-4.127253000	-0.457053000	-0.217260000
B	1.941460000	-0.131000000	-0.997099000
N	2.615690000	-1.399874000	-0.832851000
N	2.840073000	0.862139000	-0.448949000
C	3.805175000	-1.190029000	-0.159796000
C	4.760768000	-2.084134000	0.305466000
H	4.660299000	-3.150389000	0.122393000
C	5.856159000	-1.576946000	1.015131000
H	6.614595000	-2.263052000	1.381536000
C	5.987702000	-0.210780000	1.254346000
H	6.846465000	0.159725000	1.806869000
C	5.027604000	0.696514000	0.789261000
H	5.127300000	1.761834000	0.978366000
C	3.943212000	0.202859000	0.078663000
P	0.518275000	2.216587000	-0.184603000
C	2.377410000	2.126977000	0.058757000
H	2.886357000	2.985592000	-0.395068000
H	2.577983000	2.165450000	1.135394000
C	-0.017337000	3.125890000	1.412246000
C	-0.230532000	2.015240000	2.446327000
H	0.679286000	1.429554000	2.615794000
H	-1.023640000	1.333637000	2.129922000
H	-0.519257000	2.459604000	3.407695000
C	1.019238000	4.114205000	1.964672000
H	0.596828000	4.596330000	2.855596000
H	1.275995000	4.906907000	1.258883000
H	1.944732000	3.627541000	2.284907000
C	-1.339560000	3.876125000	1.222726000
H	-2.103276000	3.246335000	0.765815000
H	-1.218665000	4.780891000	0.621208000
H	-1.706544000	4.192115000	2.207973000
C	0.383199000	3.424085000	-1.655821000
C	0.932067000	4.823514000	-1.370962000

H	1.975280000	4.807924000	-1.038889000
H	0.342646000	5.361322000	-0.624815000
H	0.899209000	5.415018000	-2.295026000
C	1.188083000	2.808851000	-2.809540000
H	1.154032000	3.491328000	-3.668007000
H	0.764174000	1.851835000	-3.125058000
H	2.241981000	2.645980000	-2.560657000
C	-1.074851000	3.532676000	-2.116891000
H	-1.721053000	4.013133000	-1.380308000
H	-1.496117000	2.550942000	-2.348948000
H	-1.114409000	4.135329000	-3.033377000
P	0.098686000	-2.114434000	-0.285320000
C	1.833214000	-2.606251000	-0.838246000
H	2.263071000	-3.367142000	-0.178262000
H	1.771890000	-3.034093000	-1.844506000
C	0.131039000	-2.376408000	1.589721000
C	1.292242000	-1.523540000	2.114591000
H	1.231435000	-1.462098000	3.208211000
H	1.248200000	-0.507037000	1.711199000
H	2.268719000	-1.947778000	1.863074000
C	0.338035000	-3.818400000	2.051897000
H	0.440618000	-3.831445000	3.144563000
H	1.252388000	-4.259964000	1.641776000
H	-0.503785000	-4.468012000	1.800082000
C	-1.177696000	-1.822039000	2.162267000
H	-2.051648000	-2.410927000	1.873119000
H	-1.338797000	-0.791113000	1.834024000
H	-1.125385000	-1.823350000	3.258313000
C	-0.908094000	-3.501595000	-1.121410000
C	-1.165149000	-3.007231000	-2.550916000
H	-0.234627000	-2.755902000	-3.073409000
H	-1.804559000	-2.121959000	-2.556984000
H	-1.664750000	-3.797533000	-3.126091000
C	-0.147780000	-4.834992000	-1.189168000
H	-0.814143000	-5.591282000	-1.623207000
H	0.158754000	-5.201014000	-0.206818000
H	0.736628000	-4.792335000	-1.829691000
C	-2.246246000	-3.765829000	-0.425852000
H	-2.842984000	-4.436832000	-1.057326000
H	-2.822746000	-2.854613000	-0.277935000
H	-2.115777000	-4.264251000	0.538351000
H	-1.341616000	0.466476000	-1.801782000
H	1.130992000	0.051597000	-1.976633000

Int1'

Ni	-0.236914000	0.323647000	0.127076000
B	1.127052000	-0.255907000	-1.294586000
N	2.401565000	0.433779000	-1.446706000
N	1.431106000	-1.658168000	-1.536637000
C	3.389404000	-0.509094000	-1.689739000
C	4.762662000	-0.361204000	-1.826608000
H	5.221436000	0.622999000	-1.786336000
C	5.541771000	-1.508846000	-2.025093000
H	6.617427000	-1.408343000	-2.139814000
C	4.954781000	-2.770220000	-2.082324000
H	5.575519000	-3.647353000	-2.242554000
C	3.569691000	-2.927946000	-1.944135000
H	3.114172000	-3.913291000	-1.991682000
C	2.790856000	-1.795563000	-1.746522000
P	-0.321811000	-1.925811000	0.463614000
C	0.562167000	-2.680185000	-1.018049000
H	1.127540000	-3.572809000	-0.728843000
H	-0.186545000	-2.989894000	-1.755263000
C	-1.937022000	-2.932549000	0.408489000
C	-2.718264000	-2.437419000	-0.818432000
H	-2.287179000	-2.811479000	-1.752678000
H	-2.746774000	-1.351608000	-0.896512000
H	-3.751879000	-2.799565000	-0.767468000
C	-1.746854000	-4.445428000	0.226462000
H	-2.731922000	-4.898050000	0.052700000
H	-1.320741000	-4.934410000	1.102278000
H	-1.126871000	-4.694276000	-0.640571000
C	-2.741168000	-2.683287000	1.686830000
H	-2.870628000	-1.618281000	1.890917000
H	-2.266976000	-3.152429000	2.554286000
H	-3.739557000	-3.127808000	1.583647000
C	0.726074000	-2.465411000	1.950862000
C	0.699694000	-3.946221000	2.322688000
H	0.949524000	-4.594724000	1.476466000
H	-0.265850000	-4.257158000	2.730016000
H	1.452027000	-4.129898000	3.101002000
C	2.167884000	-2.088636000	1.586126000
H	2.791477000	-2.102100000	2.488595000
H	2.226814000	-1.090565000	1.144899000
H	2.607423000	-2.794476000	0.874621000
C	0.259413000	-1.628254000	3.147096000
H	-0.732498000	-1.928863000	3.496947000
H	0.205673000	-0.565598000	2.887437000
H	0.958426000	-1.750613000	3.984214000
P	1.251646000	2.079486000	0.340084000
C	2.669591000	1.710231000	-0.837902000

H	2.793900000	2.516367000	-1.569590000
H	3.602092000	1.652458000	-0.264196000
C	0.487610000	3.636722000	-0.473983000
C	0.041565000	3.206653000	-1.879931000
H	-0.384032000	4.077635000	-2.394298000
H	-0.728641000	2.433231000	-1.840997000
H	0.864611000	2.833772000	-2.497823000
C	1.453440000	4.819515000	-0.599415000
H	1.017733000	5.562283000	-1.279731000
H	2.430785000	4.543971000	-1.007255000
H	1.609208000	5.321718000	0.358476000
C	-0.756171000	4.115874000	0.281491000
H	-0.529873000	4.471618000	1.288572000
H	-1.520047000	3.339722000	0.357314000
H	-1.202059000	4.954088000	-0.268648000
C	2.163799000	2.565574000	1.942548000
C	2.740572000	1.254081000	2.485204000
H	3.452018000	0.789903000	1.793752000
H	1.953222000	0.530059000	2.698633000
H	3.278513000	1.454584000	3.420342000
C	3.320289000	3.557659000	1.785890000
H	3.849265000	3.636113000	2.744598000
H	2.991763000	4.562078000	1.519158000
H	4.057278000	3.232389000	1.044708000
C	1.151952000	3.104167000	2.957434000
H	1.608452000	3.123572000	3.954977000
H	0.255688000	2.475654000	3.015118000
H	0.842625000	4.126466000	2.724673000
H	-1.288830000	1.339903000	0.688020000
B	-2.231745000	0.751513000	0.051626000
O	-3.316947000	0.369189000	0.865640000
O	-2.730244000	1.174578000	-1.198383000
C	-4.433896000	0.471562000	0.090881000
C	-4.077220000	0.961578000	-1.164929000
C	-5.740315000	0.151148000	0.406866000
C	-5.010174000	1.154409000	-2.165968000
H	-6.005310000	-0.234256000	1.386014000
C	-6.694526000	0.346685000	-0.598296000
C	-6.337517000	0.837099000	-1.855736000
H	-4.719624000	1.530031000	-3.141659000
H	-7.734219000	0.108508000	-0.394008000
H	-7.104422000	0.973520000	-2.612520000
H	-0.012293000	0.254345000	-1.554201000

TS2

Ni	0.343564000	0.087873000	-0.491986000
B	-0.755431000	-0.013284000	1.052180000
N	-1.670347000	1.013668000	1.491573000
N	-1.172203000	-1.230183000	1.708069000
C	-2.618373000	0.420186000	2.320469000
C	-3.750028000	0.953176000	2.919662000
H	-3.995092000	2.005379000	2.803852000
C	-4.571570000	0.101136000	3.667680000
H	-5.460358000	0.503342000	4.145608000
C	-4.265402000	-1.251314000	3.803913000
H	-4.916983000	-1.894128000	4.389148000
C	-3.128671000	-1.799254000	3.198041000
H	-2.894662000	-2.855357000	3.299549000
C	-2.308292000	-0.960607000	2.455566000
P	-0.514478000	-2.070320000	-0.707182000
C	-0.812841000	-2.495183000	1.106262000
H	-1.609911000	-3.234321000	1.236000000
H	0.102012000	-2.903543000	1.544230000
C	0.603535000	-3.466289000	-1.390300000
C	1.568284000	-3.916842000	-0.285758000
H	1.065739000	-4.548658000	0.454045000
H	2.049509000	-3.079450000	0.219505000
H	2.362437000	-4.522181000	-0.741727000
C	-0.109602000	-4.721822000	-1.894904000
H	0.653612000	-5.446317000	-2.207443000
H	-0.745099000	-4.533252000	-2.762992000
H	-0.708602000	-5.201028000	-1.115350000
C	1.404779000	-2.850608000	-2.544093000
H	2.034125000	-2.028263000	-2.196794000
H	0.756123000	-2.469554000	-3.339947000
H	2.057928000	-3.615160000	-2.984132000
C	-2.258648000	-2.242913000	-1.460530000
C	-3.006277000	-3.557462000	-1.208432000
H	-3.043508000	-3.822988000	-0.146952000
H	-2.600559000	-4.403652000	-1.760968000
H	-4.046353000	-3.429182000	-1.534465000
C	-3.111535000	-1.129222000	-0.839973000
H	-4.042944000	-1.027338000	-1.411124000
H	-2.592189000	-0.169611000	-0.851977000
H	-3.386428000	-1.355345000	0.194174000
C	-2.129291000	-2.003298000	-2.967650000
H	-1.625473000	-2.829666000	-3.476440000
H	-1.574065000	-1.086872000	-3.182552000
H	-3.127155000	-1.904146000	-3.413367000
P	-0.575931000	2.198997000	-0.640535000
C	-1.940713000	2.154649000	0.650383000

H	-1.997477000	3.096478000	1.207846000
H	-2.904512000	2.014169000	0.141802000
C	0.544766000	3.570224000	0.077141000
C	0.913602000	3.118370000	1.498271000
H	1.574943000	3.871869000	1.943966000
H	1.450002000	2.169215000	1.494665000
H	0.040372000	3.021348000	2.150725000
C	-0.120060000	4.945897000	0.172726000
H	0.559138000	5.626507000	0.702077000
H	-1.059468000	4.931458000	0.735238000
H	-0.309869000	5.387450000	-0.808683000
C	1.830727000	3.705569000	-0.748278000
H	1.670662000	4.250730000	-1.680196000
H	2.278861000	2.739372000	-0.979156000
H	2.568838000	4.270786000	-0.165321000
C	-1.459878000	2.890805000	-2.183510000
C	-2.095784000	1.693570000	-2.892235000
H	-2.882029000	1.225326000	-2.294890000
H	-1.343338000	0.935290000	-3.124008000
H	-2.554013000	2.026306000	-3.832394000
C	-2.560713000	3.916715000	-1.893574000
H	-3.004565000	4.236153000	-2.845303000
H	-2.194793000	4.811606000	-1.389581000
H	-3.372334000	3.496296000	-1.292129000
C	-0.426278000	3.494370000	-3.138737000
H	-0.899435000	3.669566000	-4.112917000
H	0.422386000	2.820634000	-3.300924000
H	-0.044148000	4.456069000	-2.789048000
H	1.716912000	0.162800000	-1.345310000
B	2.308407000	0.012087000	-0.167443000
O	3.052034000	-1.192080000	-0.007543000
O	3.138634000	1.084947000	0.268199000
C	4.229889000	-0.854347000	0.583259000
C	4.281050000	0.529114000	0.750711000
C	5.265549000	-1.676415000	0.982768000
C	5.369865000	1.153749000	1.327193000
H	5.216371000	-2.751969000	0.845858000
C	6.376126000	-1.053364000	1.567655000
C	6.426909000	0.329993000	1.736302000
H	5.397500000	2.231318000	1.452960000
H	7.212649000	-1.663416000	1.895963000
H	7.302307000	0.781055000	2.194450000
H	0.940148000	0.031435000	0.899205000

Complex **4**

Ni	0.020716000	-0.000020000	-0.000329000
B	-1.894745000	-0.000292000	-0.000374000
N	-2.779313000	1.126912000	-0.103526000
N	-2.779159000	-1.127541000	0.103603000
C	-4.095616000	0.707010000	-0.072005000
C	-5.289333000	1.412468000	-0.148124000
H	-5.290364000	2.492687000	-0.264854000
C	-6.486118000	0.693537000	-0.072386000
H	-7.430966000	1.226501000	-0.129856000
C	-6.486015000	-0.694355000	0.076066000
H	-7.430780000	-1.227364000	0.134474000
C	-5.289118000	-1.413226000	0.150620000
H	-5.289977000	-2.493443000	0.267373000
C	-4.095516000	-0.707709000	0.073308000
P	-0.415687000	-2.206388000	0.042333000
C	-2.255012000	-2.437464000	0.325538000
H	-2.673173000	-3.189612000	-0.354579000
H	-2.443402000	-2.774542000	1.350834000
C	0.294272000	-3.260750000	1.453776000
C	0.194382000	-2.368500000	2.698719000
H	-0.832931000	-2.036708000	2.891338000
H	0.822532000	-1.478509000	2.603785000
H	0.529683000	-2.934279000	3.576764000
C	-0.494139000	-4.553642000	1.710554000
H	-0.002122000	-5.091584000	2.530261000
H	-0.508058000	-5.223867000	0.849090000
H	-1.526002000	-4.378028000	2.025318000
C	1.756409000	-3.633839000	1.211298000
H	2.373681000	-2.770118000	0.959925000
H	1.855905000	-4.373128000	0.410301000
H	2.156289000	-4.091370000	2.125053000
C	-0.160046000	-2.988456000	-1.662181000
C	-0.479990000	-4.483546000	-1.700042000
H	-1.480941000	-4.717003000	-1.323054000
H	0.250367000	-5.070770000	-1.136911000
H	-0.438978000	-4.828130000	-2.740730000
C	-1.099610000	-2.231649000	-2.612337000
H	-0.939012000	-2.598724000	-3.633501000
H	-0.891349000	-1.156455000	-2.601023000
H	-2.158284000	-2.373667000	-2.374371000
C	1.276731000	-2.764335000	-2.139892000
H	2.019039000	-3.242299000	-1.499178000
H	1.522176000	-1.701494000	-2.188372000
H	1.378926000	-3.184076000	-3.148559000
P	-0.416064000	2.206382000	-0.042163000
C	-2.255538000	2.437091000	-0.324707000

H	-2.673738000	3.188639000	0.356056000
H	-2.444251000	2.774841000	-1.349715000
C	-0.159762000	2.987499000	1.662557000
C	-1.098392000	2.229714000	2.612836000
H	-0.936999000	2.595871000	3.634202000
H	-0.890007000	1.154556000	2.600360000
H	-2.157272000	2.371840000	2.375833000
C	-0.480295000	4.482433000	1.701645000
H	-0.438918000	4.826322000	2.742546000
H	-1.481517000	4.715742000	1.325277000
H	0.249531000	5.070359000	1.138546000
C	1.277497000	2.763574000	2.138998000
H	2.019134000	3.241873000	1.497750000
H	1.523206000	1.700756000	2.186940000
H	1.380499000	3.183060000	3.147688000
C	0.292753000	3.261984000	-1.453342000
C	0.192332000	2.370452000	-2.698779000
H	-0.834999000	2.038573000	-2.891183000
H	0.820591000	1.480463000	-2.604613000
H	0.527191000	2.936723000	-3.576673000
C	-0.496204000	4.554808000	-1.708719000
H	-0.005094000	5.093204000	-2.528674000
H	-0.509276000	5.224620000	-0.846921000
H	-1.528382000	4.379298000	-2.022494000
C	1.754908000	3.635577000	-1.211778000
H	2.153672000	4.094340000	-2.125405000
H	2.372920000	2.771953000	-0.961937000
H	1.854754000	4.374065000	-0.410080000
H	1.442165000	0.091421000	-1.031066000
B	2.206849000	0.000318000	-0.002352000
O	3.098909000	-1.162855000	-0.137354000
O	3.098878000	1.163500000	0.132480000
C	4.363910000	-0.696486000	-0.078359000
C	4.363905000	0.696956000	0.075390000
C	5.543508000	-1.412535000	-0.152278000
C	5.543486000	1.412860000	0.150883000
H	5.531943000	-2.492046000	-0.269721000
C	6.746594000	-0.691381000	-0.073853000
C	6.746586000	0.691548000	0.074115000
H	5.531898000	2.492379000	0.268246000
H	7.690395000	-1.226718000	-0.130387000
H	7.690377000	1.226764000	0.131954000
H	1.443215000	-0.090984000	1.027546000

Model Complex 3 cis

Ni	0.20553	1.21654	-0.12812
B	-1.06165	-0.30984	-0.04611
P	2.17869	2.07562	0.03692
P	-1.39265	2.69601	-0.12274
N	-1.76631	-0.95726	-1.13544
N	-1.84412	-0.70456	1.11248
C	2.86501	1.86794	1.72821
C	2.48997	3.87097	-0.24652
C	-2.99080	2.25863	-0.91670
H	-3.38995	1.35289	-0.45225
H	-3.72440	3.06660	-0.82170
C	-4.85434	-2.97853	-0.58051
H	-5.61882	-3.56503	-1.08246
C	3.51007	1.34434	-0.99753
H	3.22790	1.39949	-2.05309
H	4.46250	1.86588	-0.85361
C	-2.86978	-1.64317	-0.67140
C	-1.13334	4.39037	-0.79808
C	-3.83774	-2.38948	-1.33533
H	-3.79958	-2.51139	-2.41471
C	-3.93259	-2.07778	1.48077
H	-3.96707	-1.96136	2.56096
C	-2.91789	-1.48707	0.73563
C	-1.93476	3.06740	1.59374
C	-4.90099	-2.82537	0.80711
H	-5.70087	-3.29454	1.37319
H	-0.30810	4.87987	-0.27379
H	-2.03237	5.00903	-0.69855
H	-0.86807	4.32353	-1.85784
H	-2.26966	2.13949	2.06647
H	-2.75333	3.79506	1.61309
H	-1.09148	3.45968	2.16995
H	1.88915	4.46292	0.45028
H	2.19782	4.14131	-1.26585
H	3.54597	4.12533	-0.10027
H	2.23607	2.40420	2.44552
H	2.84708	0.80651	1.98924
H	3.89014	2.24787	1.79712
C	2.25307	-2.21064	0.67119
C	2.22000	-2.25765	-0.72429
C	3.01339	-3.09574	1.41196
C	2.94712	-3.19333	-1.43670
C	3.75419	-4.04687	0.69867
H	3.03070	-3.05001	2.49633
C	3.72141	-4.09471	-0.69493
H	2.91513	-3.22168	-2.52128

H	4.36506	-4.76025	1.24428
H	4.30708	-4.84507	-1.21812
B	0.92569	-0.56041	-0.05322
O	1.40646	-1.27532	-1.19063
O	1.46193	-1.19480	1.10754
H	-2.82865	2.04703	-1.97775
H	-1.64855	-0.51818	2.08203
H	-1.50678	-0.97625	-2.10769
H	3.64123	0.28866	-0.74453

Model Complex 3 trans

Ni	-0.06391	0.00737	-0.07630
B	1.93537	0.00869	-0.04860
P	-0.00832	2.13623	0.01861
P	-0.01330	-2.12276	-0.06487
N	2.87734	0.06142	-1.15510
N	2.82845	-0.05646	1.09886
C	0.81552	2.80988	1.51157
C	-1.58059	3.09129	0.02262
C	1.23290	-2.99475	-1.09238
H	2.23841	-2.69740	-0.78601
H	1.12818	-4.08112	-1.00151
C	6.57985	0.01682	-0.64593
H	7.53502	0.03775	-1.16344
C	0.89205	2.95420	-1.35414
H	0.43508	2.66767	-2.30632
H	0.85802	4.04442	-1.25670
C	4.18593	0.03065	-0.70662
C	-1.52584	-3.07384	-0.50213
C	5.39647	0.05764	-1.38895
H	5.42014	0.11115	-2.47452
C	5.33586	-0.08029	1.43975
H	5.31321	-0.13326	2.52544
C	4.15608	-0.04153	0.70647
C	0.34997	-2.74826	1.61856
C	6.55021	-0.05135	0.74779
H	7.48239	-0.08186	1.30514
H	-2.33684	-2.80991	0.18311
H	-1.35334	-4.15444	-0.44997
H	-1.84175	-2.81129	-1.51597
H	1.34279	-2.40418	1.92051
H	0.32193	-3.84252	1.64710
H	-0.38695	-2.34985	2.32174
H	-2.18299	2.80004	0.88851
H	-2.15213	2.85608	-0.88009
H	-1.39820	4.17074	0.06091
H	0.31308	2.42721	2.40519

H	1.85615	2.47653	1.53150
H	0.77909	3.90435	1.52432
C	-4.13748	-0.05452	0.69551
C	-4.16473	0.04448	-0.69509
C	-5.29736	-0.10683	1.44548
C	-5.35374	0.09285	-1.39822
C	-6.50808	-0.05816	0.74316
H	-5.26459	-0.18300	2.52784
C	-6.53549	0.03997	-0.64844
H	-5.36430	0.16866	-2.48105
H	-7.44302	-0.09731	1.29476
H	-7.49156	0.07538	-1.16286
B	-2.02243	-0.00274	-0.04312
O	-2.88722	0.07919	-1.16662
O	-2.84128	-0.08603	1.11585
H	1.10183	-2.71289	-2.14150
H	2.57737	-0.08787	2.07315
H	2.66878	0.10255	-2.13886
H	1.93154	2.61645	-1.35584

Model Complex trans 3·H2

Ni	0.03308	0.01583	-0.63162
B	1.96947	0.01502	-0.34092
O	2.59416	-0.02626	0.93796
O	3.00463	0.04688	-1.30984
C	3.94246	-0.02523	0.73034
C	4.96596	-0.06427	1.65802
H	4.75980	-0.09999	2.72314
C	6.27315	-0.05555	1.15632
H	7.10730	-0.08536	1.85130
C	6.52308	-0.00948	-0.21599
H	7.54923	-0.00385	-0.57198
C	5.47742	0.02984	-1.14645
H	5.66186	0.06494	-2.21543
C	4.19095	0.01978	-0.64064
B	-1.93085	0.00886	-0.32986
N	-3.02335	0.01815	-1.28500
N	-2.64225	-0.01190	0.93963
C	-4.25114	0.00131	-0.64792
C	-5.54902	-0.00016	-1.14488
H	-5.73289	0.01469	-2.21622
C	-6.60970	-0.02234	-0.23488
H	-7.63056	-0.02419	-0.60671
C	-6.37500	-0.04208	1.14084
H	-7.21428	-0.05937	1.83052
C	-5.07210	-0.04009	1.64707
H	-4.88893	-0.05540	2.71855

C	-4.01359	-0.01786	0.74736
P	0.02207	-2.04641	-0.07786
C	-1.40520	-3.10291	-0.55018
H	-1.25110	-4.14299	-0.24321
H	-2.31775	-2.72123	-0.08563
C	0.10705	-2.30243	1.73348
H	-0.77710	-1.86162	2.20166
H	0.15024	-3.36723	1.98616
H	0.99578	-1.79471	2.11739
C	1.41051	-3.10201	-0.66526
H	1.26927	-4.14986	-0.37878
H	1.48187	-3.03547	-1.75497
H	2.35498	-2.74582	-0.24397
P	0.01323	2.06729	-0.04260
C	-1.41846	3.12489	-0.49894
H	-1.26989	4.16045	-0.17449
H	-1.55083	3.10497	-1.58500
C	0.09296	2.29345	1.77293
H	-0.78731	1.83542	2.23205
H	0.12480	3.35412	2.04423
H	0.98609	1.78907	2.15123
C	1.39723	3.13969	-0.60975
H	1.46966	3.09274	-1.70045
H	1.25108	4.18160	-0.30466
H	2.34334	2.78012	-0.19507
H	0.45102	0.03330	-2.30835
H	-0.35731	0.02999	-2.31981
H	-1.53876	-3.06599	-1.63563
H	-2.32979	2.73144	-0.04176
H	-2.96158	0.03232	-2.28924
H	-2.24759	-0.02394	1.86527

Model Complex trans TS1

Ni	-0.05656	0.28183	-0.39645
B	-1.96717	-0.06333	-0.52770
O	-3.03348	0.80299	-0.19470
O	-2.53128	-1.32337	-0.86358
C	-4.18709	0.08466	-0.30353
C	-5.48765	0.48697	-0.06595
H	-5.71578	1.50002	0.24970
C	-6.48860	-0.47437	-0.25229
H	-7.52483	-0.20065	-0.07615
C	-6.18186	-1.77348	-0.65964
H	-6.98303	-2.49434	-0.79450
C	-4.86029	-2.16946	-0.89815
H	-4.61034	-3.17676	-1.21579
C	-3.88073	-1.21249	-0.71060

B	1.91308	-0.04375	-0.54148
N	2.57345	-1.23428	-1.04273
N	3.02502	0.72964	-0.02468
C	3.93864	-1.17739	-0.82779
C	4.95883	-2.07256	-1.12634
H	4.74370	-3.01269	-1.62763
C	6.26516	-1.73256	-0.76563
H	7.07584	-2.42002	-0.99035
C	6.54167	-0.52556	-0.12068
H	7.56498	-0.28354	0.15236
C	5.52065	0.38006	0.17831
H	5.73627	1.32077	0.67854
C	4.21999	0.04933	-0.18284
P	-0.00811	2.48098	-0.04516
C	1.09247	3.42489	-1.17952
H	1.10937	4.49554	-0.94532
H	2.11040	3.02764	-1.14143
C	0.59134	3.05569	1.60178
H	1.57391	2.63313	1.83086
H	0.65727	4.14862	1.65591
H	-0.10504	2.71023	2.37244
C	-1.53574	3.49416	-0.18150
H	-1.32813	4.55540	-0.00210
H	-1.96335	3.37619	-1.18106
H	-2.27904	3.13941	0.53532
P	-0.03844	-1.03280	1.29933
C	0.02435	-2.82335	0.91535
H	-0.03830	-3.43512	1.82180
H	-0.80957	-3.06564	0.25077
C	1.33399	-0.85747	2.50810
H	2.28717	-1.07809	2.02129
H	1.19971	-1.53012	3.36207
H	1.36804	0.17525	2.86743
C	-1.48428	-0.96377	2.43679
H	-2.38904	-1.28567	1.91296
H	-1.33649	-1.61076	3.30825
H	-1.63616	0.06465	2.77719
H	-0.49916	0.00458	-1.81378
H	0.54539	0.04894	-1.75593
H	0.73057	3.29237	-2.20421
H	0.96143	-3.04435	0.39723
H	2.14874	-2.01222	-1.52062
H	3.00152	1.61441	0.45175

Model Complex trans Int 1

Ni	-0.38282	-1.19666	-0.44031
B	1.50654	-0.75226	-0.70745

O	2.58651	-1.25530	0.07497
O	1.95352	0.44937	-1.33901
C	3.59518	-0.34771	-0.02127
C	4.83077	-0.35865	0.59786
H	5.11899	-1.16718	1.26204
C	5.68626	0.71646	0.32428
H	6.66709	0.74377	0.78982
C	5.30279	1.74927	-0.53069
H	5.98953	2.56861	-0.72176
C	4.04715	1.75234	-1.15192
H	3.74066	2.55029	-1.82101
C	3.21208	0.68647	-0.87725
B	-1.33674	0.67968	-0.99806
N	-0.58165	1.89985	-1.06510
N	-2.64152	1.09624	-0.54366
C	-1.34972	2.95560	-0.61909
C	-1.04766	4.30224	-0.44821
H	-0.05893	4.68199	-0.69132
C	-2.04629	5.14881	0.03767
H	-1.82863	6.20427	0.17476
C	-3.31669	4.65928	0.34888
H	-4.07710	5.33790	0.72489
C	-3.62615	3.30677	0.18662
H	-4.61398	2.92568	0.43267
C	-2.63743	2.45821	-0.29793
P	-1.61357	-3.02014	-0.61556
C	-2.70021	-3.14237	-2.09439
H	-3.22442	-4.10312	-2.15241
H	-3.43914	-2.33496	-2.07205
C	-2.79925	-3.49832	0.70975
H	-3.56592	-2.72434	0.81207
H	-3.29326	-4.45176	0.48986
H	-2.27622	-3.59013	1.66647
C	-0.62680	-4.56385	-0.76740
H	-1.25777	-5.45245	-0.88332
H	0.03897	-4.47829	-1.63115
H	-0.00016	-4.68010	0.12211
P	-0.21845	-0.60633	1.60139
C	0.74595	0.89509	2.03372
H	0.66917	1.12680	3.10141
H	1.80084	0.74560	1.78682
C	-1.78378	-0.26731	2.50442
H	-2.26126	0.61808	2.07640
H	-1.59968	-0.09249	3.57024
H	-2.46674	-1.11363	2.39381
C	0.57757	-1.86231	2.67878
H	1.56938	-2.07907	2.27299

H	0.67166	-1.51471	3.71351
H	-0.00727	-2.78688	2.66264
H	0.82372	-1.63008	-1.41504
H	-1.12524	-0.35235	-1.67805
H	-2.09210	-3.01006	-2.99430
H	0.37370	1.74401	1.45449
H	0.39477	1.98096	-1.30235
H	-3.46120	0.53451	-0.38487

Model Complex trans Int 1'

Ni	-0.08177	-0.89459	-0.02138
B	1.53269	0.42276	0.76097
N	2.89696	0.09093	1.08002
N	1.60528	1.74766	0.21746
C	3.72793	1.13512	0.71009
C	5.10752	1.28180	0.79315
H	5.72566	0.49378	1.21544
C	5.67556	2.46652	0.32015
H	6.75205	2.60093	0.37676
C	4.88032	3.47763	-0.22467
H	5.34579	4.38938	-0.58834
C	3.49421	3.33687	-0.31078
H	2.87538	4.12407	-0.73281
C	2.92044	2.16205	0.16314
P	1.25802	-2.31800	-0.99792
C	2.29488	-1.63541	-2.35108
H	2.92226	-2.39927	-2.82435
H	1.64345	-1.18578	-3.10647
C	0.38745	-3.68765	-1.86684
H	-0.36863	-3.26667	-2.53592
H	1.07589	-4.31086	-2.44931
H	-0.12260	-4.32041	-1.13383
C	2.51191	-3.27492	-0.04828
H	2.99091	-4.03851	-0.67096
H	3.28511	-2.59703	0.32302
H	2.03840	-3.75961	0.81089
P	-1.57850	-2.00674	1.04472
C	-2.55787	-1.10992	2.31495
H	-3.25948	-1.77115	2.83563
H	-1.87773	-0.66100	3.04520
C	-2.91069	-2.79521	0.05715
H	-3.45060	-2.01476	-0.48496
H	-3.60934	-3.35609	0.68809
H	-2.46879	-3.47291	-0.67922
C	-0.97618	-3.44138	2.03225
H	-0.22643	-3.10080	2.75304
H	-1.79135	-3.93583	2.57327

H	-0.50312	-4.17200	1.36894
H	-0.35124	0.08138	-1.24720
B	-1.39580	0.46521	-0.52744
O	-1.36711	1.77893	0.03166
O	-2.69279	0.25711	-1.07469
C	-2.62303	2.28544	-0.09952
C	-3.42677	1.36239	-0.77087
C	-3.11619	3.50048	0.33562
C	-4.75907	1.61572	-1.03667
H	-2.48253	4.20903	0.85951
C	-4.46528	3.76767	0.06938
C	-5.26863	2.84599	-0.60159
H	-5.37551	0.89135	-1.55947
H	-4.89173	4.71201	0.39493
H	-6.31113	3.08409	-0.79171
H	0.53154	-0.10325	1.30039
H	-3.12053	-0.30047	1.83991
H	2.93238	-0.84391	-1.94606
H	3.25393	-0.72437	1.54880
H	0.83467	2.31099	-0.10580

Model Complex trans TS2

Ni	-0.09092	-1.05390	-0.15123
B	1.28598	0.29225	0.13597
N	2.39433	0.22264	1.06738
N	1.48537	1.55908	-0.52243
C	3.16959	1.36803	0.97757
C	4.31397	1.75657	1.66176
H	4.74932	1.11631	2.42486
C	4.88612	2.99302	1.34899
H	5.78001	3.31506	1.87580
C	4.32319	3.81670	0.37300
H	4.78389	4.77442	0.14725
C	3.17364	3.42931	-0.32102
H	2.73520	4.07161	-1.08030
C	2.59910	2.20117	-0.01497
P	1.40497	-2.20370	-1.16081
C	2.33631	-1.35081	-2.48750
H	3.00432	-2.03884	-3.01634
H	1.62646	-0.91688	-3.19817
C	0.73378	-3.65309	-2.07113
H	-0.04685	-3.31373	-2.75849
H	1.51434	-4.16858	-2.64132
H	0.28401	-4.35978	-1.36853
C	2.74673	-2.95701	-0.16811
H	3.39207	-3.59067	-0.78535
H	3.34832	-2.15925	0.27468

H	2.31686	-3.55887	0.63747
P	-1.34500	-2.50380	1.00408
C	-2.01772	-1.75417	2.53660
H	-2.63274	-2.45923	3.10688
H	-1.18952	-1.40818	3.16208
C	-2.86221	-3.16328	0.21045
H	-3.48582	-2.31542	-0.08665
H	-3.43120	-3.81603	0.88165
H	-2.59349	-3.72078	-0.69208
C	-0.57400	-4.03698	1.66617
H	0.29773	-3.77854	2.27512
H	-1.27810	-4.60337	2.28602
H	-0.24166	-4.68015	0.84588
H	-1.21452	-0.66121	-1.43554
B	-1.56723	0.27626	-0.65437
O	-1.37474	1.61100	-1.15749
O	-2.88842	0.21241	-0.08813
C	-2.45185	2.32744	-0.74390
C	-3.36456	1.48473	-0.10155
C	-2.69862	3.67763	-0.90009
C	-4.55631	1.96178	0.40910
H	-1.98321	4.32308	-1.39982
C	-3.90600	4.17046	-0.38554
C	-4.81501	3.33103	0.25497
H	-5.25735	1.29818	0.90572
H	-4.13312	5.22761	-0.48851
H	-5.74171	3.74207	0.64501
H	-0.55800	0.19048	0.54949
H	-2.61972	-0.88460	2.25930
H	2.92131	-0.53382	-2.05751
H	2.59332	-0.49060	1.74857
H	0.84275	2.00441	-1.15820

Model Complex trans 4

Ni	-0.07307	-1.04249	-0.12678
B	1.34645	0.27204	0.10329
N	2.47795	0.19619	1.00531
N	1.51634	1.55110	-0.53755
C	3.22974	1.35856	0.92993
C	4.37600	1.75469	1.60663
H	4.83561	1.10628	2.34838
C	4.91711	3.01013	1.31557
H	5.81147	3.33900	1.83736
C	4.32210	3.84455	0.36799
H	4.75845	4.81748	0.15948
C	3.17081	3.44936	-0.31845
H	2.70667	4.10065	-1.05439

C	2.62683	2.20225	-0.03376
P	1.39461	-2.23120	-1.12246
C	2.37349	-1.41860	-2.43968
H	2.99332	-2.14159	-2.98038
H	1.69025	-0.93161	-3.14174
C	0.65641	-3.63910	-2.04474
H	-0.09556	-3.25591	-2.74070
H	1.41602	-4.19374	-2.60620
H	0.16108	-4.32170	-1.34939
C	2.69127	-3.05480	-0.12576
H	3.30472	-3.71604	-0.74652
H	3.33451	-2.29208	0.31981
H	2.23187	-3.63887	0.67639
P	-1.36577	-2.50372	0.97969
C	-2.07030	-1.75921	2.49916
H	-2.70107	-2.46558	3.05003
H	-1.25496	-1.42176	3.14579
C	-2.86239	-3.15001	0.13908
H	-3.47727	-2.29642	-0.15992
H	-3.44817	-3.81208	0.78619
H	-2.57173	-3.69363	-0.76521
C	-0.61246	-4.04028	1.65151
H	0.24442	-3.78324	2.28157
H	-1.33228	-4.60637	2.25307
H	-0.26166	-4.68289	0.83877
H	-1.20226	-0.60495	-1.42078
B	-1.54891	0.31081	-0.61387
O	-1.35705	1.65885	-1.09934
O	-2.89299	0.23267	-0.08715
C	-2.45142	2.35879	-0.70791
C	-3.37570	1.50105	-0.10092
C	-2.70623	3.70858	-0.85534
C	-4.58530	1.96388	0.38017
H	-1.98211	4.36550	-1.32691
C	-3.93214	4.18658	-0.37025
C	-4.85184	3.33294	0.23429
H	-5.29475	1.28893	0.84890
H	-4.16451	5.24314	-0.46789
H	-5.79294	3.73149	0.60232
H	-0.62800	0.19214	0.55780
H	-2.66165	-0.88547	2.21263
H	3.01332	-0.64737	-2.00337
H	2.69050	-0.51843	1.68087
H	0.84694	2.00495	-1.13930

XV- REFERENCES

1. Y. Segawa, M. Yamashita, K. Nozaki, *J. Am. Chem. Soc.* 2009, **131***,* 9201- 9203.

2. N. Curado, C. Maya, J. López-Serrano, A. Rodríguez, *Chem. Commun.* 2014, **50***,* 15718-15721

3. (a) X. He, J. F. Hartwig, *Organometallics* 1996, **15**, 400-407. (b) F. D. Lewis, B. E. Zebrowski, P. E. Correa, J. *Am. Chem. Soc.* 1984, **106**, 187-193.

4. P. Ríos, H. Fouilloux, P. Vidossich, J. Díez, A. Lledós, S. Conejero, *Angew. Chem. Int. Ed.* 2018, ***57***, 3217-3221.

5 G. M. Sheldrick, Crystal structure refinement with SHELXL. Acta Cryst. 2008, **A64**, 112-122.

6. O.V. Dolomanov, L. J. Bourhis, R. J. Gildea, J. A. K. Howard, H. J. Puschmann, Appl. Cryst. 2009, **42**, 339-341.

7. P. T. Beurskens, G. Beurskens, R. de Gelder, S. García-Granda, R. O. Gould, J. M. M. Smits, 2008. *The DIRDIF2008 program system*. Crystallography Laboratory, University of Nijmegen: Nijmegen, The Netherlands.

8. G. M. Sheldrick, *Acta Cryst.* 2015, C71, 3-8**.**

9. A. L. Spek, *Acta Cryst.* 2009, **D65**, 148-155.

10. C. Adamo, V. Barone, *J. Chem. Phys.,* 1999, **110**, 6158-6170.

11. S. Grimme, J. Anthony, S. Ehrlich, H. Krieg, *J. Chem. Phys*. 2010, **132**, 154104.

12. Gaussian 09, Revision D.01, M. J. Frisch, G. W. Trucks, H. B. Schlegel, G. E. Scuseria, M. A. Robb, J. R. Cheeseman, G. Scalmani, V. Barone, B. Mennucci, G. A. Petersson, H. Nakatsuji, M. Caricato, X. Li, H. P. Hratchian, A. F. Izmaylov, J. Bloino, G. Zheng, J. L. Sonnenberg, M. Hada, M. Ehara, K. Toyota, R. Fukuda, J. Hasegawa, M. Ishida, T. Nakajima, Y. Honda, O. Kitao, H. Nakai, T. Vreven, J. A. Montgomery, Jr., J. E. Peralta, F. Ogliaro, M. Bearpark, J. J. Heyd, E. Brothers, K. N. Kudin, V. N. Staroverov, R. Kobayashi, J. Normand, K. Raghavachari, A. Rendell, J. C. Burant, S. S. Iyengar, J. Tomasi, M. Cossi, N. Rega, J. M. Millam, M. Klene, J. E. Knox, J. B. Cross, V. Bakken, C. Adamo, J. Jaramillo, R. Gomperts, R. E. Stratmann, O. Yazyev, A. J. Austin, R. Cammi, C. Pomelli, J. W. Ochterski, R. L. Martin, K. Morokuma, V. G. Zakrzewski, G. A. Voth, P. Salvador, J. J. Dannenberg, S. Dapprich, A. D. Daniels, Ö. Farkas, J. B. Foresman, J. V. Ortiz, J. Cioslowski, and D. J. Fox, Gaussian, Inc., Wallingford CT, 2009.

13. S. A. V. Marenich, C. J. Cramer, D. G. Truhlar, Universal Solvation Model Based on Solute Electron Density and on a Continuum Model of the Solvent Defined by the Bulk Dielectric Constant and Atomic Surface Tensions. *J. Phys. Chem. B*, 2009, **113**, 6378–6396.

14. a) W. J. Hehre, R. Ditchfield, J. A. Pople, Self—Consistent Molecular Orbital Methods. XII. Further Extensions of Gaussian—Type Basis Sets for Use in Molecular Orbital Studies of Organic Molecules. *J. Chem. Phys.* 1972, **56**, 2257-2261; b) P. C. Hariharan, J. A. Pople, The influence of polarization functions on molecular orbital hydrogenation energies. *Theor. Chim. Acta.* 1973, **28**, 213-222; c) M. M. Francl, W. J. Pietro, W.H. Hehre, J. S. Binkley, M. S. Gordon, D. J. DeFrees, J. A. Pople, Self-consistent molecular orbital methods. XXIII. A polarization-type basis set for second-row elements. *J. Chem. Phys.* 1982, **77**, 3654-3665; d) M. Dolg, U. Wedig, H. Stoll, H. Preuss, Energy-adjusted ab initio pseudopotentials for the first row transition elements. *J. Chem. Phys.,* 1987, **86**, 866-872.

15. F. Weigend, R. Ahlrichs, Balanced basis sets of split valence, triple zeta valence and quadruple zeta valence quality for H to Rn: Design and assessment of accuracy. *Phys. Chem. Chem. Phys.,* 2005, **7**, 3297–3305.
16. S. Grimme, Supramolecular Binding Thermodynamics by Dispersion-Corrected Density Functional Theory. *Chem. Eur. J.*, 2012, **18**, 9955−9964.
17. G. Luchini, J. V. Alegre-Requena, I. Funes-Ardoiz, R. S. Paton, GoodVibes: Automated Thermochemistry for Heterogeneous Computational Chemistry Data. *F1000Research*, 2020, **9**, 291 **DOI:** 10.12688/f1000research.22758.1
18. R. F. W. Bader, Atoms in Molecules: A Quantum Theory; Oxford University Press: Oxford, U.K., 1995.
19. T., Lu, F. Chen, *J. Comput. Chem.*, 2012, **33**, 580-592.
20. Chemcraft - graphical software for visualization of quantum chemistry computations. https://www.chemcraftprog.com
21. CYLview, version 1.0b; C. Y. Legault, Université de Sherbrooke, 2009 (http://www.cylview.org).
22. www.cp2k.org
23. J. P. Perdew, K. Burke, M. Ernzerhof, Generalized Gradient Approximation Made Simple. *Phys. Rev. Lett.*, 1996, ***77***, 3865−3868.
24. G. Lippert, J. Hutter, M. Parrinello, A hybrid Gaussian and plane wave density functional scheme. *Mol. Phys.*, 1997, **92**, 477−488
25. (a) S. Goedecker, M. Teter, J. Hutter. Separable dual-space Gaussian pseudopotentials. *Phys. Rev. B Condens. Matter.*, 1996, **54**, 1703-1710; (b) M. Krack, Pseudopotentials for H to Kr optimized for gradient-corrected exchange-correlation functionals. *Theor. Chem. Acc.*, 2005, **114**, 145−152.
